# The blood metabolome of incident kidney cancer: A case–control study nested within the MetKid consortium

**DOI:** 10.1371/journal.pmed.1003786

**Published:** 2021-09-20

**Authors:** Florence Guida, Vanessa Y. Tan, Laura J. Corbin, Karl Smith-Byrne, Karine Alcala, Claudia Langenberg, Isobel D. Stewart, Adam S. Butterworth, Praveen Surendran, David Achaintre, Jerzy Adamski, Pilar Amiano, Manuela M. Bergmann, Caroline J. Bull, Christina C. Dahm, Audrey Gicquiau, Graham G. Giles, Marc J. Gunter, Toomas Haller, Arnulf Langhammer, Tricia L. Larose, Börje Ljungberg, Andres Metspalu, Roger L. Milne, David C. Muller, Therese H. Nøst, Elin Pettersen Sørgjerd, Cornelia Prehn, Elio Riboli, Sabina Rinaldi, Joseph A. Rothwell, Augustin Scalbert, Julie A. Schmidt, Gianluca Severi, Sabina Sieri, Roel Vermeulen, Emma E. Vincent, Melanie Waldenberger, Nicholas J. Timpson, Mattias Johansson

**Affiliations:** 1 Genomic Epidemiology Branch, International Agency for Research on Cancer (IARC/WHO), Lyon, France; 2 MRC Integrative Epidemiology Unit, University of Bristol, Bristol, United Kingdom; 3 Population Health Sciences, Bristol Medical School, University of Bristol, Bristol, United Kingdom; 4 MRC Epidemiology Unit, University of Cambridge, Cambridge, United Kingdom; 5 British Heart Foundation Cardiovascular Epidemiology Unit, Department of Public Health and Primary Care, University of Cambridge, Cambridge, United Kingdom; 6 British Heart Foundation Centre of Research Excellence, University of Cambridge, Cambridge, United Kingdom; 7 Health Data Research UK Cambridge, Wellcome Genome Campus and University of Cambridge, Cambridge, United Kingdom; 8 National Institute for Health Research Blood and Transplant Research Unit in Donor Health and Genomics, University of Cambridge, Cambridge, United Kingdom; 9 Rutherford Fund Fellow, Department of Public Health and Primary Care, University of Cambridge, Cambridge, United Kingdom; 10 Nutrition and Metabolism Branch, International Agency for Research on Cancer (IARC/WHO), Lyon, France; 11 Institute of Experimental Genetics, Helmholtz Zentrum München, German Research Center for Environmental Health (GmbH), Neuherberg, Germany; 12 Institute of Biochemistry, Faculty of Medicine, University of Ljubljana, Ljubljana, Slovenia; 13 Chair of Experimental Genetics, School of Life Science, Weihenstephan, Technische Universität München, Freising, Germany; 14 Department of Biochemistry, Yong Loo Lin School of Medicine, National University of Singapore, Singapore, Singapore; 15 Ministry of Health of the Basque Government, Sub Directorate for Public Health and Addictions of Gipuzkoa, San Sebastián, Spain; 16 Biodonostia Health Research Institute, Epidemiology of Chronic and Communicable Diseases Group, San Sebastián, Spain; 17 Spanish Consortium for Research on Epidemiology and Public Health (CIBERESP), Instituto de Salud Carlos III, Madrid, Spain; 18 German Institute of Human Nutrition Potsdam-Rehbrücke, Nuthetal, Germany; 19 School of Cellular and Molecular Medicine, University of Bristol, Bristol, United Kingdom; 20 Bristol Renal, Translational Health Sciences, Bristol Medical School, University of Bristol, Bristol, United Kingdom; 21 Department of Public Health, Aarhus University, Aarhus, Denmark; 22 Cancer Epidemiology Division, Cancer Council Victoria, Melbourne, Australia; 23 Centre for Epidemiology and Biostatistics, Melbourne School of Population and Global Health, The University of Melbourne, Melbourne, Australia; 24 Precision Medicine, School of Clinical Sciences at Monash Health, Monash University, Clayton, Australia; 25 Institute of Genomics, University of Tartu, Tartu, Estonia; 26 HUNT Research Centre, Department of Public Health and Nursing, NTNU, Norwegian University of Science and Technology, Levanger, Norway; 27 Levanger Hospital, Nord-Trøndelag Hospital Trust, Levanger, Norway; 28 Department of Community Medicine and Global Health, Institute of Health and Society, University of Oslo, Oslo, Norway; 29 Department of Surgical and Perioperative Sciences, Urology and Andrology, Umeå University, Umeå, Sweden; 30 Department of Epidemiology and Biostatistics, School of Public Health, Imperial College London, London, United Kingdom; 31 Department of Community Medicine, Faculty of Health Sciences, UiT The Arctic University of Norway, Tromsø, Norway; 32 Metabolomics and Proteomics Core (MPC), Helmholtz Zentrum München, German Research Center for Environmental Health (GmbH), Neuherberg, Germany; 33 Université Paris-Saclay, UVSQ, Inserm, Gustave Roussy, Équipe “Exposome et Hérédité”, CESP UMR1018, Inserm, Villejuif, France; 34 Cancer Epidemiology Unit, Nuffield Department of Population Health, University of Oxford, Oxford, United Kingdom; 35 Department of Statistics, Computer Science and Applications (DISIA), University of Florence, Florence, Italy; 36 Epidemiology and Prevention Unit, Fondazione IRCCS Istituto Nazionale dei Tumori di Milano, Milano, Italy; 37 Institute for Risk Assessment Sciences (IRAS), Utrecht University, Utrecht, the Netherlands; 38 Research Unit Molecular Epidemiology, Institute of Epidemiology, Helmholtz Zentrum München, German Research Center for Environmental Health (GmbH), Neuherberg, Germany; Royal Derby Hospital, UNITED KINGDOM

## Abstract

**Background:**

Excess bodyweight and related metabolic perturbations have been implicated in kidney cancer aetiology, but the specific molecular mechanisms underlying these relationships are poorly understood. In this study, we sought to identify circulating metabolites that predispose kidney cancer and to evaluate the extent to which they are influenced by body mass index (BMI).

**Methods and findings:**

We assessed the association between circulating levels of 1,416 metabolites and incident kidney cancer using pre-diagnostic blood samples from up to 1,305 kidney cancer case–control pairs from 5 prospective cohort studies. Cases were diagnosed on average 8 years after blood collection. We found 25 metabolites robustly associated with kidney cancer risk. In particular, 14 glycerophospholipids (GPLs) were inversely associated with risk, including 8 phosphatidylcholines (PCs) and 2 plasmalogens. The PC with the strongest association was PC ae C34:3 with an odds ratio (OR) for 1 standard deviation (SD) increment of 0.75 (95% confidence interval [CI]: 0.68 to 0.83, *p* = 2.6 × 10^−8^). In contrast, 4 amino acids, including glutamate (OR for 1 SD = 1.39, 95% CI: 1.20 to 1.60, *p* = 1.6 × 10^−5^), were positively associated with risk. Adjusting for BMI partly attenuated the risk association for some—but not all—metabolites, whereas other known risk factors of kidney cancer, such as smoking and alcohol consumption, had minimal impact on the observed associations. A mendelian randomisation (MR) analysis of the influence of BMI on the blood metabolome highlighted that some metabolites associated with kidney cancer risk are influenced by BMI. Specifically, elevated BMI appeared to decrease levels of several GPLs that were also found inversely associated with kidney cancer risk (e.g., −0.17 SD change [ß_BMI_] in 1-(1-enyl-palmitoyl)-2-linoleoyl-GPC (P-16:0/18:2) levels per SD change in BMI, *p* = 3.4 × 10^−5^). BMI was also associated with increased levels of glutamate (ß_BMI_: 0.12, *p* = 1.5 × 10^−3^). While our results were robust across the participating studies, they were limited to study participants of European descent, and it will, therefore, be important to evaluate if our findings can be generalised to populations with different genetic backgrounds.

**Conclusions:**

This study suggests a potentially important role of the blood metabolome in kidney cancer aetiology by highlighting a wide range of metabolites associated with the risk of developing kidney cancer and the extent to which changes in levels of these metabolites are driven by BMI—the principal modifiable risk factor of kidney cancer.

## Abstract 2

In a case-control study, Florence Guida and colleagues identify metabolites associated with risk of kidney cancer, and use Mendelian randomization techniques to study the role of body mass index in this relationship.

## Author summary

**Why was this study done?:**

- Several modifiable risk factors have been established for kidney cancer, among which elevated body mass index (BMI) and obesity are central.
- The biological mechanisms underlying these relationships are poorly understood, but obesity-related metabolic perturbations may be important.

**What did the researchers do and find?:**

- We looked at the association between kidney cancer and the levels of 1,416 metabolites measured in blood on average 8 years before the disease onset. The study included 1,305 kidney cancer cases and 1,305 healthy controls.
- We found 25 metabolites robustly associated with kidney cancer risk.
- Specifically, multiple glycerophospholipids (GPLs) were inversely associated with risk, while several amino acids were positively associated with risk.
- Accounting for BMI highlighted that some—but not all—metabolites associated with kidney cancer risk are influenced by BMI.

**What do these findings mean?:**

- These findings illustrate the potential utility of prospectively measured metabolites in helping us to understand the aetiology of kidney cancer.
- By examining overlap between the metabolomic profile of prospective risk of kidney cancer and that of modifiable risk factors for the disease—in this case BMI—we can begin to identify biological pathways relevant to disease onset.

## Introduction

Kidney cancer is the 14th most common cancer worldwide, with renal cell carcinoma (RCC) making up the majority of cases [1]. There are important geographical variations in kidney cancer incidence that are only partly understood [2]. Excess bodyweight and related conditions, such as hypertension, diabetes, and related metabolic perturbations, are among the most robustly implicated risk factors for kidney cancer, with support from both traditional observational studies and genetic studies [2–7]. For instance, in the United Kingdom, an estimated 24% of kidney cancer cases are attributable to overweight and obesity, making this the leading modifiable risk factor for the disease [8]. Germline mutations responsible for an inherited predisposition to kidney cancer (a small proportion of kidney cancer cases) have a key role in regulating cellular metabolism [9], and this, together with evidence of extensive metabolic reprogramming within tumours themselves [10], have led to the characterisation of kidney cancer as a metabolic disease. However, the molecular mechanisms predisposing kidney cancer remain largely unknown. Given the likely metabolic underpinnings of kidney cancer, studies of circulating metabolites, the downstream products of cellular regulatory processes, may improve our understanding into pathways relevant to kidney cancer aetiology [11].

Metabolite variations are the result of genetic and nongenetic factors and provide a readout of physiological functions [12]. Metabolomics technologies based on mass spectrometry (MS) and nuclear magnetic resonance (NMR) have enabled the systematic quantification of hundreds of metabolites (the “metabolome”) from a single biological sample. The analysis of metabolites has enabled a more thorough exploration of an individual’s metabolic status, providing important insights into the biological pathways leading to diseases such as cancer [11,13,14] and has enabled the discovery and development of new drug targets [15]. Already, global metabolic profiling of blood [16–19], urine [20–24], and tissue samples [24–27] has been used to characterise kidney cancer and identify novel potential diagnostic biomarkers. However, because of the cross-sectional or retrospective design of these studies, they could not inform the identification of biomarkers for incident disease development. Prospective cohort studies, where healthy individuals initially donate blood at recruitment and are longitudinally followed over time for incident disease, can circumvent many of the problems of retrospective study designs—particularly where the focus is on identifying risk factors for disease onset.

The aim of this study was to identify circulating metabolites associated with the development of kidney cancer in a prospective case–control framework. We used 2 complementary metabolomics platforms [28] to quantify over 1,000 metabolites in blood samples donated by research participants later diagnosed with kidney cancer along with matched control participants. In a series of follow-up analyses, including a 2-sample mendelian randomisation (MR) analysis, which uses genetic variants as proxies for an exposure of interest [29], we evaluated the extent to which the metabolomic signature of disease risk could be explained by body mass index (BMI), the leading modifiable risk factor for kidney cancer.

## Methods

### Analytical strategy (Fig 1)

The primary analysis was predefined and involved investigating the association between circulating levels of metabolites and kidney cancer risk using pre-diagnostic metabolomics measurements in a case–control study nested within multiple large-scale prospective cohorts (the MetKid consortium). Adjustment for known risk factors for kidney cancer (BMI, hypertension, alcohol consumption, and smoking) [2] was then carried out to evaluate the extent to which these could explain the associations between blood metabolites and kidney cancer risk.

A natural complementary analysis would have been to interrogate the potentially causal role for the identified risk-associated metabolites in kidney cancer aetiology through MR analyses. However, given the methodological constraints of MR in this context, specifically, widespread pleiotropic instruments, which would violate the MR assumptions, we chose not to pursue this analysis. Our analysis plan was therefore revised, and as a secondary analysis, we rather used a 2-sample MR approach to estimate the causal effect of BMI on the blood metabolome. This analysis complemented the main risk analysis by quantifying the extent to which BMI—the central risk factor of kidney cancer—influenced the identified risk metabolites. This study is reported as per the Strengthening the Reporting of Observational Studies in Epidemiology (STROBE) and STROBE-MR guidelines (S1 and S2 Tables) [30,31].

**Fig 1:**
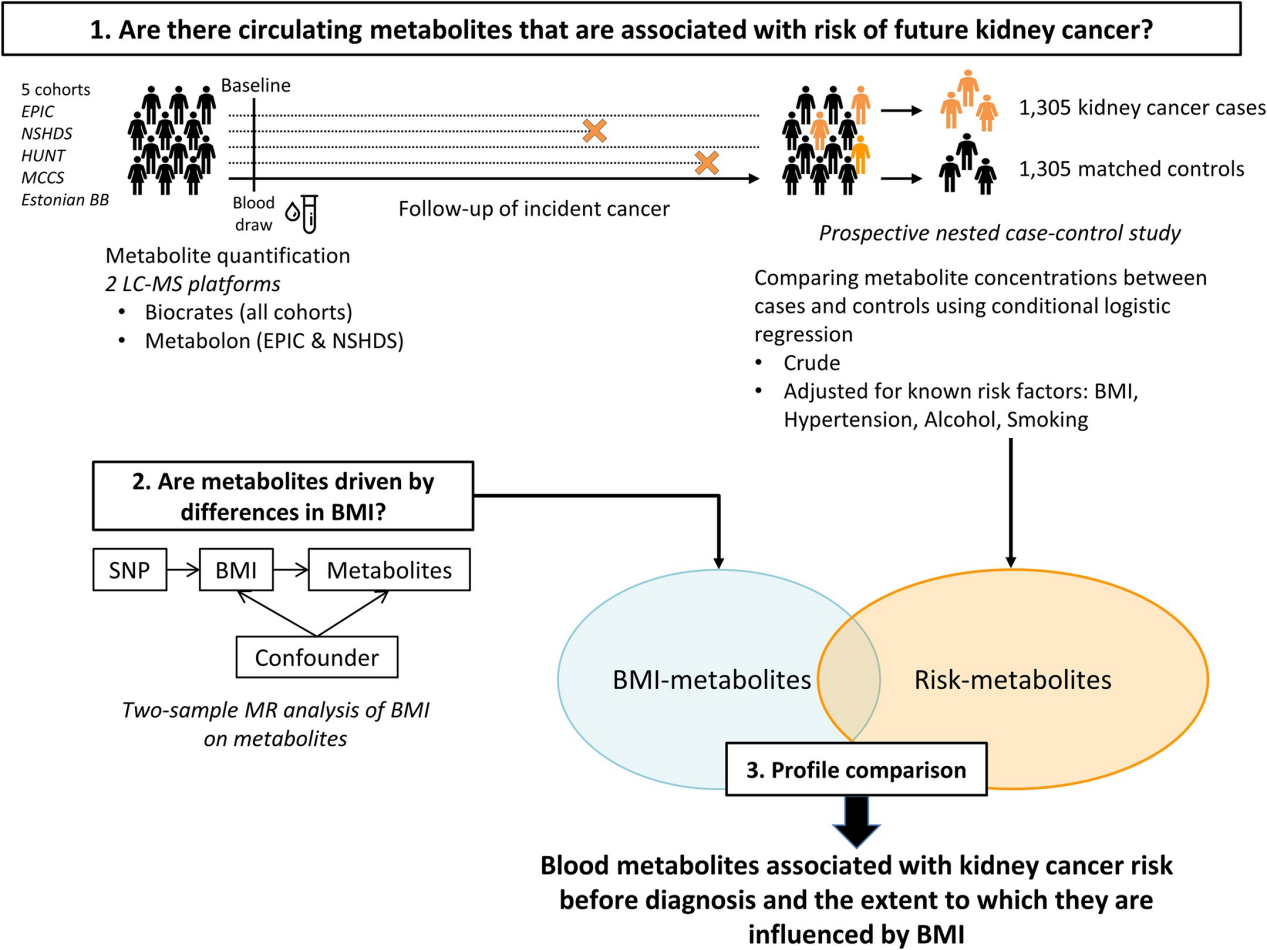
Conceptual framework of the study design. This study includes 3 main analytical steps: (i) the investigation of the associations between circulating levels of metabolites and kidney cancer risk using pre-diagnostic measurements in a case–control study nested within multiple large-scale prospective cohorts; (ii) the assessment of the causal effect of BMI, the leading modifiable risk factor for kidney cancer, on circulating metabolites levels; and (iii) the evaluation of the overlap between the metabolic footprint of BMI and that of kidney cancer risk. The orange X’s indicate the time at which a participant is diagnosed with kidney cancer when his follow-up is stopped. Controls have been selected among participants free of cancer at the time their matched case was diagnosed. Metabolites from all samples have been measured on the Biocrates platform, while only samples from EPIC and NSHDS cohorts have been measured with Metabolon platform. BMI, body mass index; EPIC, The European Prospective Investigation into Cancer and Nutrition; Estonian BB, University of Tartu—Estonian Biobank; HUNT, The Trøndelag Health Study; LC–MS, liquid chromatography–tandem mass spectrometry; MCCS, The Melbourne Collaborative Cohort Study; MR, mendelian randomisation; NSHDS, Northern Sweden Health and Disease study; SNP, single nucleotide polymorphism.

### Study population, sample collection, and follow-up

Our study population consisted of kidney cancer nested case–control studies drawn from 5 independent cohorts: the European Prospective Investigation into Cancer and Nutrition (EPIC), The Melbourne Collaborative Cohort Study (MCCS), Northern Sweden Health and Disease study (NSHDS), University of Tartu—Estonian Biobank (Estonian BB), and The Trøndelag Health Study (HUNT) (Table A in S3 Table; details of the cohorts are described in the S1 Methods). Cases were defined as participants diagnosed with incident malignant neoplasm of the kidney or renal pelvis (International Classification of Diseases for Oncology, 3rd Edition [ICD-O-3] code C64/C65) who gave a blood sample at recruitment. In each independent cohort, one randomly selected control without history of kidney cancer was matched to each case based on age, sex, and date of blood collection. There were small variations between the cohorts in the tightness by which controls were matched to cases according to their age and date of blood draw (see S1 Methods), owing to inherent differences in demography and availability of controls. The study was approved by the International Agency for Research on Cancer (IARC) Ethics Committee.

### Metabolite data acquisition and quality control

Plasma and serum samples from 2,614 participants (1,307 cases and 1,307 controls) were analysed. Samples from all cohorts were analysed using the Biocrates targeted MS assay. Samples from EPIC and NSHDS (*n* = 1,596) were additionally analysed using Metabolon’s untargeted MS platform. Samples from matched case–control pairs were assayed in adjacent wells (in random order) and in the same analytical batch. Laboratory personnel were blinded to case–control status of the samples.

An overview of the quality control (QC) pipeline is shown in S1 Fig. All the QC steps were performed for each cohort separately before pooling the data.

#### Targeted metabolomics—Biocrates

All samples from EPIC and MCCS were assayed at the IARC, while samples from NSHDS, HUNT, and the Estonian BB were assayed by the Metabolomics Core Facility of the Genome Analysis Center of the Helmholtz Zentrum München [32]. The targeted metabolomics approach was based on LC-ESI-MS/MS and FIA-ESI-MS/MS measurements using the Absolute*IDQ* p180 Kit (BIOCRATES Life Sciences, Innsbruck, Austria). The assay allows simultaneous quantification of 188 metabolites using 10-μL plasma or serum. Sample preparation and MS measurements were performed as described in S1 Methods. The median intra- and inter-batch coefficients of variation (CV) were 5.6% and 6.9%, respectively (interquartile range = 1.7% and 2.8%, respectively). The lower limits of detection (LODs) were set to 3 times the values of the zero samples (PBS solution).

Values lower than the lower limit of quantification (LLOQ) or higher than the upper limit of quantification (ULOQ), as well as lower than batch-specific LOD (for compounds semiquantified: acylcarnitines, glycerophospholipids (GPLs), and sphingolipids), were imputed with half of the LOD/LLOQ or the ULOQ. For NSHDS, metabolites with internal standard out of range were left as missing (*n* = 205). Metabolites with less than 100 values above LOD/LLOQ in any individual cohort were excluded from the analyses. In our samples, a total of 164 metabolites were retained for statistical analyses (30 acylcarnitines, 21 amino acids, 10 biogenic amines, 88 GPLs, 14 sphingolipids, and the sum of hexoses). In addition to individual metabolites, 22 ratios or sums selected for their capacity to provide detailed insight into a wide range of disorders of the metabolic disease spectrum were computed (listed in Table B in S3 Table). Among them, the Fischer ratio, a clinical indicator of liver metabolism and function, was calculated as the molar ratio of branched chain amino acids (leucine + isoleucine + valine) to aromatic amino acids (phenylalanine + tyrosine). Lower Fischer ratio values are associated with liver dysfunction.

#### Untargeted metabolomics—Metabolon

Untargeted metabolomic analyses were performed at Metabolon (Durham, North Carolina, United States of America) on a platform consisting of 4 independent ultra-high performance liquid chromatography—tandem mass spectrometry (UPLC–MS/MS) methods. Detailed descriptions of the platform and workflow to identify features, including extraction of raw data, peak identification, and internal quality control (QC) processes can be found in the S1 Methods and in published work [33–35]. Samples from EPIC and NSHDS were processed as 2 independent experimental batches. The median intra-batch CV were 5% and 4% for EPIC and NSHDS, respectively, while the median inter-batch CV were 11% for both EPIC and NSHDS. A variety of curation procedures were carried out by Metabolon to ensure that a high-quality data set was made available for statistical analysis and data interpretation (S1 Methods). Each metabolite was rescaled to set the median equal to 1 and missing values imputed with the minimum observed value. Data returned for EPIC comprised a total of 1,308 metabolite features, 982 of known identity (named biochemicals) and 326 compounds of unknown structural identity (unnamed biochemicals). Data returned for NSHDS comprised a total of 1,302 metabolite features, 979 of known identity (named biochemicals) and 323 compounds of unknown structural identity (unnamed biochemicals). A total of 1,275 metabolites were available across the 2 data sets with the total number of unique metabolites reaching 1,335. Metabolites were categorised by Metabolon as belonging to 1 of 8 mutually exclusive chemical classes: amino acids and amino acid derivatives (subsequently referred to as “amino acids”), carbohydrates, cofactors and vitamins, energy metabolites, lipids, nucleotides, peptides, or xenobiotics. An asterisk (*) at the end of the metabolite name indicates the metabolite identity has not been confirmed by comparison with an authentic chemical standard. After the exclusion of metabolites for which less than 100 participants had values recorded (86 and 176 for EPIC and NSHDS, respectively), 1,230 metabolite features remained for analysis (1,222 and 1,126 for EPIC and NSHDS, respectively; 1,118 in common).

### Statistical analysis

#### Primary statistical analysis: Prospective observational analysis of circulating metabolites and kidney cancer risk

Log-transformed and standardised (z-score) metabolite concentrations were used in all analyses. Crude conditional logistic regressions were performed to estimate the odds ratio (OR) for kidney cancer per 1 standard deviation (SD) increment in log-transformed metabolite concentrations, conditioning on the individual case–control sets. To consider multiple comparisons while accounting for the correlation between the different metabolites, we estimated the effective number of independent tests (ENT) performed as the number of principal components explaining more than 95% of the variance in our metabolite matrices. Metabolites with *p*-values equal or below 0.05/ENT in the pooled analyses and equal or below 0.05 in at least 2 cohorts independently were deemed robustly associated with kidney cancer risk. For these metabolites, we carried out additional conditional logistic regressions adjusted for BMI, smoking history (smoking status: never, former, current smokers, and pack years of smoking), lifetime alcohol consumption (in g/day), and hypertension (ever/never). To avoid comparing different sets of participants due to missingness in risk factor data, we restricted these analyses to study participants with complete risk factor information.

To further characterise the epidemiological properties of the association between metabolites and kidney cancer risk, we also carried out conditional logistic regression stratified by age at blood collection, sex, country, BMI, waist-to-hip ratio, smoking status, alcohol consumption, hypertension, and time to diagnosis (number of years between blood draw and diagnosis).

#### Secondary statistical analysis: mendelian randomisation and profile comparison analyses

We initially investigated pleiotropy among potential SNP instruments for the circulating metabolites associated with kidney cancer risk in prospective analyses (Biocrates and Metabolon) with a view to conducting a 2-sample MR analysis for metabolites (as the exposure) and kidney cancer risk (as the outcome). SNP–metabolite associations were extracted from the largest genome-wide association studies (GWASs) currently available for circulating metabolites and included summary statistics for 174 Biocrates metabolites [36] (*N* = ranged from 8,569 to 56,040 for different metabolites, depending on the platform used in each contributing study) and 913 Metabolon metabolites (*N* = 14,296). Specifically, pleiotropy was assessed by estimating the variance explained in all metabolites by the single nucleotide polymorphisms (SNPs) (i.e., the potential “instruments”) associated with each of our candidate risk metabolites (see S1 Methods for more details of how instruments were selected). Where the variance explained in other metabolites (i.e., those not associated with risk in the prospective analysis) was similar to that explained in the candidate risk metabolite, we inferred low metabolite specificity for current GWAS results, and thus violation of the MR assumptions necessary to infer potential single exposure causality.

To evaluate the extent to which the metabolomic signature of disease risk could be explained by BMI, we first conducted a 2-sample MR analysis to provide estimates of the causal relationships between BMI and circulating metabolites (Biocrates and Metabolon). A total of 549 independent SNPs (R^2^ < 0.01) that were robustly associated with BMI at genome-wide significance were selected as instruments from the largest GWAS meta-analysis for BMI from the Genetic Investigation of Anthropometric Traits (GIANT) consortium (*n* = approximately 700,000 [37]; see Table C in S3 Table). SNP–exposure associations were extracted from the BMI GWAS meta-analysis [37], and SNP–outcome associations were extracted from the metabolite GWAS described above. A BMI effect estimate was generated for each metabolite measured and calculated as an SD unit increase in log-transformed metabolite level per SD increment in BMI. The primary MR analysis was conducted using the inverse-variance weighted (IVW) method [38]. We performed the following sensitivity analyses to attempt to account for potential unbalanced horizontal pleiotropy: (1) MR–Egger regression to test overall directional pleiotropy and provide a valid causal estimate, taking into account the presence of pleiotropy [39]; and (2) weighted median [40], which provides a consistent estimate of causal effect if at least 50% of the information in the analysis comes from variants that are valid instrumental variables. To account for multiple testing, we used the same *p*-value threshold as used in our observational analyses (*p* < 8.3 × 10^−4^ and *p* < 1 × 10^−4^ for Biocrates and Metabolon, respectively).

To examine the extent to which kidney cancer–associated metabolites are driven by BMI, we assessed the correlation between the kidney cancer–associated metabolite profile (metabolites associated with kidney cancer risk in the prospective observational analyses) and the BMI-associated metabolite profile (metabolites associated with BMI levels in the MR analyses) using Spearman rank correlation analyses. Effect estimates from both the prospective and MR analyses were divided by the standard error of the estimate before conducting the correlation analyses.

#### Negative control analyses

The presence or absence of overlap between metabolite profiles flagged by prospective analysis and those derived from BMI MR is only informative in the context of a null or negative control comparator. To allow this, we repeated the profile comparison analysis described above (with BMI as the exposure) in an analysis in which we used dental disease as a negative control exposure (i.e., an exposure not likely to be a risk factor for kidney cancer) and one that we would therefore expect to deliver a null. This strategy of repeating an experiment under conditions that are expected to deliver a null result has previously been advocated within observational epidemiology [41]. In our analysis of the causal relationship between dental disease and circulating metabolites, 47 independent (R^2^ < 0.01) SNPs that were robustly associated at genome-wide significance (*p* < 5 × 10^−8^) were selected from the largest GWAS for dental disease (*n* = 487,823) (detailed information for instrumental variables for dental disease are presented in Table D in S3 Table). SNP–exposure associations were extracted from the largest dental disease GWAS meta-analysis [42], and SNP–outcome associations were extracted from the metabolite GWAS described above. Effect estimates were calculated as SD unit increase in metabolite levels per logOR increase in dental disease. Methods used in the 2-sample MR analyses were as described above.

All MR analyses were performed using the TwoSample MR R package version 0.4.13 (http://github.com/MRCIEU/TwoSampleMR) [43].

## Results

### Population characteristics and metabolites overview

Demographic and baseline characteristics for the 1,305 cases and 1,305 matched controls are presented in Table 1. The mean age at diagnosis for cases was 65.6 years (SD = 9.79), and cases were diagnosed on average 8 years after blood collection. The majority (58%) of samples were collected after fewer than 6 hours of fasting. Overall, 186 metabolites or ratios/sums of metabolites were measured using the Biocrates assay on 2,610 samples (all cohorts), and 1,230 metabolites were measured using the Metabolon platform on 1,596 samples (EPIC and NSHDS cohorts). Mean concentrations of the 1,416 metabolites by case–control status are shown in Table E in S3 Table.

**Table 1:** Population characteristics of the 2,610 kidney cancer cases and controls from 5 independent cohorts with pre-diagnostic blood samples included in our analyses.

	Cases	Controls
	Mean (SD) or *N* (%)	Mean (SD) or *N* (%)
**Total**	1,305	1,305
**Age at blood collection (years)**	57.6 (10.1)	57.6 (10.1)
**Length of follow-up from blood collection (years)**	7.95 (4.98)	-
**Histology**		
Clear cell	931 (71.3)	-
Other	282 (21.6)	-
Unknown	92 (7.1)	-
**Sex**		
Male	725 (55.6)	725 (55.6)
Female	580 (44.4)	580 (44.4)
**Cohort**		
EPIC	634 (48.6)	634 (48.6)
Estonian BB	115 (8.8)	115 (8.8)
HUNT	254 (19.5)	254 (19.5)
MCCS	139 (10.6)	139 (10.6)
NSHDS	163 (12.5)	163 (12.5)
**Education**		
None	43 (3.3)	52 (4)
Primary school	468 (35.9)	456 (34.9)
Technical school	233 (17.9)	222 (17)
Secondary school	239 (18.3)	236 (18.1)
University	216 (16.6)	242 (18.5)
Unknown	106 (8.1)	97 (7.4)
**BMI**		
Mean (SD)	27.79 (4.62)	26.95 (4.28)
**BMI classes**		
<18.5	6 (0.5)	6 (0.5)
[18.5 to 25]	364 (27.9)	458 (35.1)
[25 to 30]	596 (45.7)	581 (44.5)
> = 30	335 (25.7)	254 (19.5)
Unknown	4 (0.3)	6 (0.5)
**Smoking status**		
Never	553 (42.4)	603 (46.2)
Former	418 (32)	445 (34.1)
Current	315 (24.1)	233 (17.9)
Unknown	19 (1.5)	24 (1.8)
**Smoking quantity**		
Pack years; mean (SD)	11.77 (17.13)	9.63 (15.34)
Min–max	0.00 to 153.45	0.00 to 100.00
**Alcohol consumption (g/d)**		
Mean (SD)	13.85 (25.14)	14.87 (29.61)
**Diabetes**		
No	1,069 (81.9)	1,099 (84.2)
Yes	80 (6.1)	54 (4.1)
Unknown	156 (12)	152 (11.7)
**Hypertension**		
No	612 (46.9)	718 (55)
Yes	433 (33.2)	333 (25.5)
Unknown	260 (19.9)	254 (19.5)
**Fasting status**		
Fasting for less than 6 hours	768 (58.8)	759 (58.2)
Fasting for 6 hours or more	476 (36.5)	497 (38.1)
Unknown	61 (4.7)	49 (3.7)

BMI, body mass index; d, days; EPIC, The European Prospective Investigation into Cancer and Nutrition; Estonian BB, University of Tartu—Estonian Biobank; g, grams; HUNT, The Trøndelag Health Study; MCCS, The Melbourne Collaborative Cohort Study; *N*, number of participants; NSHDS, Northern Sweden Health and Disease study; OR, odds ratio; SD, standard deviation.

### Prospective observational analysis of circulating metabolites and kidney cancer risk

**Fig 2:**
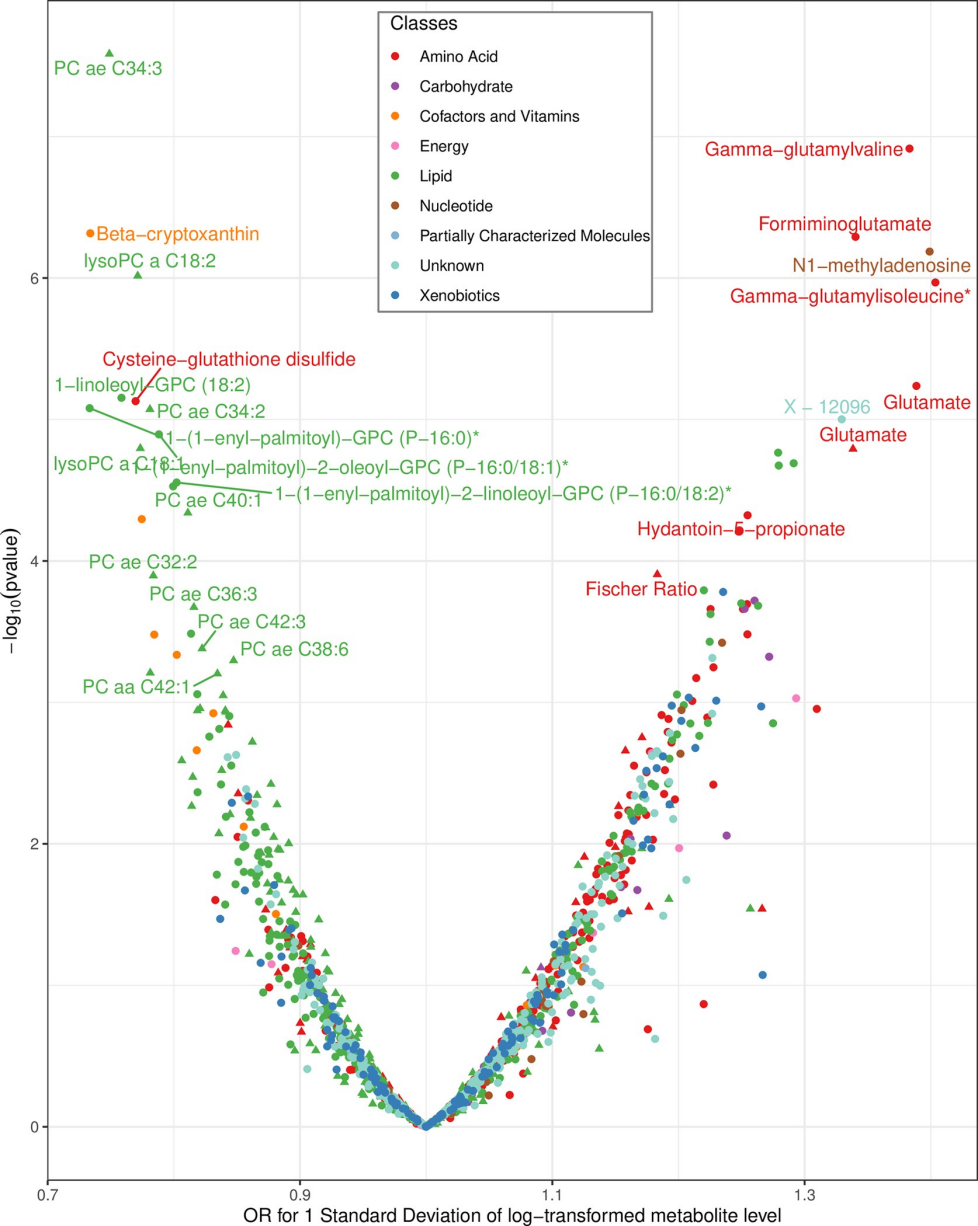
**Volcano plot depicting the association between circulating metabolites measured by either Biocrates (triangles) or Metabolon (dots) with kidney cancer risk in 5 prospective cohorts.** Metabolites that are labelled have a *p*-value below the threshold (*p* < 0.05/ENTs) in the pooled analyses and are nominally significant in at least 2 cohorts separately. * Metabolite identity not yet confirmed by comparison with an authentic chemical standard. ORs and CIs were estimated for 1 SD of log-transformed metabolite levels by logistic regression conditioned on case set. Estimated ENT are 60 and 499 for Biocrates and Metabolon metabolites, respectively. *p*-Values threshold are thus 8.33E-04 and 1.00E-04 for Biocrates and Metabolon metabolites, respectively. CI, confidence interval; ENT, effective number of test; OR, odds ratio; SD, standard deviation.

We identified 25 metabolites robustly associated with kidney cancer risk (i.e., metabolites associated with risk after correction for multiple testing in the pooled analysis and nominally significant in at least 2 cohorts; Fig 2, Table 2). Among these metabolites, 12 were measured with the Biocrates assay, and 13 were measured with the Metabolon platform. Two metabolites—glutamate and 1-linoleoyl-GPC (18:2) (known as lysoPC a C18:2 in Biocrates)—were measured on both platforms and resulted in similar risk association estimates (for glutamate OR: 1.34 in Biocrates and 1.39 in Metabolon; for 1-linoleoyl-GPC (18:2), OR: 0.77 in Biocrates and 0.76 in Metabolon). Pearson correlations among risk metabolites are displayed in S2 Fig.

**Table 2:** Metabolites robustly^Ψ^ associated with kidney cancer risk.

		Crude^a^				Adjusted for BMI^b^			
Metabolite name	Class	N_pairs_	OR	95% CI	*p*-Value	N_pairs_	OR	95% CI	*p*-Value
***Biocrates***									
Glutamate	Amino acid	1,300	1.34	1.17 to 1.53	1.62E-05	1,290	1.24	1.08 to 1.42	2.46E-03
Fischer ratio	Amino acid (ratio)	1,300	1.18	1.09 to 1.29	1.25E-04	1,290	1.14	1.04 to 1.24	5.02E-03
PC ae C34:3	GPLs	1,304	0.75	0.68 to 0.83	2.61E-08	1,294	0.79	0.71 to 0.88	1.05E-05
lysoPC a C18:2	GPLs	1,304	0.77	0.70 to 0.86	9.65E-07	1,294	0.81	0.73 to 0.90	1.35E-04
PC ae C34:2	GPLs	1,304	0.78	0.70 to 0.87	8.47E-06	1,294	0.82	0.73 to 0.91	4.00E-04
lysoPC a C18:1	GPLs	1,304	0.77	0.69 to 0.87	1.60E-05	1,294	0.81	0.72 to 0.92	8.04E-04
PC ae C40:1	GPLs	1,304	0.81	0.73 to 0.90	4.57E-05	1,294	0.84	0.76 to 0.93	8.96E-04
PC ae C32:2	GPLs	1,304	0.78	0.69 to 0.89	1.27E-04	1,294	0.81	0.72 to 0.92	1.31E-03
PC ae C36:3	GPLs	1,304	0.82	0.73 to 0.91	2.12E-04	1,294	0.85	0.76 to 0.95	3.24E-03
PC ae C42:3	GPLs	1,304	0.82	0.74 to 0.92	4.17E-04	1,294	0.87	0.78 to 0.98	1.75E-02
PC ae C38:6	GPLs	1,304	0.85	0.77 to 0.93	5.06E-04	1,294	0.86	0.78 to 0.95	1.85E-03
PC aa C42:1	GPLs	1,304	0.83	0.75 to 0.93	6.27E-04	1,294	0.88	0.79 to 0.99	2.59E-02
***Metabolon***									
Formiminoglutamate	Amino acid	798	1.34	1.20 to 1.50	5.11E-07	794	1.28	1.14 to 1.45	4.23E-05
Glutamate	Amino acid	798	1.39	1.20 to 1.60	5.79E-06	794	1.30	1.11 to 1.51	8.02E-04
Cysteine-glutathione disulphide	Amino acid	798	0.77	0.69 to 0.86	7.42E-06	794	0.79	0.70 to 0.89	6.99E-05
Hydantoin-5-propionate	Amino acid	798	1.25	1.12 to 1.39	6.17E-05	794	1.22	1.09 to 1.36	3.76E-04
Beta-cryptoxanthin	Cofactors and vitamins	798	0.73	0.65 to 0.83	4.83E-07	794	0.76	0.67 to 0.86	1.81E-05
1-linoleoyl-GPC (18:2)	GPLs	798	0.76	0.67 to 0.86	7.03E-06	794	0.79	0.70 to 0.89	2.04E-04
1-(1-enyl-palmitoyl)-GPC (P-16:0)*	GPLs	798	0.73	0.64 to 0.84	8.32E-06	794	0.77	0.67 to 0.88	1.71E-04
1-(1-enyl-palmitoyl)-2-oleoyl-GPC (P-16:0/18:1)*	GPLs	798	0.79	0.71 to 0.88	1.27E-05	794	0.83	0.74 to 0.93	1.41E-03
1-(1-enyl-palmitoyl)-2-linoleoyl-GPC (P-16:0/18:2)*	GPLs	798	0.80	0.72 to 0.89	2.79E-05	794	0.84	0.76 to 0.94	1.61E-03
N1-methyladenosine	Nucleotide	798	1.40	1.23 to 1.60	6.50E-07	794	1.35	1.18 to 1.55	8.74E-06
Gamma-glutamylvaline	Peptide	798	1.38	1.23 to 1.56	1.22E-07	794	1.32	1.17 to 1.49	1.24E-05
Gamma-glutamylisoleucine*	Peptide	798	1.40	1.22 to 1.61	1.07E-06	794	1.33	1.15 to 1.53	1.01E-04
X– 12096	Unknown	798	1.33	1.17 to 1.51	9.97E-06	794	1.27	1.12 to 1.45	2.40E-04

* Metabolite identity not yet confirmed by comparison with an authentic chemical standard.

^a^ ORs and CIs were estimated for 1 SD of log-transformed metabolite levels by logistic regression conditioned on case set.

^b^ ORs and CIs were estimated for 1 SD of log-transformed metabolite levels by logistic regression conditioned on case set and adjusted for BMI

^Ψ^ *p*-Values below 0.05/ENT in the pooled analyses and at least nominally significant in 2 cohorts independently.

Estimated ENT are 60 and 499 for Biocrates and Metabolon metabolites, respectively. *p*-Values threshold are thus 8.33E-04 and 1.00E-04 for Biocrates and Metabolon metabolites, respectively.

BMI, body mass index; CI, confidence interval; ENT, effective number of test; GPL, glycerophospholipid; N_pairs_, number of case control pairs included in the analyses; OR, odds ratio.

We found that increased concentrations of 14 individual GPLs were associated with reduced kidney cancer risk. These included 8 phosphatidylcholines (PCs; overall *p*-values ranging from 6 × 10^−4^ to 3 × 10^−8^), among which PC ae C34:3 had the strongest association (OR = 0.75, 95% confidence interval [CI]: 0.68 to 0.83, *p* = 2.61 × 10^−8^). Similar associations were identified for the lysophosphatidyl-cholines, lysoPC a C18:1, and lysoPC a C18:2 (labelled as 1-linoleoyl-GPC (18:2) in Metabolon) (*p*-values between 1.60 × 10^−5^ and 9.65 × 10^−7^). Two plasmalogens were also inversely associated with risk, 1-(1-enyl-palmitoyl-2-oleoyl-GPC (P-16:0/18:1) (*p* = 1.27 × 10^−5^) and 1-(1-enyl-palmitoyl)-2-linoleoyl-GPC (P-16:0/18:2) (*p* = 2.79 × 10^−5^), as well as the lysoplasmalogen 1-(1-enyl-palmitoyl)-GPC (P-16:0) (*p* = 8.32 × 10^−6^).

Among 274 metabolites involved in amino acid metabolism, we found 4 positively associated with kidney cancer risk, including glutamate, formiminoglutamate, hydantoin-5-propionate and the Fischer ratio (*p*-values between 1.25 × 10^−4^ and 5.11 × 10^−7^). For example, the relative odds of kidney cancer associated with an SD increment in log-transformed glutamate levels was estimated at 1.39 (95% CI: 1.20 to 1.60) when measured on the Metabolon platform. Another amino acid, cysteine-glutathione disulphide was inversely associated with risk (OR: 0.77, 95% CI: 0.69 to 0.86, *p* = 7.42 × 10^−6^). The 2 peptides gamma-glutamylvaline (*p* = 1.22 × 10^−7^) and gamma-glutamylisoleucine (*p* = 1.07 × 10^−6^) were positively associated with risk. Finally, we found beta-cryptoxanthin negatively associated with kidney cancer risk (OR: 0.73, 95% CI: 0.65, 0.83, *p* = 4.83 × 10^−7^), while an unidentified metabolite (X-12096) was positively associated (OR: 1.33, 95% CI: 1.17, 1.51, *p* = 9.97 × 10^−6^). Adjusting for the fasting status of the samples (more versus less than 6 hours) did not modify the OR estimates for the identified risk metabolites (Table F in S3 Table).

Associations with risk of kidney cancer for all metabolites analysed are presented in Table G in S3 Table.

### The influence of kidney cancer risk factors on kidney cancer–associated metabolites

We assessed the extent to which known modifiable risk factors could explain the observed associations by multivariable analyses. For all 25 metabolites found to be associated with risk in the primary analysis, we found that adjustments for BMI partly attenuated the OR estimates for some metabolites, although they all remained at least nominally significant (i.e., *p*-value below 0.05, Table 2). The association most modified by adjustment for BMI was that of glutamate (from 1.34, 95% CI: 1.17 to 1.53, *p* = 1.62 × 10^−5^ to 1.24, 95% CI: 1.08 to 1.42, *p* = 2.46 × 10^−3^), followed by PC ae C42:3 and PC aa C42:1 (OR increased by 6% for both metabolites: from 0.82, 95% CI: 0.74 to 0.92, *p* = 4.17 × 10^−4^ to 0.87, 95% CI: 0.78 to 0.98, *p* = 1.75 × 10^−2^ and 0.83, 95% CI: 0.75 to 0.93, *p* = 6.27 × 10^−4^ to 0.88, 95% CI: 0.79 to 0.99, *p* = 2.59 × 10^−2^ for PC ae C42:3 and PC aa C42:1, respectively). Conversely, association for PC ae C38:6 was not influenced by adjustment for BMI (OR:0.85, 95% CI: 0.77 to 0.93, *p* = 5.06 × 10^−4^ to 0.86, 95% CI: 00.78 to 0.95, *p* = 1.85 × 10^−3^). Results adjusted for all individual risk factors on participants with complete information on these risk factors are shown in Table H in S3 Table (*N* = 1,162 and 996 for Biocrates and Metabolon, respectively). Adjustment for smoking and alcohol consumption did not modify any OR by more than 1.5% and 1.2%, respectively, whereas adjusting for hypertension partly attenuated the associations of lysoPC a C18:1 and lysoPC a C18:2, albeit to a lesser extent than BMI (5% change for both). In fully adjusted models, risk associations remained nominally significant (*p*-value below 0.05) for 10 out of 25 metabolites with all effect estimates in the same direction as in the primary analysis, although, due to missing data for some risk factors, this analysis included only 581 and 498 case–control pairs for Biocrates and Metabolon, respectively.

In stratified risk analyses by time to diagnosis (Figs A–Y in S3 Fig), several metabolites appeared to display a stronger risk association closer to diagnosis, including 1-(1-enyl-palmitoyl)-2-linoleoyl-GPC (P-16:0/18:2) (heterogeneity *p* = 0.02) (Fig M in S3 Fig) and the metabolite of unknown structural identity X-12096 (heterogeneity *p* = 0.02) that was measured on the Metabolon platform (Fig Y in S3 Fig). The lysophosphatidyl-choline lysoPC a C18:2, as measured by Biocrates, showed a stronger association when alcohol consumption was above the median compared to lower (heterogeneity *p* = 0.03) (Fig D in S3 Fig); this pattern was evident for the same metabolite measured in Metabolon but was not statistically significant (heterogeneity *p* = 0.3) (Fig P in S3 Fig).

### Two-sample mendelian randomisation and profile comparison analyses

We identified genetic instruments for 17 of the 25 risk metabolites but observed substantial pleiotropy for the instruments defined for 16 of the 17 instrumented metabolites. The total variance explained from a risk metabolite’s instruments was typically similar across classes of metabolite (lipids and 1-(1-enyl-palmitoyl)-2-linoleoyl-GPC (P-16:0/18:2), for example) and far from specific to the given risk metabolite being instrumented. Further, the variance explained was often higher for an alternative metabolite compared to the risk metabolite (see Figs A–Q in S4 Fig). Following these observations, we chose not to carry out a formal MR analysis of the relation between individual metabolites and kidney cancer risk because the profound pleiotropy across metabolites clearly violates the MR assumptions.

Rather, to complement the risk analyses, and to gain further understanding of how BMI—the leading modifiable risk factor of kidney cancer—might explain our findings, we conducted a 2-sample MR analysis to evaluate the extent to which the measured metabolites are driven by differences in BMI. Using the IVW method, 60 metabolites (22 Biocrates and 38 Metabolon) were associated with BMI. In an MR framework, there was consistent evidence between both platforms that BMI was associated with decreased concentrations of many GPLs and increased concentrations of several amino acids and nucleotides, as well as acylcarnitines, sphingomyelins, and several metabolites of unknown identity (S5 Fig). Estimates from MR–Egger and weighted median analyses were consistent with the IVW estimates (Tables I and J in S3 Table).

**Fig 3:**
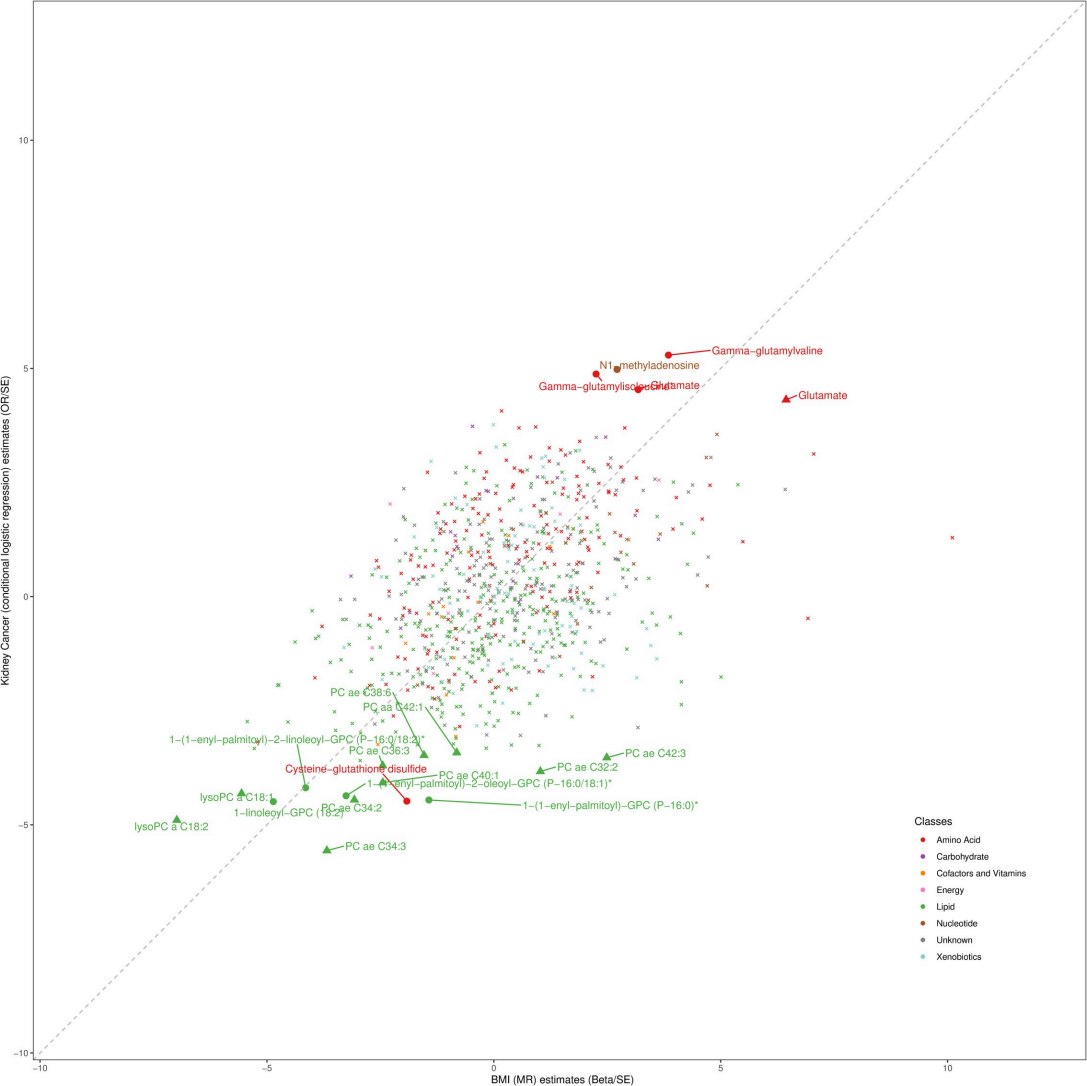
Scatter plot comparing the metabolite profile associated with kidney cancer from prospective observational analyses with the BMI-driven metabolite profile from MR analyses. Metabolites that are labelled have a *p*-value below the threshold (*p* < 0.05/ENTs) in the prospective pooled analyses and are nominally significant in at least 2 cohorts separately. Metabolites measured by the Biocrates platform that are below the *p*-value threshold are represented by triangles, those measured by the Metabolon platform that are below the *p*-value threshold are represented by dots, and those that are measured by either the Biocrates or the Metabolon platform that are above the *p*-value threshold are represented by an x. * Metabolite identity not yet confirmed by comparison with an authentic chemical standard. On the y-axis, the OR and SE were derived from the logistic regression analyses conditioned on case set estimating the associations between circulating metabolites and kidney cancer risk in 5 prospective cohorts. On the x-axis, the beta and SE were derived from the MR analyses evaluating the effect of BMI on circulating metabolites levels. BMI, body mass index; ENT, effective number of test; MR, mendelian randomisation; OR, odds ratio; SE, standard error.

When comparing the metabolic profile of kidney cancer (metabolites associated with kidney cancer risk in the prospective analyses) and BMI (metabolites associated with BMI levels in the MR analyses), we observed moderate correlation between the BMI-driven metabolite profile and metabolite profile associated with kidney cancer risk (Fig 3) (r = 0.53, *p* = 2.2 × 10^−6^ for Biocrates metabolites and r = 0.36, *p* = 2.2 × 10^−6^ for Metabolon metabolites). Specifically, elevated BMI appeared to decrease levels of several GPLs that were also found inversely associated with kidney cancer risk, including 1-(1-enyl-palmitoyl)-2-linoleoyl-GPC (P-16:0/18:2)*, 1-linoleoyl-GPC (18:2) (lysoPC a C18:2), lysoPC a C18:1, and PC ae C34:3. For instance, 1 SD increment in BMI was associated with a 0.17 SD decrease in 1-(1-enyl-palmitoyl)-2-linoleoyl-GPC (P-16:0/18:2) levels ([ß_BMI_], *p* = 3.4 × 10^−5^). We also found that BMI was associated with increased levels of glutamate (ß_BMI_: 0.12, *p* = 1.5 × 10^−3^), which was positively associated with kidney cancer risk. Several metabolites associated with kidney cancer risk in our prospective analysis did not appear to be strongly influenced by BMI, but we note that for all but 2 metabolites (PC ae 32:2 and PC ae 42:3), estimates were directionally concordant (i.e., positively correlated) but with the effect size estimates from the BMI MR being closer to the null than those seen in the observational analysis. Conversely, some of the metabolites that were most strongly affected by BMI (e.g., phenylalanine and valine) were not associated with kidney cancer risk.

### Negative control analyses

There was little evidence that genetic predisposition to dental disease influenced circulating metabolite levels with no metabolites reaching our predetermined threshold for a statistically significant association (Tables K and L in S3 Table). We observed low correlation between the dental disease metabolite estimates from MR analyses and the kidney cancer metabolite estimates from the prospective analysis for both Biocrates (r = 0.15, *p* = 0.06) and Metabolon metabolites (r = 0.12, *p* = 0.002) (S5 Fig). None of the 25 metabolites that were associated with kidney cancer risk in prospective analyses were associated with dental disease from the MR analyses (S5 Fig). These findings suggest that when the profile comparison analysis is conducted using a hypothetically unrelated exposure (dental disease), we see no meaningful relationship between metabolite associations from the prospective analysis and the MR.

## Discussion

This study describes the relationship between the pre-diagnostic blood-metabolome and risk of developing kidney cancer based on data from 5 longitudinal population cohorts. This is the first comprehensive metabolomics analysis of incident kidney cancer to be conducted using a prospective design, and as such, complements existing work characterising the metabolic profile (in tissue and biofluids) of the disease itself [16–26]. We investigated 1,416 metabolites in relation to the occurrence of kidney cancer using 2 complementary analytical methods and observed 25 metabolites to be robustly associated with risk. These metabolites included 14 GPLs inversely associated with risk, 5 amino acids positively associated, and 1 inversely associated with risk, as well as risk associations for a carotenoid, 2 peptides, a nucleotide, and an unidentified feature. Results of an MR analysis designed to evaluate the extent to which BMI influences the key risk-associated metabolites suggest that differences in BMI may be responsible for part of the metabolite profile associated with the development of kidney cancer.

The majority of metabolites found to be associated with kidney cancer risk in this study can be classified as GPLs. GPLs are the main component of cell membranes and are essential for maintaining cellular structure and for regulating cell signalling. The circulating metabolite associations we see here pre-diagnosis appear to intersect with the known cellular metabolic programming observed within kidney tumour tissue. For example, it has been proposed that clear cell RCC cells use exogenous lipids for membrane formation and cell signalling [44]. The relationship between lipid metabolites and prospective kidney cancer risk reported in our study could, theoretically, be capturing increased uptake of lipid metabolites by preclinical kidney carcinogenesis.

GPLs can be broadly classified into 2 types based on their biochemical structure—diacyl (aa) or acyl-alkyl (ae)—and can be further characterised according to their lipid side chain composition, specifically the number of carbons and their degree of (un)saturation (number of double bonds). The association of a subset of long chain unsaturated (mainly acyl-alkyl) PCs, lysophophatidylcholines (LPCs), and plasmalogens with reduced kidney cancer risk is consistent with some limited existing literature. Specifically, lower levels of total PC/choline have been reported in the serum of diagnosed kidney cancer patients compared to control participants [17], and numerous studies have found decreased LPCs in both tumour and normal kidney tissues [27,45,46], as well as in the circulation of kidney cancer patients [18,47]. The mechanisms underpinning these associations are not well understood, but some of these molecules (e.g., plasmalogens) have been proposed as antioxidants [48]. Low levels of plasmalogens in cancer patients have been proposed as a potential mechanism by which increased oxidative stress could drive cancer progression [49].

We assessed the extent to which known risk factors could explain the observed metabolite associations and observed that adjusting for BMI—the main modifiable risk factor for kidney cancer—partially attenuated (less than 9% change in OR) the risk association for some specific metabolites. To further understand the relation with BMI for the kidney cancer risk-associated metabolites, we estimated the causal influence of BMI on metabolite levels using MR. This analysis clearly demonstrated that some—but not all—metabolites inversely associated with kidney cancer risk are also decreased by elevated BMI (e.g., several GPLs), whereas other metabolites positively associated with risk (e.g., glutamate) are also increased by elevated BMI. The association of long chain unsaturated (mainly acyl-alkyl) GPLs with both lower risk of RCC and lower BMI is consistent with extensive literature linking lower levels of these and similar molecules to a range of common diseases that include a metabolic component such as obesity and hypertension [50–52], type 2 diabetes [53], type 1 diabetes development [54], and nonalcoholic fatty liver disease [55].

Glutamate was found to be positively associated with both kidney cancer risk and BMI and was also the metabolite for which adjusting for BMI resulted in the greatest attenuation in its OR estimate. Glutamate and glutamine are both found to be increased in kidney tumour tissue [44]. This observation provides further evidence of overlap between metabolites relevant to disease development and those whose levels are perturbed in the disease state [10,56]. Consistent with our findings, glutamate has previously been shown to be increased in visceral obesity [57,58], and glutamine-derived glutamate has been linked to tumour cell metabolism [59], with RCC being no exception [60]. α-Ketoglutarate, generated from glutamine-derived glutamate, enters the tricarboxylic acid (TCA) cycle providing both energy and biosynthetic intermediates [61]. A large intracellular glutamate pool is also important for nonessential amino acid synthesis in addition to cellular redox regulation [61]. Two previous NMR-based studies found lower levels of glutamine in serum of kidney cancer cases taken at diagnosis compared to controls [16,17]. While we did not identify a robust association of glutamine in our study, the point estimate was consistent with a weak inverse association with risk of kidney cancer.

A final overarching observation was that in comparison with previously published prospective metabolomics analyses on other cancer sites [62–64], the sheer number of metabolites found to be associated with risk in the current study suggests that the blood metabolome is particularly important in the aetiology of kidney cancer.

### Strengths, limitations, and prospects for future studies

The chief strength of our study was the design of the primary risk analysis wherein control participants were individually matched to incident kidney cancer cases with pre-diagnostic blood samples from 5 independent population cohorts, a design that minimised differential bias and allowed for identification of novel and robust risk metabolites of kidney cancer. The use of 2 complementary metabolomics platforms also increased the overall coverage of the metabolome. The well-characterised cohorts offered the opportunity to carefully assess the influence of known kidney cancer risk factors (i.e., potential confounders) on identified risk-associated metabolites, as well as the robustness of their risk associations across the independent cohort studies. Well-designed prospective studies can provide compelling evidence in favour of a role of molecular risk factors in cancer aetiology, but residual confounding from imperfectly measured risk factors may still bias the association estimates. We therefore complemented the main risk analysis with a genetic analysis to assess the influence of BMI on the identified risk metabolites. We believe that this independent analysis provided important independent evidence when interpreting the relation between the identified risk metabolites and kidney cancer risk in the context of BMI—the principal risk factor of kidney cancer.

Limitations of our study include the presence of measurement error in the (semi-) quantification of metabolites. However, by using well-established platforms with built-in validation procedures along with randomisation schemes to ensure any batch variation was orthogonal to the outcome of interest (in this case kidney cancer case status), we can be confident there was no systematic bias in our estimates as a result of measurement error. In addition, the consistency in estimates we see for metabolites that appear on both platforms provides increased confidence in our results, but we note that statistical power to identify risk metabolites exclusive to the Metabolon platform was lower than for metabolites exclusive to the Biocrates platform due to the lower sample size. In this study, we focused on those metabolites that demonstrated consistency in risk associations across the 5 participating cohorts. While this approach ensured the robustness of the estimates, any risk marker present in specific populations would not be highlighted. Although we only measured metabolite levels at a single time point, we do not believe this represents a major limitation as the majority of measured metabolites have a high within person stability over time (stable over 4 months to 2 years) [65–67]. Another limitation of our study is the lack of detailed data on body composition. It is possible that some individual risk markers may reflect a certain adiposity distribution that is specifically strongly associated with kidney cancer risk. While the current literature on kidney cancer aetiology does not highlight any specific aspect of obesity as being particularly important in kidney cancer aetiology, evaluating the identified risk markers in relation to detailed body composition (e.g., using DEXA scan data) represents an appealing future focus of our kidney cancer research. The remaining limitations relate to the generalisability of our findings. Given evidence for specific metabolic alterations by kidney cancer histotype [10], it is possible that kidney cancer subtypes have different dependencies on circulating metabolites. In this case, findings from this study are likely most relevant to the major histological subtype—clear cell RCC—which made up 71% of kidney cancer cases. Furthermore, our study does not inform on the extent to which the identified risk markers translate to populations of non-European descent. Addressing these limitations should constitute an important focus for future studies addressing the role of the blood metabolome in the aetiology of kidney cancer.

While the results of our prospective risk analysis are consistent with circulating metabolites playing an important role in kidney cancer aetiology, it is appealing to complement such observational analyses with MR studies to further inform causal inference. However, we chose not to carry out an MR analysis on kidney cancer risk for individual metabolites for a number of reasons related to characteristics specific to circulating metabolites. Firstly, owing to high correlational structure of many metabolites, few SNPs have been found associated with specific metabolites, leading to pleiotropic instruments for most metabolites [36]. Secondly, there is a high degree of pleiotropy for metabolite-associated SNPs with modifiable risk factors and other disease endpoints. That few metabolites have a sufficient number of instruments is particularly problematic as applying statistical methods aiming to correct for these biases is not possible (e.g., MR–Egger and MR-PRESSO), nor is the use of techniques designed to evaluate the effect of multiple correlated exposures (e.g., multivariable MR [68]). While the genetic architecture of blood metabolites is complicated for the reasons outlined above, there are hundreds of independent SNPs robustly associated with BMI [37], and this gave us greater confidence in the application of this analysis [69]. Better characterising of the genetic architecture of circulating metabolites together with methodological advancements may allow for more robust causal inference in future metabolomics studies.

## Conclusions

This study points to a particularly important role of the blood metabolome in kidney cancer aetiology, specifically by identifying positive risk associations for several amino acids, as well as negative risk associations with multiple lipids, including PCs, LPCs, and plasmalogens. Downstream analyses indicated that some—but not all—risk metabolites are influenced by BMI, which partly explains their associations with kidney cancer risk, whereas the risk associations for other metabolites could not be explained by known risk factors. These results provide important insight into the metabolic pathways underpinning the central role of obesity in kidney cancer aetiology and clues to novel pathways involved in kidney cancer aetiology.

## Supporting information

S1 Methods Supporting information methods. (DOCX)

S1 Table STROBE Statement—Checklist of items that should be included in reports of case–control studies. STROBE, Strengthening the Reporting of Observational Studies in Epidemiology.
(DOCX)

S2 Table STROBE-MR checklist. MR, mendelian randomisation; STROBE, Strengthening the Reporting of Observational Studies in Epidemiology.
(DOCX)

S3 Table **Table A:** Population characteristics of the kidney cancer cases and controls from 5 independent cohorts with pre-diagnostic blood samples included in our analyses, by cohort. **Table B:** Description of the predefined sums and ratios of metabolites measured with the Biocrates assay as described in the manufacturer’s documentation. **Table C:** Genetic instruments associated with BMI used in 2-sample MR analyses. **Table D:** Genetic instruments associated with dental disease used in 2-sample MR analyses. **Table E:** Mean concentration of metabolites by case or control status (in μmol/L and raw area counts for Biocrates and Metablon, respectively). **Table F:** Associations between levels of circulating metabolites and kidney cancer risk adjusted for fasting status. **Table G:** Crude associations between levels of circulating metabolites and kidney cancer risk. **Table H:** Associations between levels of circulating metabolites and kidney cancer risk adjusted for risk factors (for metabolites robustly associated with kidney cancer risk in the crude analyses and restricted to participants with complete risk factor information). **Table I:** Association between BMI and circulating Biocrates metabolites from 2-sample MR analyses. **Table J:** Association between BMI and circulating Metabolon metabolites from 2-sample MR analyses. **Table K:** Association between dental disease and circulating Biocrates metabolites from 2-sample MR analyses. **Table L:** Association between dental disease and circulating Metabolon metabolites from 2-sample MR analyses. BMI, body mass index; MR, mendelian randomisation.
(XLSX)

S1 Fig Overview of the QC pipelines used for the metabolite measurements pre-processing. QC, quality control.
(DOCX)

S2 Fig Heatmap of Pearson correlation coefficients for selected Biocrates (left) and Metabolon (right) metabolites. (DOCX)

S3 Fig Forest plots depicting the kidney cancer risk association for each metabolite deemed robustly associated with kidney cancer risk, stratified by specific kidney cancer risk factors. **Fig A:** Forest plots depicts the kidney cancer risk association for the Fischer ratio stratified by risk factors. **Fig B:** Forest plots depicts the kidney cancer risk association for glutamate (Biocrates), stratified by risk factors. **Fig C:** Forest plots depicts the kidney cancer risk association for lysoPC a C18:1, stratified by risk factors. **Fig D:** Forest plots depicts the kidney cancer risk association for lysoPC a C18:2, stratified by risk factors. **Fig E:** Forest plots depicts the kidney cancer risk association for PC aa C42:1, stratified by risk factors. **Fig F:** Forest plots depicts the kidney cancer risk association for PC ae C32:2, stratified by risk factors. **Fig G:** Forest plots depicts the kidney cancer risk association for PC ae C34:2, stratified by risk factors. **Fig H:** Forest plots depicts the kidney cancer risk association for PC ae C34:3, stratified by risk factors. **Fig I:** Forest plots depicts the kidney cancer risk association for PC ae C36:3, stratified by risk factors. **Fig J:** Forest plots depicts the kidney cancer risk association for PC ae C38:6, stratified by risk factors. **Fig K:** Forest plots depicts the kidney cancer risk association for PC ae C40:1, stratified by risk factors. **Fig L:** Forest plots depicts the kidney cancer risk association for PC ae C42:3, stratified by risk factors. **Fig M:** Forest plots depicts the kidney cancer risk association for 1-(1-enyl-palmitoyl)-2-linoleoyl-GPC (P-16:0/18:2)*, stratified by risk factors. **Fig N:** Forest plots depicts the kidney cancer risk association for 1-(1-enyl-palmitoyl)-2-oleoyl-GPC (P-16:0/18:1)*, stratified by risk factors. **Fig O:** Forest plots depicts the kidney cancer risk association for 1-(1-enyl-palmitoyl)-GPC (P-16:0)*, stratified by risk factors. **Fig P:** Forest plots depicts the kidney cancer risk association for 1-linoleoyl-GPC (18:2), stratified by risk factors. **Fig Q:** Forest plots depicts the kidney cancer risk association for beta-cryptoxanthin, stratified by risk factors. **Fig R:** Forest plots depicts the kidney cancer risk association for cysteine-glutathione disulphide, stratified by risk factors. **Fig S:** Forest plots depicts the kidney cancer risk association for formiminoglutamate, stratified by risk factors. **Fig T:** Forest plots depicts the kidney cancer risk association for gamma-glutamylisoleucine*, stratified by risk factors. **Fig U:** Forest plots depicts the kidney cancer risk association for gamma-glutamylvaline, stratified by risk factors. **Fig V:** Forest plots depicts the kidney cancer risk association for glutamate (Metabolon), stratified by risk factors. **Fig W:** Forest plots depicts the kidney cancer risk association for hydantoin-5-propionate, stratified by risk factors. **Fig X:** Forest plots depicts the kidney cancer risk association for N1-methyladenosine, stratified by risk factors. **Fig Y:** Forest plots depicts the kidney cancer risk association for X-12096, stratified by risk factors. GPC, Glycerophosphocholine; PC, phosphatidylcholine.
(DOCX)

S4 Fig **Scatter plots of the cumulative variance explained in the Metabolon/Biocrates metabolites by the genome-wide significant (*p* < 5 × 10**^**−**^**8) independent (R**^**2**^ **< 0.01) SNPs for the specified risk metabolite (labelled in red). Fig A:** Scatter plots of the cumulative variance explained by the genome-wide significant (*p* < 5 × 10^−^8) independent (R^2^ < 0.01) SNPs for cysteine-glutathione disulphide (Metabolon). **Fig B:** Scatter plots of the cumulative variance explained by the genome-wide significant (*p* < 5 × 10^−^8) independent (R^2^ < 0.01) SNPs for Hydantoin-5-propionate (Metabolon). **Fig C:** Scatter plots of the cumulative variance explained by the genome-wide significant (*p* < 5 × 10^−^8) independent (R^2^ < 0.01) SNPs for 1-linoleoyl-GPC (18:2) (Metabolon). **Fig D:** Scatter plots of the cumulative variance explained by the genome-wide significant (*p* < 5 × 10^−^8) independent (R^2^ < 0.01) SNPs for 1-(1-enyl-palmitoyl)-GPC (P-16:0) (Metabolon). **Fig E:** Scatter plots of the cumulative variance explained by the genome-wide significant (*p* < 5 × 10^−^8) independent (R^2^ < 0.01) SNPs for 1-(1-enyl-palmitoyl)-2-oleoyl-GPC (P-16:0/18:1) (Metabolon). **Fig F:** Scatter plots of the cumulative variance explained by the genome-wide significant (*p* < 5 × 10^−^8) independent (R^2^ < 0.01) SNPs for 1-(1-enyl-palmitoyl)-2-linoleoyl-GPC (P-16:0/18:2) (Metabolon). **Fig G:** Scatter plots of the cumulative variance explained by the genome-wide significant (*p* < 5 × 10^−^8) independent (R^2^ < 0.01) SNPs for N1-methyladenosine (Metabolon). **Fig H:** Scatter plots of the cumulative variance explained by the genome-wide significant (*p* < 5 × 10^−^8) independent (R^2^ < 0.01) SNPs for PC ae C34:3 (Biocrates). **Fig I:** Scatter plots of the cumulative variance explained by the genome-wide significant (*p* < 5 × 10^−^8) independent (R^2^ < 0.01) SNPs for lysoPC a C18:2 (Biocrates). **Fig J:** Scatter plots of the cumulative variance explained by the genome-wide significant (*p* < 5 × 10^−^8) independent (R^2^ < 0.01) SNPs for PC ae C34:2 (Biocrates). **Fig K:** Scatter plots of the cumulative variance explained by the genome-wide significant (*p* < 5 × 10^−^8) independent (R^2^ < 0.01) SNPs for lysoPC a C18:1 (Biocrates). **Fig L:** Scatter plots of the cumulative variance explained by the genome-wide significant (*p* < 5 × 10^−^8) independent (R^2^ < 0.01) SNPs for PC ae C40:1 (Biocrates). **Fig M:** Scatter plots of the cumulative variance explained by the genome-wide significant (*p* < 5 × 10^−^8) independent (R^2^ < 0.01) SNPs for PC ae C32:2 (Biocrates). **Fig N:** Scatter plots of the cumulative variance explained by the genome-wide significant (*p* < 5 × 10^−^8) independent (R^2^ < 0.01) SNPs for PC ae C36:3 (Biocrates). **Fig O:** Scatter plots of the cumulative variance explained by the genome-wide significant (*p* < 5 × 10^−^8) independent (R^2^ < 0.01) SNPs for PC ae C42:3 (Biocrates). **Fig P:** Scatter plots of the cumulative variance explained by the genome-wide significant (*p* < 5 × 10^−^8) independent (R^2^ < 0.01) SNPs for PC ae C38:6 (Biocrates). **Fig Q:** Scatter plots of the cumulative variance explained by the genome-wide significant (*p* < 5 × 10^−^8) independent (R^2^ < 0.01) SNPs for PC aa C42:1 (Biocrates). GPC, Glycerophosphocholine; PC, phosphatidylcholine; SNP, single nucleotide polymorphism.
(DOCX)

S5 Fig Volcano plots representing the association between BMI and circulating Biocrates metabolites (triangle) and Metabolon metabolites (dots) from MR analyses. BMI, body mass index; MR, mendelian randomisation.
(DOCX)

S6 Fig Scatter plots comparing the metabolite profile associated with kidney cancer from prospective observational analyses with the dental disease–driven metabolite profile from MR analyses. MR, mendelian randomisation.
(DOCX)

## Abbreviations

BMI: body mass index
CI: confidence interval
CV: coefficients of variation
ENT: effective number of independent tests
EPIC: The European Prospective Investigation into Cancer and Nutrition
Estonian BB: University of Tartu—Estonian Biobank
GIANT: Genetic Investigation of Anthropometric Traits
GPC: Glycerophosphocholine
GPL: glycerophospholipid
GWAS: genome-wide association study
HUNT: The Trøndelag Health Study
IARC: International Agency for Research on Cancer
ICD-O-3: International Classification of Diseases for Oncology: 3rd Edition
IVW: inverse-variance weighted
LLOQ: lower limit of quantification
LOD: limit of detection
LPC: lysophosphatidylcholine
MCCS: The Melbourne Collaborative Cohort Study
MR: mendelian randomisation
MS: mass spectrometry
NMR: nuclear magnetic resonance
NSHDS: Northern Sweden Health and Disease study
OR: odds ratio
PC: phosphatidylcholine
QC: quality control
RCC: renal cell carcinoma
SD: standard deviation
STROBE: Strengthening the Reporting of Observational Studies in Epidemiology
TCA: tricarboxylic acid
ULOQ: upper limit of quantification
UPLC–MS/MS: ultra-high performance liquid chromatography—tandem mass spectrometry

## References

[1] Ferlay J EM, Lam F, Colombet M, Mery L, Piñeros M, Znaor A, et al. Global Cancer Observatory: Cancer Today. 2018 [cited 13 Nov 2020]. Available from: https://gco.iarc.fr/today.

[2] Scelo G, Larose TL. Epidemiology and Risk Factors for Kidney Cancer. J Clin Oncol. 2018:JCO2018791905. Epub 2018/10/30. doi: 10.1200/JCO.2018.79.1905 . 30372394 PMC6299342

[3] Haggstrom C, Rapp K, Stocks T, Manjer J, Bjorge T, Ulmer H, et al. Metabolic factors associated with risk of renal cell carcinoma. PLoS ONE. 2013;8(2):e57475. doi: 10.1371/journal.pone.0057475 . 23468995 PMC3585341

[4] Stocks T, Bjorge T, Ulmer H, Manjer J, Haggstrom C, Nagel G, et al. Metabolic risk score and cancer risk: pooled analysis of seven cohorts. Int J Epidemiol. 2015;44(4):1353–63. Epub 2015/02/06. doi: 10.1093/ije/dyv001 . 25652574 PMC4588859

[5] Laaksonen MA, MacInnis RJ, Canfell K, Giles GG, Hull P, Shaw JE, et al. The future burden of kidney and bladder cancers preventable by behavior modification in Australia: A pooled cohort study. Int J Cancer. 2020;146(3):874–83. Epub 2019/05/21. doi: 10.1002/ijc.32420 . 31107541

[6] Johansson M, Carreras-Torres R, Scelo G, Purdue MP, Mariosa D, Muller DC, et al. The influence of obesity-related factors in the etiology of renal cell carcinoma-A mendelian randomization study. PLoS Med. 2019;16(1):e1002724. Epub 2019/01/04. doi: 10.1371/journal.pmed.1002724 . 30605491 PMC6317776

[7] Wang F, Xu Y. Body mass index and risk of renal cell cancer: a dose-response meta-analysis of published cohort studies. Int J Cancer. 2014;135(7):1673–86. Epub 2014/03/13. doi: 10.1002/ijc.28813 . 24615287

[8] Brown KF, Rumgay H, Dunlop C, Ryan M, Quartly F, Cox A, et al. The fraction of cancer attributable to modifiable risk factors in England, Wales, Scotland, Northern Ireland, and the United Kingdom in 2015. Br J Cancer. 2018;118(8):1130–41. Epub 2018/03/24. doi: 10.1038/s41416-018-0029-6 . 29567982 PMC5931106

[9] Linehan WM, Srinivasan R, Schmidt LS. The genetic basis of kidney cancer: a metabolic disease. Nat Rev Urol. 2010;7(5):277–85. Epub 2010/05/08. doi: 10.1038/nrurol.2010.47 . 20448661 PMC2929006

[10] DiNatale RG, Sanchez A, Hakimi AA, Reznik E. Metabolomics informs common patterns of molecular dysfunction across histologies of renal cell carcinoma. Urol Oncol. 2020;38(10):755–62. Epub 2019/06/04. doi: 10.1016/j.urolonc.2019.04.028 . 31155438

[11] Civelek M, Lusis AJ. Systems genetics approaches to understand complex traits. Nat Rev Genet. 2014;15(1):34–48. Epub 2013/12/04. doi: 10.1038/nrg3575 . 24296534 PMC3934510

[12] Homuth G, Teumer A, Volker U, Nauck M. A description of large-scale metabolomics studies: increasing value by combining metabolomics with genome-wide SNP genotyping and transcriptional profiling. J Endocrinol. 2012;215(1):17–28. Epub 2012/07/12. doi: 10.1530/JOE-12-0144 . 22782382

[13] Johnson CH, Ivanisevic J, Siuzdak G. Metabolomics: beyond biomarkers and towards mechanisms. Nat Rev Mol Cell Biol. 2016;17(7):451–9. Epub 2016/03/17. doi: 10.1038/nrm.2016.25 . 26979502 PMC5729912

[14] Sullivan LB, Gui DY, Vander Heiden MG. Altered metabolite levels in cancer: implications for tumour biology and cancer therapy. Nat Rev Cancer. 2016;16(11):680–93. Epub 2016/10/25. doi: 10.1038/nrc.2016.85 . 27658530

[15] Wishart DS. Emerging applications of metabolomics in drug discovery and precision medicine. Nat Rev Drug Discov. 2016;15(7):473–84. Epub 2016/03/12. doi: 10.1038/nrd.2016.32 . 26965202

[16] Zira AN, Theocharis SE, Mitropoulos D, Migdalis V, Mikros E. (1)H NMR metabonomic analysis in renal cell carcinoma: a possible diagnostic tool. J Proteome Res. 2010;9(8):4038–44. Epub 2010/06/10. doi: 10.1021/pr100226m . 20527959

[17] Gao H, Dong B, Liu X, Xuan H, Huang Y, Lin D. Metabonomic profiling of renal cell carcinoma: high-resolution proton nuclear magnetic resonance spectroscopy of human serum with multivariate data analysis. Anal Chim Acta. 2008;624(2):269–77. Epub 2008/08/19. doi: 10.1016/j.aca.2008.06.051 . 18706333

[18] Lin L, Huang Z, Gao Y, Yan X, Xing J, Hang W. LC-MS based serum metabonomic analysis for renal cell carcinoma diagnosis, staging, and biomarker discovery. J Proteome Res. 2011;10(3):1396–405. Epub 2010/12/29. doi: 10.1021/pr101161u . 21186845

[19] Lin L, Yu Q, Yan X, Hang W, Zheng J, Xing J, et al. Direct infusion mass spectrometry or liquid chromatography mass spectrometry for human metabonomics? A serum metabonomic study of kidney cancer. Analyst. 2010;135(11):2970–8. Epub 2010/09/22. doi: 10.1039/c0an00265h . 20856980

[20] Ganti S, Taylor SL, Kim K, Hoppel CL, Guo L, Yang J, et al. Urinary acylcarnitines are altered in human kidney cancer. Int J Cancer. 2012;130(12):2791–800. Epub 2011/07/07. doi: 10.1002/ijc.26274 . 21732340 PMC3258465

[21] Ganti S, Weiss RH. Urine metabolomics for kidney cancer detection and biomarker discovery. Urol Oncol. 2011;29(5):551–7. Epub 2011/09/21. doi: 10.1016/j.urolonc.2011.05.013 . 21930086 PMC3177099

[22] Kind T, Tolstikov V, Fiehn O, Weiss RH. A comprehensive urinary metabolomic approach for identifying kidney cancerr. Anal Biochem. 2007;363(2):185–95. Epub 2007/02/24. doi: 10.1016/j.ab.2007.01.028 . 17316536

[23] Perroud B, Lee J, Valkova N, Dhirapong A, Lin PY, Fiehn O, et al. Pathway analysis of kidney cancer using proteomics and metabolic profiling. Mol Cancer. 2006;5:64. Epub 2006/11/25. doi: 10.1186/1476-4598-5-64 . 17123452 PMC1665458

[24] Niziol J, Bonifay V, Ossolinski K, Ossolinski T, Ossolinska A, Sunner J, et al. Metabolomic study of human tissue and urine in clear cell renal carcinoma by LC-HRMS and PLS-DA. Anal Bioanal Chem. 2018;410(16):3859–69. Epub 2018/04/17. doi: 10.1007/s00216-018-1059-x . 29658093 PMC5956006

[25] Wettersten HI, Hakimi AA, Morin D, Bianchi C, Johnstone ME, Donohoe DR, et al. Grade-Dependent Metabolic Reprogramming in Kidney Cancer Revealed by Combined Proteomics and Metabolomics Analysis. Cancer Res. 2015;75(12):2541–52. doi: 10.1158/0008-5472.CAN-14-1703 . 25952651 PMC4470795

[26] Saito K, Arai E, Maekawa K, Ishikawa M, Fujimoto H, Taguchi R, et al. Lipidomic Signatures and Associated Transcriptomic Profiles of Clear Cell Renal Cell Carcinoma. Sci Rep. 2016;6:28932. Epub 2016/07/01. doi: 10.1038/srep28932 . 27357243 PMC4928052

[27] Schaeffeler E, Buttner F, Reustle A, Klumpp V, Winter S, Rausch S, et al. Metabolic and Lipidomic Reprogramming in Renal Cell Carcinoma Subtypes Reflects Regions of Tumor Origin. Eur Urol Focus. 2019;5(4):608–18. Epub 2018/02/18. doi: 10.1016/j.euf.2018.01.016 . 29452772

[28] Yet I, Menni C, Shin SY, Mangino M, Soranzo N, Adamski J, et al. Genetic Influences on Metabolite Levels: A Comparison across Metabolomic Platforms. PLoS ONE. 2016;11(4):e0153672. Epub 2016/04/14. doi: 10.1371/journal.pone.0153672 . 27073872 PMC4830611

[29] Smith GD, Ebrahim S. ’Mendelian randomization’: can genetic epidemiology contribute to understanding environmental determinants of disease? Int J Epidemiol. 2003;32(1):1–22. Epub 2003/04/12. doi: 10.1093/ije/dyg070 . 12689998

[30] von Elm E, Altman DG, Egger M, Pocock SJ, Gotzsche PC, Vandenbroucke JP, et al. The Strengthening the Reporting of Observational Studies in Epidemiology (STROBE) statement: guidelines for reporting observational studies. PLoS Med. 2007;4(10):e296. Epub 2007/10/19. doi: 10.1371/journal.pmed.0040296 . 17941714 PMC2020495

[31] Davey Smith G, Davies NM, Dimou N, Egger M, Gallo V, Golub R, et al. STROBE-MR: Guidelines for strengthening the reporting of Mendelian randomization studies. PeerJ. 2019;7:e27857v1. 10.7287/peerj.preprints.27857v1.

[32] Zukunft S, Sorgenfrei M, Prehn C, Moller G, Adamski J. Targeted Metabolomics of Dried Blood Spot Extracts. Chromatographia. 2013;76(19–20):1295–305. doi: 10.1007/s10337-013-2429-3 WOS:000324825100011.

[33] Evans AM, DeHaven CD, Barrett T, Mitchell M, Milgram E. Integrated, nontargeted ultrahigh performance liquid chromatography/electrospray ionization tandem mass spectrometry platform for the identification and relative quantification of the small-molecule complement of biological systems. Anal Chem. 2009;81(16):6656–67. Epub 2009/07/25. doi: 10.1021/ac901536h . 19624122

[34] Dehaven CD, Evans AM, Dai H, Lawton KA. Organization of GC/MS and LC/MS metabolomics data into chemical libraries. J Cheminform. 2010;2(1):9. Epub 2010/10/20. doi: 10.1186/1758-2946-2-9 . 20955607 PMC2984397

[35] Zierer J, Jackson MA, Kastenmuller G, Mangino M, Long T, Telenti A, et al. The fecal metabolome as a functional readout of the gut microbiome. Nat Genet. 2018;50(6):790–5. Epub 2018/05/29. doi: 10.1038/s41588-018-0135-7 . 29808030 PMC6104805

[36] Lotta LA, Pietzner M, Stewart ID, Wittemans LBL, Li C, Bonelli R, et al. A cross-platform approach identifies genetic regulators of human metabolism and health. Nat Genet. 2021;53(1):54–64. Epub 2021/01/09. doi: 10.1038/s41588-020-00751-5 . 33414548 PMC7612925

[37] Yengo L, Sidorenko J, Kemper KE, Zheng Z, Wood AR, Weedon MN, et al. Meta-analysis of genome-wide association studies for height and body mass index in approximately 700000 individuals of European ancestry. Hum Mol Genet. 2018;27(20):3641–9. Epub 2018/08/21. doi: 10.1093/hmg/ddy271 . 30124842 PMC6488973

[38] Burgess S, Butterworth A, Thompson SG. Mendelian randomization analysis with multiple genetic variants using summarized data. Genet Epidemiol. 2013;37(7):658–65. Epub 2013/10/12. doi: 10.1002/gepi.21758 . 24114802 PMC4377079

[39] Bowden J, Davey Smith G, Burgess S. Mendelian randomization with invalid instruments: effect estimation and bias detection through Egger regression. Int J Epidemiol. 2015;44(2):512–25. Epub 2015/06/08. doi: 10.1093/ije/dyv080 . 26050253 PMC4469799

[40] Bowden J, Davey Smith G, Haycock PC, Burgess S. Consistent Estimation in Mendelian Randomization with Some Invalid Instruments Using a Weighted Median Estimator. Genet Epidemiol. 2016;40(4):304–14. Epub 2016/04/12. doi: 10.1002/gepi.21965 . 27061298 PMC4849733

[41] Lipsitch M, Tchetgen Tchetgen E, Cohen T. Negative controls: a tool for detecting confounding and bias in observational studies. Epidemiology. 2010;21(3):383–8. Epub 2010/03/26. doi: 10.1097/EDE.0b013e3181d61eeb . 20335814 PMC3053408

[42] Shungin D, Haworth S, Divaris K, Agler CS, Kamatani Y, Keun Lee M, et al. Genome-wide analysis of dental caries and periodontitis combining clinical and self-reported data. Nat Commun. 2019;10(1):2773. Epub 2019/06/27. doi: 10.1038/s41467-019-10630-1 . 31235808 PMC6591304

[43] Hemani G, Zheng J, Elsworth B, Wade KH, Haberland V, Baird D, et al. The MR-Base platform supports systematic causal inference across the human phenome. Elife. 2018;7. Epub 2018/05/31. doi: 10.7554/eLife.34408 . 29846171 PMC5976434

[44] Lucarelli G, Loizzo D, Franzin R, Battaglia S, Ferro M, Cantiello F, et al. Metabolomic insights into pathophysiological mechanisms and biomarker discovery in clear cell renal cell carcinoma. Expert Rev Mol Diagn. 2019;19(5):397–407. Epub 2019/04/16. doi: 10.1080/14737159.2019.1607729 . 30983433

[45] Cifkova E, Holcapek M, Lisa M, Vrana D, Gatek J, Melichar B. Determination of lipidomic differences between human breast cancer and surrounding normal tissues using HILIC-HPLC/ESI-MS and multivariate data analysis. Anal Bioanal Chem. 2015;407(3):991–1002. Epub 2014/10/30. doi: 10.1007/s00216-014-8272-z . 25352274

[46] Du Y, Wang Q, Zhang X, Wang X, Qin C, Sheng Z, et al. Lysophosphatidylcholine acyltransferase 1 upregulation and concomitant phospholipid alterations in clear cell renal cell carcinoma. J Exp Clin Cancer Res. 2017;36(1):66. Epub 2017/05/13. doi: 10.1186/s13046-017-0525-1 . 28494778 PMC5427523

[47] Sullentrop F, Moka D, Neubauer S, Haupt G, Engelmann U, Hahn J, et al. 31P NMR spectroscopy of blood plasma: determination and quantification of phospholipid classes in patients with renal cell carcinoma. NMR Biomed. 2002;15(1):60–8. Epub 2002/02/13. doi: 10.1002/nbm.758 . 11840554

[48] Engelmann B. Plasmalogens: targets for oxidants and major lipophilic antioxidants. Biochem Soc Trans. 2004;32(Pt 1):147–50. Epub 2004/01/30. doi: 10.1042/bst0320147 . 14748736

[49] Messias MCF, Mecatti GC, Priolli DG, de Oliveira Carvalho P. Plasmalogen lipids: functional mechanism and their involvement in gastrointestinal cancer. Lipids Health Dis. 2018;17(1):41. Epub 2018/03/09. doi: 10.1186/s12944-018-0685-9 . 29514688 PMC5842581

[50] Graessler J, Schwudke D, Schwarz PE, Herzog R, Shevchenko A, Bornstein SR. Top-down lipidomics reveals ether lipid deficiency in blood plasma of hypertensive patients. PLoS ONE. 2009;4(7):e6261. Epub 2009/07/16. doi: 10.1371/journal.pone.0006261 . 19603071 PMC2705678

[51] Pietilainen KH, Sysi-Aho M, Rissanen A, Seppanen-Laakso T, Yki-Jarvinen H, Kaprio J, et al. Acquired obesity is associated with changes in the serum lipidomic profile independent of genetic effects—a monozygotic twin study. PLoS ONE. 2007;2(2):e218. Epub 2007/02/15. doi: 10.1371/journal.pone.0000218 . 17299598 PMC1789242

[52] Wahl S, Yu Z, Kleber M, Singmann P, Holzapfel C, He Y, et al. Childhood obesity is associated with changes in the serum metabolite profile. Obes Facts. 2012;5(5):660–70. Epub 2012/10/31. doi: 10.1159/000343204 . 23108202

[53] Razquin C, Toledo E, Clish CB, Ruiz-Canela M, Dennis C, Corella D, et al. Plasma Lipidomic Profiling and Risk of Type 2 Diabetes in the PREDIMED Trial. Diabetes Care. 2018;41(12):2617–24. Epub 2018/10/18. doi: 10.2337/dc18-0840 . 30327364 PMC6245212

[54] Oresic M, Simell S, Sysi-Aho M, Nanto-Salonen K, Seppanen-Laakso T, Parikka V, et al. Dysregulation of lipid and amino acid metabolism precedes islet autoimmunity in children who later progress to type 1 diabetes. J Exp Med. 2008;205(13):2975–84. Epub 2008/12/17. doi: 10.1084/jem.20081800 . 19075291 PMC2605239

[55] Oresic M, Hyotylainen T, Kotronen A, Gopalacharyulu P, Nygren H, Arola J, et al. Prediction of non-alcoholic fatty-liver disease and liver fat content by serum molecular lipids. Diabetologia. 2013;56(10):2266–74. Epub 2013/07/05. doi: 10.1007/s00125-013-2981-2 . 23824212 PMC3764317

[56] Lucarelli G, Rutigliano M, Sallustio F, Ribatti D, Giglio A, Lepore Signorile M, et al. Integrated multi-omics characterization reveals a distinctive metabolic signature and the role of NDUFA4L2 in promoting angiogenesis, chemoresistance, and mitochondrial dysfunction in clear cell renal cell carcinoma. Aging (Albany NY). 2018;10(12):3957–85. Epub 2018/12/13. doi: 10.18632/aging.101685 . 30538212 PMC6326659

[57] Takashina C, Tsujino I, Watanabe T, Sakaue S, Ikeda D, Yamada A, et al. Associations among the plasma amino acid profile, obesity, and glucose metabolism in Japanese adults with normal glucose tolerance. Nutr Metab (Lond). 2016;13:5. Epub 2016/01/21. doi: 10.1186/s12986-015-0059-5 . 26788116 PMC4717594

[58] Boulet MM, Chevrier G, Grenier-Larouche T, Pelletier M, Nadeau M, Scarpa J, et al. Alterations of plasma metabolite profiles related to adipose tissue distribution and cardiometabolic risk. Am J Physiol Endocrinol Metab. 2015;309(8):E736–46. Epub 2015/08/27. doi: 10.1152/ajpendo.00231.2015 . 26306599

[59] Li T, Le A. Glutamine Metabolism in Cancer. Adv Exp Med Biol. 2018;1063:13–32. doi: 10.1007/978-3-319-77736-8_2 WOS:000451318000003. 29946773

[60] Shroff EH, Eberlin LS, Dang VM, Gouw AM, Gabay M, Adam SJ, et al. MYC oncogene overexpression drives renal cell carcinoma in a mouse model through glutamine metabolism. Proc Natl Acad Sci U S A. 2015;112(21):6539–44. Epub 2015/05/13. doi: 10.1073/pnas.1507228112 . 25964345 PMC4450371

[61] DeBerardinis RJ, Chandel NS. Fundamentals of cancer metabolism. Sci Adv. 2016;2(5):e1600200. Epub 2016/07/08. doi: 10.1126/sciadv.1600200 . 27386546 PMC4928883

[62] His M, Viallon V, Dossus L, Gicquiau A, Achaintre D, Scalbert A, et al. Prospective analysis of circulating metabolites and breast cancer in EPIC. BMC Med. 2019;17(1):178. Epub 2019/09/25. doi: 10.1186/s12916-019-1408-4 . 31547832 PMC6757362

[63] Shu X, Xiang YB, Rothman N, Yu D, Li HL, Yang G, et al. Prospective study of blood metabolites associated with colorectal cancer risk. Int J Cancer. 2018;143(3):527–34. Epub 2018/02/27. doi: 10.1002/ijc.31341 . 29479691 PMC6019169

[64] Shu X, Zheng W, Yu D, Li HL, Lan Q, Yang G, et al. Prospective metabolomics study identifies potential novel blood metabolites associated with pancreatic cancer risk. Int J Cancer. 2018;143(9):2161–7. Epub 2018/05/03. doi: 10.1002/ijc.31574 . 29717485 PMC6195470

[65] Carayol M, Licaj I, Achaintre D, Sacerdote C, Vineis P, Key TJ, et al. Reliability of Serum Metabolites over a Two-Year Period: A Targeted Metabolomic Approach in Fasting and Non-Fasting Samples from EPIC. PLoS ONE. 2015;10(8):e0135437. Epub 2015/08/15. doi: 10.1371/journal.pone.0135437 . 26274920 PMC4537119

[66] Floegel A, Drogan D, Wang-Sattler R, Prehn C, Illig T, Adamski J, et al. Reliability of serum metabolite concentrations over a 4-month period using a targeted metabolomic approach. PLoS ONE. 2011;6(6):e21103. Epub 2011/06/24. doi: 10.1371/journal.pone.0021103 . 21698256 PMC3115978

[67] Townsend MK, Clish CB, Kraft P, Wu C, Souza AL, Deik AA, et al. Reproducibility of metabolomic profiles among men and women in 2 large cohort studies. Clin Chem. 2013;59(11):1657–67. Epub 2013/07/31. doi: 10.1373/clinchem.2012.199133 . 23897902 PMC3812240

[68] Sanderson E, Davey Smith G, Windmeijer F, Bowden J. An examination of multivariable Mendelian randomization in the single-sample and two-sample summary data settings. Int J Epidemiol. 2018. Epub 2018/12/12. doi: 10.1093/ije/dyy262 . 30535378 PMC6734942

[69] Burgess S, Davies NM, Thompson SG. Bias due to participant overlap in two-sample Mendelian randomization. Genet Epidemiol. 2016;40(7):597–608. Epub 2016/10/19. doi: 10.1002/gepi.21998 . 27625185 PMC5082560

