# Supplementary material for: The blood metabolome of incident kidney cancer: A case–control study nested within the MetKid consortium
Source: PLoS Med. 2021 Sep 20;18(9):e1003786. doi: 10.1371/journal.pmed.1003786 (PMC8496779; doi:10.1371/journal.pmed.1003786)
Supplement: S1 Methods — (DOCX) [file pmed.1003786.s001.docx]

**Supplementary Methods**

Table of Contents

[Study population 2](#_Toc82023377)

[European Prospective Investigation into Cancer and Nutrition (EPIC) 2](#_Toc82023378)

[Northern Sweden Health and Disease study (NSHDS) 3](#_Toc82023379)

[The Trøndelag Health Study (HUNT) 4](#_Toc82023380)

[The Melbourne Collaborative Cohort Study (MCCS) 4](#_Toc82023381)

[University of Tartu - Estonian Biobank (Estonian BB) 5](#_Toc82023382)

[Metabolite data acquisition 6](#_Toc82023383)

[Biocrates 6](#_Toc82023384)

[Metabolon 7](#_Toc82023385)

[Data sources for Mendelian randomization analyses 8](#_Toc82023386)

[BMI GWAS 8](#_Toc82023387)

[Metabolite GWAS 8](#_Toc82023388)

[Dental disease GWAS 11](#_Toc82023389)

[References 12](#_Toc82023390)

# Study population

| **Acronym** | **Country/Region** | **Cohort name** | **N case-control pairs** | **Biocrates metabolites** | **Metabolon metabolites** |
| --- | --- | --- | --- | --- | --- |
| **EPIC** | EUROPE | European Prospective Investigation into Cancer and Prevention | 635 | x | x |
| **NSHDS** | SWEDEN | Northern Sweden Health and Disease Study | 163 | x | x |
| **HUNT** | NORWAY | The Nord-Trøndelag Health Study | 254 | x | - |
| **MCCS** | AUSTRALIA | The Melbourne Collaborative Cohort Study | 140 | x | - |
| **Estonian BB** | ESTONIA | Estonian Genome Center - Estonian Biobank | 115 | x | - |

*Overview of the cohorts included in the study population*

## European Prospective Investigation into Cancer and Nutrition (EPIC)

The European Prospective Investigation into Cancer and Nutrition (EPIC) is an ongoing multicenter prospective cohort study designed primarily to investigate the relationship between nutrition and cancer. The recruitment and baseline assessment of the EPIC cohort are described in detail elsewhere[1,2]. Between 1992 and 2000, 521,330 individuals from 10 European countries. In this project we included participants from France, Germany, Greece, Italy, the Netherlands, Norway, Spain and the United Kingdom were recruited. Participants completed self-administered questionnaires on their diet, lifestyle and medical history. Height and weight of individuals were measured using standard protocols. Of the 521,330 individuals, 385,747 individuals provided a blood sample. Blood fractions were aliquoted into 0.5mL straws, which were heat sealed and stored in liquid nitrogen tanks at -196^o^C. All participants gave written informed consent. The study was approved by the ethics committee at the International Agency for Research on Cancer (Lyon, France) and the local ethics committee of the study centres.

Incident cancer cases were identified via linkage to population-based cancer registries (in Italy (except Naples), the Netherlands, Norway, Spain, and the United Kingdom) or by active follow-up (in France, Germany, Greece, and Naples), which involved a combination of methods, including review of health insurance records and cancer and pathology registries, as well as direct contact with participants and their next of kin. Participants were followed up from study entry until cancer diagnosis (except nonmelanoma skin cancer), death, emigration, or the end of follow-up.

We identified 635 eligible kidney cancer cases defined as participants who were diagnosed with Kidney cancer (with *International Classification of Diseases for Oncology, Second Edition*, code C64 and C65), excluding prevalent cases and cases with a history of another cancer (except nonmelanoma skin cancer). For each case, 1 control was chosen randomly from risk sets consisting of all cohort members who were alive and free of cancer (except nonmelanoma skin cancer) at the time of diagnosis of the index case. Matching criteria were country, sex, date of blood collection (±1 month, relaxed to ±5 months for sets without available controls), and date of birth (±1 year relaxed to ±5 years for sets without available controls). Written informed consent was obtained from all participants. In total, 635 matched case-controls pairs were included in our study.

## Northern Sweden Health and Disease study (NSHDS)

The Northern Sweden Health and Disease Study (NSHDS) includes several prospective cohorts[3]. The current study included study participants from the Västerbotten Intervention Project (VIP) , which is a sub-cohort within NSHDS. The ongoing VIP prospective cohort is an intervention study aimed at health promotion of the general population of the Västerbotten County in Sweden. In 1985, when VIP was started, all residents in the Västerbotten County were invited to participate by attending a health check-up at 40, 50 and 60 years of age. Participants were asked to complete a self-administered questionnaire that inquired about various population characteristics such as education, smoking habits, physical activity, diet, height and weight. Fasting blood samples were collected from participants during a medical examination. Blood specimens were collected and processed by centrifugation and separation and frozen at -80^o^C within 1 hr of collection. Plasma samples were stored in the Medical Biobank (Umea, Sweden).

Newly identified cancer cases were identified through linkage with the Swedish Cancer Registry and the local Northern Sweden Cancer Registry. Eligible controls were selected among those who were alive and cancer-free at the time of the case’s diagnosis and matched on birthdate (within 2.5 years), sex, blood draw date (within the same year), and fasting status. This study was approved by the Ethics Committee of the Faculty of medicine at Umea University, Umea Sweden. Written informed consent was obtained from all participants. In total, 163 incident kidney cancer cases and 163 individually matched controls were included in our study.

## The Trøndelag Health Study (HUNT)

The Trondelag Health Study (HUNT) includes repeated surveys of a large population-based cohort in Norway[4]. Data from 570 individuals aged 20 years and older from HUNT2 (1995 to 1997, n=416) and HUNT3 (2006 to 2008, n=154) were used in this study. Individuals who participated in both HUNT2 and HUNT3 were included as part of HUNT3. Blood samples were collected at the health examination stations and stored in the HUNT biobank at -70^o^C for later use. The self-administered questionnaires used in HUNT included medical history, smoking, alcohol consumption. Weight (kg) and height (cm) were measured in a standardized manner in HUNT2 and HUNT3. Body mass index (BMI) was calculated as weight (kg) divided by height (m) squared. Blood samples were collected at the time of participation as described in in the Cohort paper[4] and earlier in this section. The study was approved by the Regional Committee for Ethics in Medical Research, the National Directorate of Health, and by the Norwegian Data Inspectorate.

The mandatory reporting of cancer by physicians and hospitals to the Cancer Registry of Norway ([www.krefregisteret.no](http://www.krefregisteret.no)) provides information on incident cases of kidney cancer that occurred during follow-up. Incident kidney cancer cases were identified using ICD10 codes (C 64) and we acquired information on date of first diagnosis of participants from the Cancer Registry of Norway. All participants with previous cancer diagnosis were excluded. One randomly selected control, matched by sex, age ±2 years, date of blood collection (± 2 months) and time since last meal when blood sample was collected (fasting status). Controls were alive and did not have a cancer diagnosis at the diagnosis time of their index case. Written informed consent was obtained from all participants. In total, 254 matched case-controls pairs were included in our study.

## The Melbourne Collaborative Cohort Study (MCCS)

The Melbourne Collaborative Cohort Study (MCCS) is a prospective study of 41,513 healthy adult volunteers (24,469 women) aged between 27 and 76 years (99.3% aged 40-69) when recruited between 1990 and 1994[5,6]. At baseline, demographic characteristics and lifestyle factors were collected by interviewer-administered questionnaires (including smoking and alcohol consumption) while height, weight, and waist and hip circumferences were measured. Peripheral blood was drawn at recruitment (1990-1994) or at subsequent follow-up (2003-2007). The study was approved by Cancer Council Victoria’s Human Research Ethics Committee and performed in accordance with the institution’s ethical guidelines.

Cases of kidney cancer were identified by record linkage with the Victorian Cancer Registry that receives mandatory notification of all new cancer cases in Victoria, Australia. Diagnostic pathology reports were reviewed and classified according to the International Classification of Disease (ICD-0-3 WHO classification). Subjects with any history of kidney cancer before blood collection were excluded. Controls were individually matched to cases by age, sex and country of birth. Study participants provided informed consent in accordance with the Declaration of Helsinki. In total, 140 incident kidney cancer cases and 140 individually matched controls were included in our study.

## University of Tartu - Estonian Biobank (Estonian BB)

The Estonian Genome Center, The University of Tartu (EGCUT), cohort is a population biobank containing 5% of the Estonian adult population. Detailed description of the Estonian cohort was described previously[7]. The age, sex and geographical distribution of the 152,000 participants closely reflect those of the Estonian adult population. EGCUT can link its own database with the national electronic databases (eight total) to constantly update the phenotype information of the participants. Every entry in the biobank consists of: (i) biological samples, (ii) answers to the questions of a computer-assisted personal interview conducted at the doctor’s office (including questions about smoking and alcohol consumption), (iii) objective measurements performed at the doctor’s office (including weight, height, waist and hip circumferences and blood pressure), (iv) electronic health data from various databases, (v) genotype data from array genotyping, exome sequencing, or whole-genome sequencing, and (vi) biomedical data obtained by performing various assays on the material collected. Written informed consent was obtained from all participants for the baseline and follow-up investigations.

Kidney cancer cases were identified through national cancer registries and through independent review of medical records. For diagnosis of kidney cancer, we used the ICD-10 C64.0 code. For each case, we selected 1 random control, matching on age at sample collection, sex and time of blood collection. Controls were individuals who were alive and without a diagnosis of kidney cancer at time of the case’s diagnosis date. In total, 115 matched case-controls pairs were included in our study

# Metabolite data acquisition

## Biocrates

The targeted metabolomics approach was based on LC-ESI-MS/MS and FIA-ESI-MS/MS measurements by AbsoluteIDQ p180 Kit (BIOCRATES Life Sciences AG, Innsbruck, Austria).

The assay allows simultaneous quantification of 188 metabolites out of 10 µL plasma or serum, and includes free carnitine, 39 acylcarnitines (Cx:y), 21 amino acids (19 proteinogenic + citrulline + ornithine), 21 biogenic amines, hexoses (sum of hexoses – about 90-95 % glucose), 90 glycerophospholipids (14 lysophosphatidylcholines (lysoPC) and 76 phosphatidylcholines (PC)), and 15 sphingolipids (SMx:y). The abbreviations Cx:y are used to describe the total number of carbons and double bonds of all chains, respectively (for more details see 1). The method of AbsoluteIDQ p180 Kit has been proven to be in conformance with the EMEA-Guideline "Guideline on bioanalytical method validation (July 21st 2011”) [8], which implies proof of reproducibility within a given error range. The long-time stability of plasma metabolites during storage at -80 °C and the performance of the targeted-metabolomics platform using the AbsoluteIDQ p180 Kit have been evaluated in [9].

In the IARC laboratory, a liquid chromatography-tandem mass spectrometry system (Agilent UHPLC-1290/Sciex QTRAP5500 (AB Sciex, Framingham, MA, USA) was used to measure metabolites levels. For the LC-part, compound identification and quantification were based on scheduled multiple reaction monitoring measurements (sMRM). Sample preparation and LC-MS/MS measurements were performed as described in the manufacturer in manual UM-P180-Sciex-13. Analytical specifications for the limit of detection (LOD) and evaluated quantification ranges, further LOD for semiquantitative measurements, identities of quantitative and semiquantitative metabolites, specificity, potential interferences, linearity, precision and accuracy, reproducibility and stability were described in Biocrates manual AS-P180. The LODs were set to three times the values of the zero samples (phosphate buffered saline solution). The lower and upper limits of quantification were determined experimentally by Biocrates.

In the Helmholtz Zentrum München, an API4000 mass spectrometer (Sciex Deutschland GmbH, Darmstadt, Germany) was used to measure metabolites levels. The assay procedures of the AbsoluteIDQ p180 Kit as well as the metabolite nomenclature have been described in detail previously[10]. Sample handling was performed by a Hamilton Microlab STARTM robot (Hamilton Bonaduz AG, Bonaduz, Switzerland) and a Ultravap nitrogen evaporator (Porvair Sciences, Leatherhead, U.K.), beside standard laboratory equipment. Mass spectrometric analyses were done on an API 4000 triple quadrupole system (Sciex Deutschland GmbH, Darmstadt, Germany) equipped with a 1200 Series HPLC (Agilent Technologies Deutschland GmbH, Böblingen, Germany) and a HTC PAL auto sampler (CTC Analytics, Zwingen, Switzerland) controlled by the software Analyst 1.6.2. Data evaluation for quantification of metabolite concentrations and quality assessment was performed with the software MultiQuant 3.0.1 (Sciex) and the MetIDQ™ software package, which is an integral part of the AbsoluteIDQ Kit. Metabolite concentrations were calculated using internal standards and reported in µM.

## Metabolon

All samples were maintained at -80^o^C until processed. Samples were prepared with use of an automated MicroLab STAR system (Hamilton Company, Reno, NV, USA). For quality control (QC), a pooled sample from all experimental samples was used throughout the experiment, and a mixture of Metabolon QC standards were spiked into all experimental samples to monitor instrument performance and chromatographic alignment. Samples were randomised prior to experimentation. Experiments were conducted on Waters Acuity ultra-performance liquid chromatography (UPLC) systems (Waters Corporation, Milford, MA, USA) using Thermo Scientific Q- Exactive high resolution/accurate mass spectrometer interfaced with a heated electrospray ionization (HESI-II) source and Orbitrap mass analyser (Thermo Fisher Scientific, MA, USA). The analysis platform used four methods for Ultrahigh Performance Liquid Chromatography- Tandem Mass Spectroscopy (UPLC-MS/MS) including a) positive ion mode electrospray ionisation (ESI), b) positive ion mode optimised for hydrophobic compounds, c) negative ion mode ESI and d) negative ionisation following elution from a hydrophilic interaction chromatography (HILIC) column. Scan time varied between methods and covered 70- 1000m/z.**:** Raw data was extracted, peak-identified and QC processed using Metabolon’s hardware and software. Metabolites were identified by comparison to the in-house Metabolon standard library using retention time, mass (m/z), adducts and MS/MS spectra. As experiments were conducted over multiple consecutive days, a data normalization step was performed to correct variation resulting from instrument inter-day tuning differences.

The cases and their matched controls were assayed within the same batches in order to avoid any effect of batch differences on the risk estimates.

Instrument variability was determined by calculating the median relative standard deviation (RSD) for the internal standards that were added to each sample prior to injection into the mass spectrometers. Overall process variability was determined by calculating the median RSD for all endogenous metabolites (i.e., non-instrument standards) present in the MTRX5 technical replicates (a large pool of human plasma maintained by Metabolon that has been characterized extensively).

Values for instrument and process variability meet Metabolon’s acceptance criteria: median RSD for internal standards were 5% and 4% for EPIC and NSHDS samples, respectively; median RSD for endogenous biochemicals were 11% for both EPIC and NSHDS.

# Data sources for Mendelian randomization analyses

## BMI GWAS

Summary-level GWAS data for BMI was obtained from a 2018 meta-analysis of GWASs of BMI [11] (downloaded from: <https://portals.broadinstitute.org/collaboration/giant/index.php/GIANT_consortium_data_files#2018_GIANT_and_UK_BioBank_Meta-analysis>). This analysis was a fixed-effects meta-analysis combining results from a GWAS of BMI performed among 456,426 participants from the UK Biobank (adjusted for age, sex, recruitment center, genotyping batch and 10 genetic principal components) and results from a BMI GWAS published by the GIANT (Genetic Investigation of Anthropometric Traits)[12] consortium, which included 253,288 participants from 79 studies (adjusted for age, sex, and study specific covariates). For UK Biobank, BMI (weight in kg per height in metres squared) was measured during the initial assessment centre visit whereas for the BMI GWAS conducted by the GIANT consortium, BMI was either measured or self-reported.

## Metabolite GWAS

Summary-level GWAS data for 174 Biocrates metabolites [13] and 913 Metabolon metabolites were used. The metabolite GWAS data used in the MR analyses, are available via [www.omicscience.org](http://www.omicscience.org) for all Biocrates and a subset of Metabolon metabolites. Metabolite associations for the BMI-associated and dental disease-associated SNPs used in the Mendelian randomization analyses are available to download from at the University of Bristol data repository, data.bris, at https://doi.org/10.5523/bris.33bq35s9lbos026r1xukxijoqu.

*Biocrates:*

The GWAS meta-analysis for the 174 Biocrates metabolites was a fixed-effects meta-analysis combining results from the Fenland cohort [14] (maximum N= 9736, available at: <https://omicscience.org/apps/crossplatform/>) (metabolites profiled by the Biocrates p180 kit and measured using mass spectrometry) with those from the EPIC-Norfolk [15] (maximum N=5841) and INTERVAL studies [16] (maximum N=40,818) (metabolites were profiled using mass spectrometry (Metabolon Discovery HD4 platform) and proton nuclear magnetic resonance (^1^H-NMR) spectroscopy). Ten of the 174 Biocrates metabolites were covered across all platforms, while 38 were available on the Biocrates and Metabolon platforms and 126 were unique to Biocrates. An overall z-score meta-analysis was also conducted by further integrating publicly available summary statistics from GWAS of the same metabolites measured using mass spectrometry (with Biocrates or Metabolon platforms[17,18]) or ^1^H-NMR spectroscopy [18] (N=ranged from 8,569 to 86,507 for different metabolites, available at: <https://omicscience.org/apps/crossplatform/>).

Genotyping in Fenland was performed using Affymetrix SNP5.0 and Affymetrix Axiom and genotype imputation was performed using 1000 Genomes Phase 1v3 or phase 3 reference panels. In EPIC-Norfolk, genotype imputation was performed using 1000 Genomes Phase 3 reference panels. Genotyping in INTERVAL was performed using Affymetrix Axiom and imputation was performed using the 1000 Genomes Phase 3 (May 2013)-UK10K reference imputation panel. For Fenland and EPIC-Norfolk, GWAS analyses were carried out using BOLT-LMM and SNPTEST adjusting for age, sex and study-specific covariates in mixed linear models. For the GWAS conducted in INTERVAL, phenotype residuals were corrected for age, gender, metabolon batch, INTERVAL centre, plate number, appointment month, the lag time between the blood donation appointment and sample processing, and the first 5 ancestry principal components.

For the pleiotropy analyses, SNPs associated with metabolites at *p*<4.9x10^-10^ (conventional threshold of genome-wide significance corrected for 102 tests which corresponded to the number of principal components that explained 95% of the variance of the 174 metabolites in the Fenland cohort) were identified from the overall z-score meta-analysis. The estimated effect sizes for each of the metabolite-associated SNPs were then obtained from the three-cohort meta-analysis (Fenland and, when available, EPIC-Norfolk and/or INTERVAL) and only metabolite-associated SNPs with a *p*<5x10^-08^ in the three-cohort meta-analysis was included in the pleiotropy analyses. For the MR analyses, the metabolite associations for the BMI-associated SNPs or dental disease-associated SNPs were obtained from the three-cohort meta-analysis.

*Metabolon:*

A GWAS of metabolon metabolite levels was performed using samples from the EPIC-Norfolk [15] and INTERVAL studies [19]. 14,296 participants were included in a *discovery* set (5,841 from EPIC-Norfolk; 8,455 from INTERVAL) and 5,698 from EPIC-Norfolk in a *validation* set. Metabolites were measured using the Metabolon DiscoveryHD4 platform (Metabolon, Inc., Durham, USA), from plasma samples collected at baseline. A total of 913 metabolites measured in at least 100 participants in each study were taken forward for GWAS analysis. Metabolite measures were median normalised for run day, log transformed, winsorised to 5 standard deviations, before being regressed against age, sex and study specific variables using linear regression. Residuals from this regression were standardised (mean 0, standard deviation 1) and used for further analysis. Genotyping was performed using the Affymetrix Axiom UK Biobank genotyping array. In INTERVAL, genotype imputation was performed using the combined UK10K+1000 Genomes Phase 3 reference panel. In EPIC-Norfolk, imputation was performed using the Haplotype Reference Consortium reference panel, with additional variants imputed using the UK10K+1000 Genomes Phase 3 reference panel.

Association analyses were performed using BOLT-LMM [20] or SNPTEST [21,22] separately in each study and combined using inverse variance weighted fixed effect meta-analysis methods implemented in METAL [23]. Genome-wide significant (p < 5 x 10^-8^) lead regional associations that were directionally consistent and significant at p < 0.01 in both studies were considered validated if they were significant at p < 5.48 x 10^-11^ (p < 5 x 10^-8^ Bonferroni corrected for 913 metabolites) and directionally consistent in a meta-analysis including the independent validation samples, as described above. To identify independent associations, exact conditional analyses were then performed using forward stepwise regression with a significance threshold of p < 1.25 x 10^-8^. For the current study, unconditional effect estimates for both primary and conditionally independent associations were used. In analyses to assess pleiotropy of potential instruments, we obtained the effect estimates from the unconditional analysis and all SNPs used had a *p<5* x10^-08^ in the unconditional analysis.

## Dental disease GWAS

Summary-level data for dental disease was obtained from a 2019 meta-analysis of GWASs of dental disease (DMFS (Decayed, Missing and Filled tooth Surfaces); N=26,792 from 9 studies) and dentures (n_case_= 77,714 and n_controls_ = 383,317) [24] (downloaded from: <https://data.bris.ac.uk/data/dataset/2j2rqgzedxlq02oqbb4vmycnc2>). This analysis was a fixed effects meta-analysis combining results from a GWAS of dental disease performed in the UK Biobank (adjusted for age, age-squared, sex, genotyping batch) and a GWAS of dental disease conducted by GLIDE (Gene-Lifestyle Interactions in Dental Endpoints) (adjusted for age, age-squared, genetic principal components and other study-specific covariates). Dental disease. Self-reported measures of oral health were characterised in UK Biobank while clinical dental records were used to calculate DMFS/dentures in GLIDE.

# References

1. Riboli E, Kaaks R. The EPIC Project: rationale and study design. European Prospective Investigation into Cancer and Nutrition. Int J Epidemiol. 1997;26 Suppl 1:S6-14. Epub 1997/01/01. PMID: 9126529.

2. Riboli E, Hunt KJ, Slimani N, Ferrari P, Norat T, Fahey M, et al. European Prospective Investigation into Cancer and Nutrition (EPIC): study populations and data collection. Public Health Nutr. 2002;5(6B):1113-24. Epub 2003/03/18. doi: 10.1079/PHN2002394. PMID: 12639222.

3. Hallmans G, Agren A, Johansson G, Johansson A, Stegmayr B, Jansson JH, et al. Cardiovascular disease and diabetes in the Northern Sweden Health and Disease Study Cohort - evaluation of risk factors and their interactions. Scand J Public Health Suppl. 2003;61:18-24. doi: 10.1080/14034950310001432. PMID: 14660243.

4. Krokstad S, Langhammer A, Hveem K, Holmen TL, Midthjell K, Stene TR, et al. Cohort Profile: the HUNT Study, Norway. Int J Epidemiol. 2013;42(4):968-77. Epub 2012/08/11. doi: 10.1093/ije/dys095. PMID: 22879362.

5. Milne RL, Fletcher AS, MacInnis RJ, Hodge AM, Hopkins AH, Bassett JK, et al. Cohort Profile: The Melbourne Collaborative Cohort Study (Health 2020). Int J Epidemiol. 2017;46(6):1757-i. Epub 2017/06/24. doi: 10.1093/ije/dyx085. PMID: 28641380.

6. Giles GG, English DR. The Melbourne Collaborative Cohort Study. IARC Sci Publ. 2002;156:69-70. PMID: 12484128.

7. Leitsalu L, Haller T, Esko T, Tammesoo ML, Alavere H, Snieder H, et al. Cohort Profile: Estonian Biobank of the Estonian Genome Center, University of Tartu. Int J Epidemiol. 2015;44(4):1137-47. Epub 2014/02/13. doi: 10.1093/ije/dyt268. PMID: 24518929.

8. Guideline on bioanalytical method validation. Committee for Medicinal Products for Human Use (CHMP). 2011;EMEA/CHMP/EWP/192217/2009 (Rev. 1 Corr. 2). Epub 21 July 2011.

9. Haid M, Muschet C, Wahl S, Romisch-Margl W, Prehn C, Moller G, et al. Long-Term Stability of Human Plasma Metabolites during Storage at -80 degrees C. J Proteome Res. 2018;17(1):203-11. Epub 2017/10/25. doi: 10.1021/acs.jproteome.7b00518. PMID: 29064256.

10. Zukunft S, Sorgenfrei M, Prehn C, Moller G, Adamski J. Targeted Metabolomics of Dried Blood Spot Extracts. Chromatographia. 2013;76(19-20):1295-305. doi: 10.1007/s10337-013-2429-3. PMID: WOS:000324825100011.

11. Yengo L, Sidorenko J, Kemper KE, Zheng Z, Wood AR, Weedon MN, et al. Meta-analysis of genome-wide association studies for height and body mass index in approximately 700000 individuals of European ancestry. Hum Mol Genet. 2018;27(20):3641-9. Epub 2018/08/21. doi: 10.1093/hmg/ddy271. PMID: 30124842.

12. Locke AE, Kahali B, Berndt SI, Justice AE, Pers TH, Day FR, et al. Genetic studies of body mass index yield new insights for obesity biology. Nature. 2015;518(7538):197-206. Epub 2015/02/13. doi: 10.1038/nature14177. PMID: 25673413.

13. Lotta LA, Pietzner M, Stewart ID, Wittemans LBL, Li C, Bonelli R, et al. A cross-platform approach identifies genetic regulators of human metabolism and health. Nat Genet. 2021;53(1):54-64. Epub 2021/01/09. doi: 10.1038/s41588-020-00751-5. PMID: 33414548.

14. Lindsay T, Westgate K, Wijndaele K, Hollidge S, Kerrison N, Forouhi N, et al. Descriptive epidemiology of physical activity energy expenditure in UK adults (The Fenland study). Int J Behav Nutr Phys Act. 2019;16(1):126. Epub 2019/12/11. doi: 10.1186/s12966-019-0882-6. PMID: 31818302.

15. Day N, Oakes S, Luben R, Khaw KT, Bingham S, Welch A, et al. EPIC-Norfolk: study design and characteristics of the cohort. European Prospective Investigation of Cancer. Br J Cancer. 1999;80 Suppl 1:95-103. Epub 1999/08/31. PMID: 10466767.

16. Moore C, Sambrook J, Walker M, Tolkien Z, Kaptoge S, Allen D, et al. The INTERVAL trial to determine whether intervals between blood donations can be safely and acceptably decreased to optimise blood supply: study protocol for a randomised controlled trial. Trials. 2014;15:363. Epub 2014/09/19. doi: 10.1186/1745-6215-15-363. PMID: 25230735.

17. Shin SY, Fauman EB, Petersen AK, Krumsiek J, Santos R, Huang J, et al. An atlas of genetic influences on human blood metabolites. Nat Genet. 2014;46(6):543-50. Epub 2014/05/13. doi: 10.1038/ng.2982. PMID: 24816252.

18. Draisma HHM, Pool R, Kobl M, Jansen R, Petersen AK, Vaarhorst AAM, et al. Genome-wide association study identifies novel genetic variants contributing to variation in blood metabolite levels. Nat Commun. 2015;6:7208. Epub 2015/06/13. doi: 10.1038/ncomms8208. PMID: 26068415.

19. Di Angelantonio E, Thompson SG, Kaptoge S, Moore C, Walker M, Armitage J, et al. Efficiency and safety of varying the frequency of whole blood donation (INTERVAL): a randomised trial of 45 000 donors. Lancet. 2017;390(10110):2360-71. Epub 2017/09/25. doi: 10.1016/S0140-6736(17)31928-1. PMID: 28941948.

20. Loh PR, Tucker G, Bulik-Sullivan BK, Vilhjalmsson BJ, Finucane HK, Salem RM, et al. Efficient Bayesian mixed-model analysis increases association power in large cohorts. Nat Genet. 2015;47(3):284-90. Epub 2015/02/03. doi: 10.1038/ng.3190. PMID: 25642633.

21. Marchini J, Howie B. Genotype imputation for genome-wide association studies. Nat Rev Genet. 2010;11(7):499-511. Epub 2010/06/03. doi: 10.1038/nrg2796. PMID: 20517342.

22. Wellcome Trust Case Control C. Genome-wide association study of 14,000 cases of seven common diseases and 3,000 shared controls. Nature. 2007;447(7145):661-78. Epub 2007/06/08. doi: 10.1038/nature05911. PMID: 17554300.

23. Willer CJ, Li Y, Abecasis GR. METAL: fast and efficient meta-analysis of genomewide association scans. Bioinformatics. 2010;26(17):2190-1. Epub 2010/07/10. doi: 10.1093/bioinformatics/btq340. PMID: 20616382.

24. Shungin D, Haworth S, Divaris K, Agler CS, Kamatani Y, Keun Lee M, et al. Genome-wide analysis of dental caries and periodontitis combining clinical and self-reported data. Nat Commun. 2019;10(1):2773. Epub 2019/06/27. doi: 10.1038/s41467-019-10630-1. PMID: 31235808.
