# Supplementary material for: The blood metabolome of incident kidney cancer: A case–control study nested within the MetKid consortium
Source: PLoS Med. 2021 Sep 20;18(9):e1003786. doi: 10.1371/journal.pmed.1003786 (PMC8496779; doi:10.1371/journal.pmed.1003786)
Supplement: S1 Fig — QC, quality control. (DOCX) [file pmed.1003786.s005.docx]

**Figure S1. Overview of the quality control pipelines used for the metabolite measurements pre-processing**

**
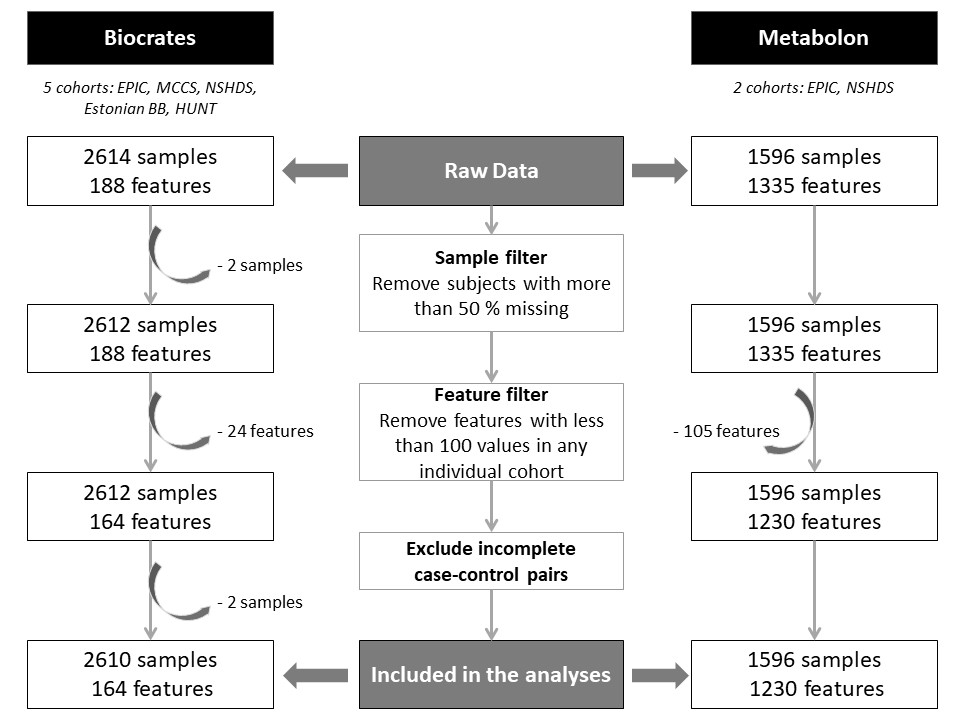
**

EPIC: The European Prospective Investigation into Cancer and Prevention; Estonian BB: University of Tartu- Estonian Biobank; HUNT: The Trøndelag Health Study; MCCS: The Melbourne Collaborative Cohort Study; NSHDS: Northern Sweden Health and Disease study.

*The number of features is the maximum number of features measured across all cohorts. Some are only measured in one cohort.*
