# Supplementary material for: The blood metabolome of incident kidney cancer: A case–control study nested within the MetKid consortium
Source: PLoS Med. 2021 Sep 20;18(9):e1003786. doi: 10.1371/journal.pmed.1003786 (PMC8496779; doi:10.1371/journal.pmed.1003786)
Supplement: S2 Fig — (DOCX) [file pmed.1003786.s006.docx]

**Figure S2. Heatmap of Pearson correlation coefficients for selected Biocrates (left) and Metabolon (right) metabolites.**


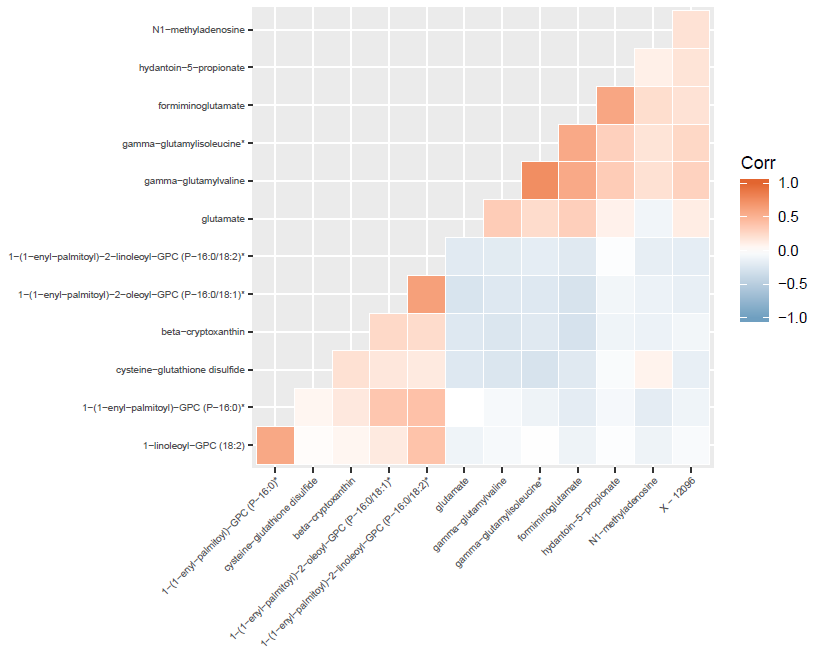

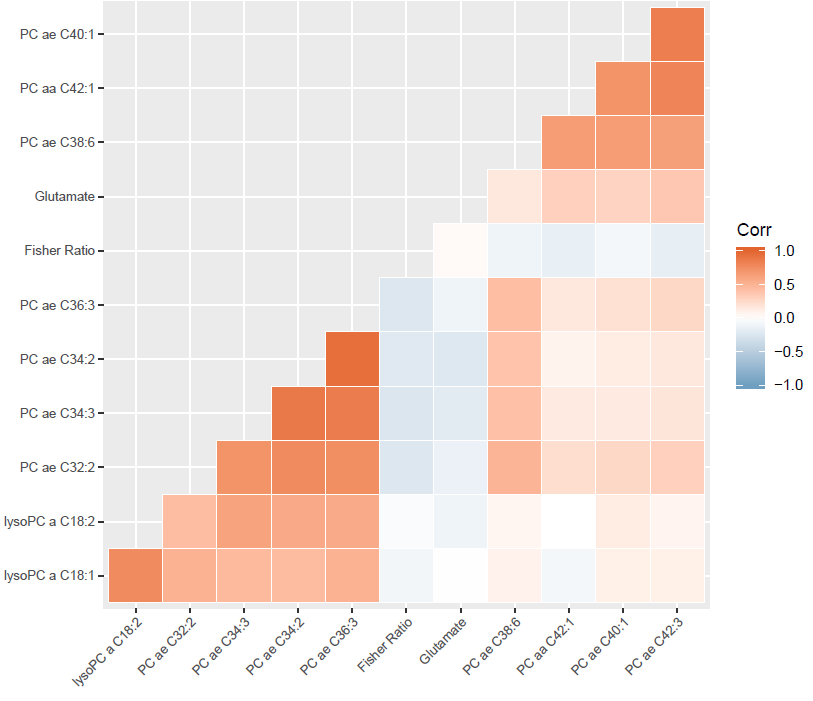


Corr: Pearson correlation coefficient
