# Supplementary material for: The blood metabolome of incident kidney cancer: A case–control study nested within the MetKid consortium
Source: PLoS Med. 2021 Sep 20;18(9):e1003786. doi: 10.1371/journal.pmed.1003786 (PMC8496779; doi:10.1371/journal.pmed.1003786)
Supplement: S3 Fig — Fig A: Forest plots depicts the kidney cancer risk association for the Fischer ratio stratified by risk factors. Fig B: Forest plots depicts the kidney cancer risk association for glutamate (Biocrates), stratified by risk factors. Fig C: Forest plots depicts the kidney cancer risk association for lysoPC a C18:1, stratified by risk factors. Fig D: Forest plots depicts the kidney cancer risk association for lysoPC a C18:2, stratified by risk factors. Fig E: Forest plots depicts the kidney cancer risk association for PC aa C42:1, stratified by risk factors. Fig F: Forest plots depicts the kidney cancer risk association for PC ae C32:2, stratified by risk factors. Fig G: Forest plots depicts the kidney cancer risk association for PC ae C34:2, stratified by risk factors. Fig H: Forest plots depicts the kidney cancer risk association for PC ae C34:3, stratified by risk factors. Fig I: Forest plots depicts the kidney cancer risk association for PC ae C36:3, stratified by risk factors. Fig J: Forest plots depicts the kidney cancer risk association for PC ae C38:6, stratified by risk factors. Fig K: Forest plots depicts the kidney cancer risk association for PC ae C40:1, stratified by risk factors. Fig L: Forest plots depicts the kidney cancer risk association for PC ae C42:3, stratified by risk factors. Fig M: Forest plots depicts the kidney cancer risk association for 1-(1-enyl-palmitoyl)-2-linoleoyl-GPC (P-16:0/18:2)*, stratified by risk factors. Fig N: Forest plots depicts the kidney cancer risk association for 1-(1-enyl-palmitoyl)-2-oleoyl-GPC (P-16:0/18:1)*, stratified by risk factors. Fig O: Forest plots depicts the kidney cancer risk association for 1-(1-enyl-palmitoyl)-GPC (P-16:0)*, stratified by risk factors. Fig P: Forest plots depicts the kidney cancer risk association for 1-linoleoyl-GPC (18:2), stratified by risk factors. Fig Q: Forest plots depicts the kidney cancer risk association for beta-cryptoxanthin, stratified by risk factors. Fig R: Forest plots depic [file pmed.1003786.s007.docx]

**Figures S3. Forest plots depicting the kidney cancer risk association for each metabolite deemed robustly associated with kidney cancer risk, stratified by specific** **kidney cancer risk factors.**

[Figure A. Forest plots depicts the kidney cancer risk association for the Fischer’s ratio stratified by risk factors. 2](#_Toc82021810)

[Figure B. Forest plots depicts the kidney cancer risk association for glutamate (Biocrates), stratified by risk factors. 3](#_Toc82021811)

[Figure C. Forest plots depicts the kidney cancer risk association for lysoPC a C18:1, stratified by risk factors. 4](#_Toc82021812)

[Figure D. Forest plots depicts the kidney cancer risk association for lysoPC a C18:2, stratified by risk factors. 5](#_Toc82021813)

[Figure E. Forest plots depicts the kidney cancer risk association for PC aa C42:1, stratified by risk factors. 6](#_Toc82021814)

[Figure F. Forest plots depicts the kidney cancer risk association for PC ae C32:2, stratified by risk factors. 7](#_Toc82021815)

[Figure G. Forest plots depicts the kidney cancer risk association for PC ae C34:2, stratified by risk factors. 8](#_Toc82021816)

[Figure H. Forest plots depicts the kidney cancer risk association for PC ae C34:3, stratified by risk factors. 9](#_Toc82021817)

[Figure I. Forest plots depicts the kidney cancer risk association for PC ae C36:3, stratified by risk factors. 10](#_Toc82021818)

[Figure J. Forest plots depicts the kidney cancer risk association for PC ae C38:6, stratified by risk factors. 11](#_Toc82021819)

[Figure K. Forest plots depicts the kidney cancer risk association for PC ae C40:1, stratified by risk factors. 12](#_Toc82021820)

[Figure L. Forest plots depicts the kidney cancer risk association for PC ae C42:3, stratified by risk factors. 13](#_Toc82021821)

[Figure M. Forest plots depicts the kidney cancer risk association for 1-(1-enyl-palmitoyl)-2-linoleoyl-GPC (P-16:0/18:2)*, stratified by risk factors. 14](#_Toc82021822)

[Figure N. Forest plots depicts the kidney cancer risk association for 1-(1-enyl-palmitoyl)-2-oleoyl-GPC (P-16:0/18:1)*, stratified by risk factors. 15](#_Toc82021823)

[Figure O. Forest plots depicts the kidney cancer risk association for 1-(1-enyl-palmitoyl)-GPC (P-16:0)*, stratified by risk factors. 16](#_Toc82021824)

[Figure P. Forest plots depicts the kidney cancer risk association for 1-linoleoyl-GPC (18:2), stratified by risk factors. 17](#_Toc82021825)

[Figure Q. Forest plots depicts the kidney cancer risk association for beta-cryptoxanthin, stratified by risk factors. 18](#_Toc82021826)

[Figure R. Forest plots depicts the kidney cancer risk association for cysteine-glutathione disulfide, stratified by risk factors. 19](#_Toc82021827)

[Figure S. Forest plots depicts the kidney cancer risk association for formiminoglutamate, stratified by risk factors. 20](#_Toc82021828)

[Figure T. Forest plots depicts the kidney cancer risk association for gamma-glutamylisoleucine*, stratified by risk factors. 21](#_Toc82021829)

[Figure U. Forest plots depicts the kidney cancer risk association for gamma-glutamylvaline, stratified by risk factors. 22](#_Toc82021830)

[Figure V. Forest plots depicts the kidney cancer risk association for glutamate (Metabolon), stratified by risk factors. 23](#_Toc82021831)

[Figure W. Forest plots depicts the kidney cancer risk association for hydantoin-5-propionate, stratified by risk factors. 24](#_Toc82021832)

[Figure X. Forest plots depicts the kidney cancer risk association for N1-methyladenosine, stratified by risk factors. 25](#_Toc82021833)

[Figure Y. Forest plots depicts the kidney cancer risk association for X-12096, stratified by risk factors. 26](#_Toc82021834)

### Figure A. Forest plots depicts the kidney cancer risk association for the Fischer’s ratio stratified by risk factors.

**
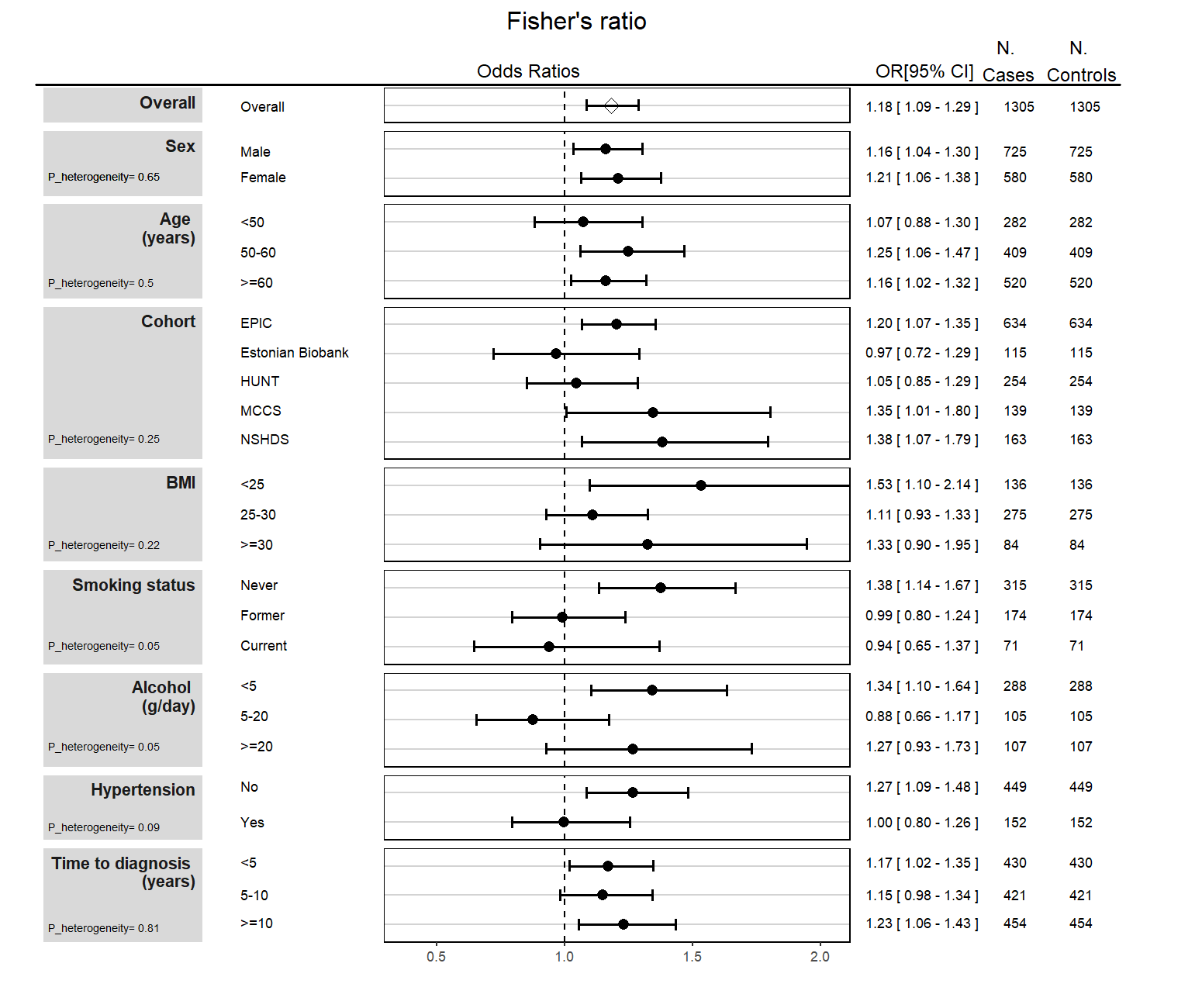
**

BMI: Body Mass Index; CI: Confidence Interval; d: days; g: grams; N.: number of participants; OR: Odds Ratio.

The Fischer’s ratio is a clinical indicator of liver metabolism and function, was calculated as the molar ratio of branched chain amino acids (leucine + isoleucine + valine) to aromatic amino acids (phenylalanine + tyrosine). Lower Fischer’s ratio values are associated with liver dysfunction.

### Figure B. Forest plots depicts the kidney cancer risk association for glutamate (Biocrates), stratified by risk factors.

**
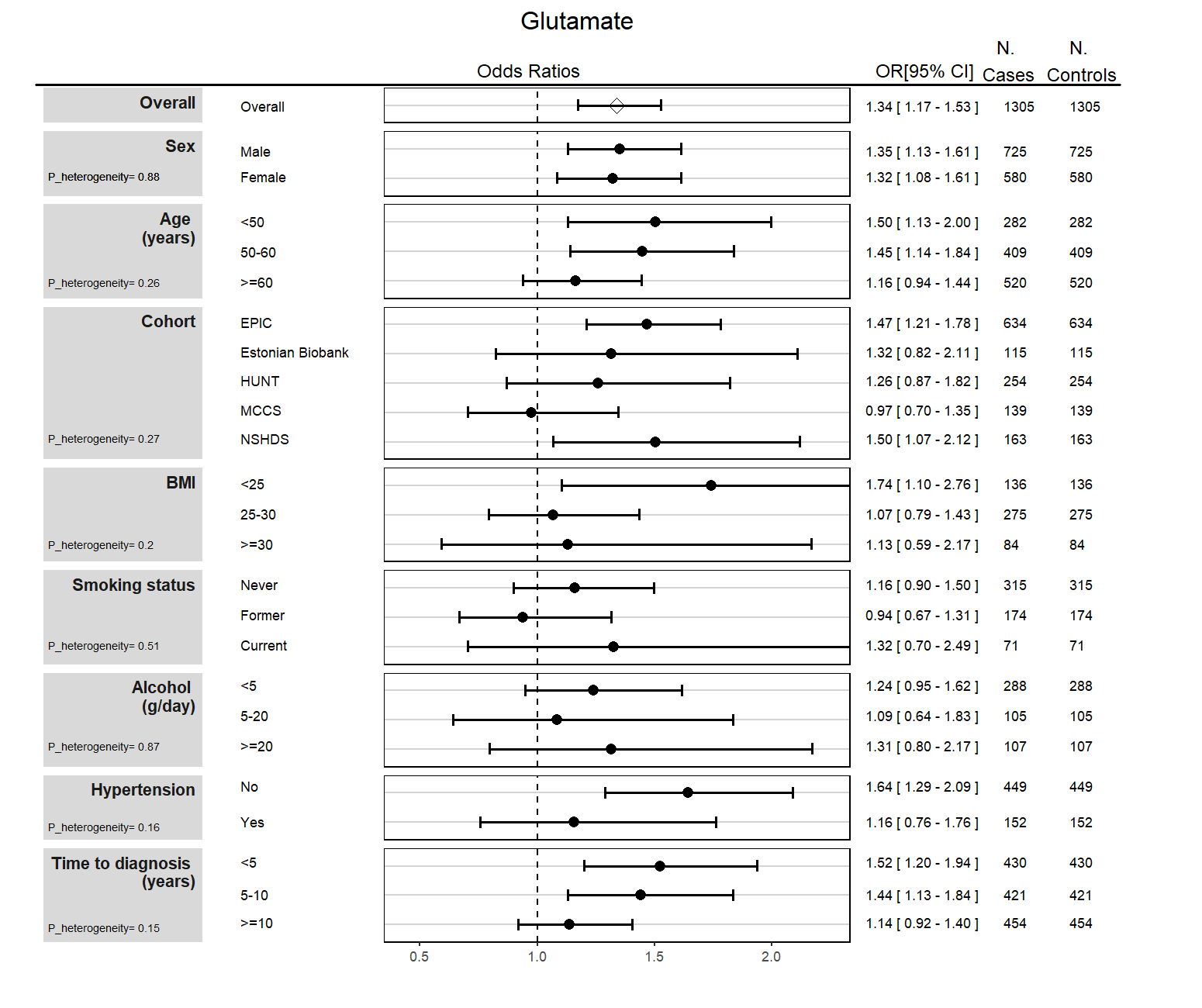
**

BMI: Body Mass Index; CI: Confidence Interval; d: days; g: grams; N.: number of participants; OR: Odds Ratio.

### Figure C. Forest plots depicts the kidney cancer risk association for lysoPC a C18:1, stratified by risk factors.

**
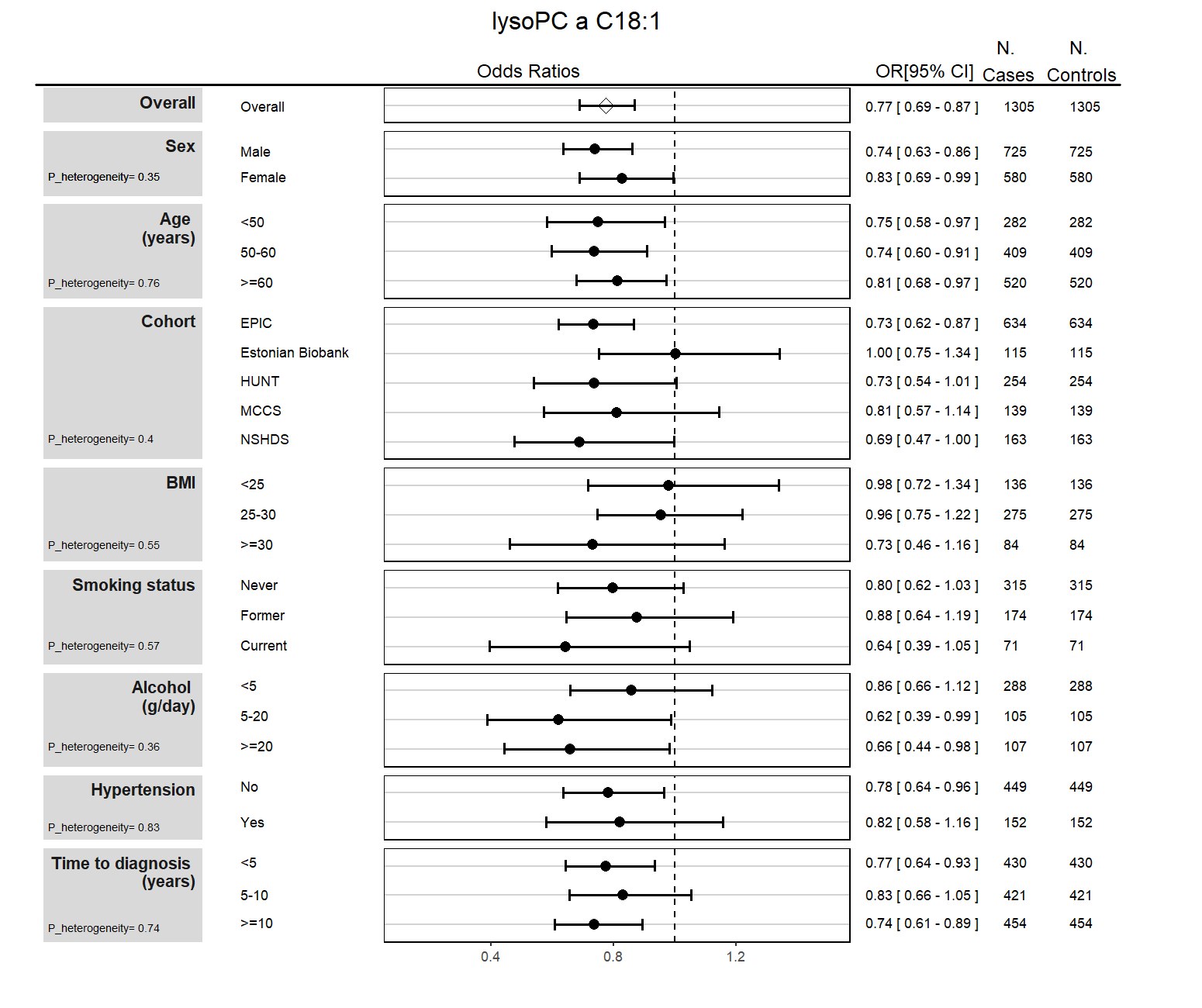
**

BMI: Body Mass Index; CI: Confidence Interval; d: days; g: grams; N.: number of participants; OR: Odds Ratio.

### Figure D. Forest plots depicts the kidney cancer risk association for lysoPC a C18:2, stratified by risk factors.

**
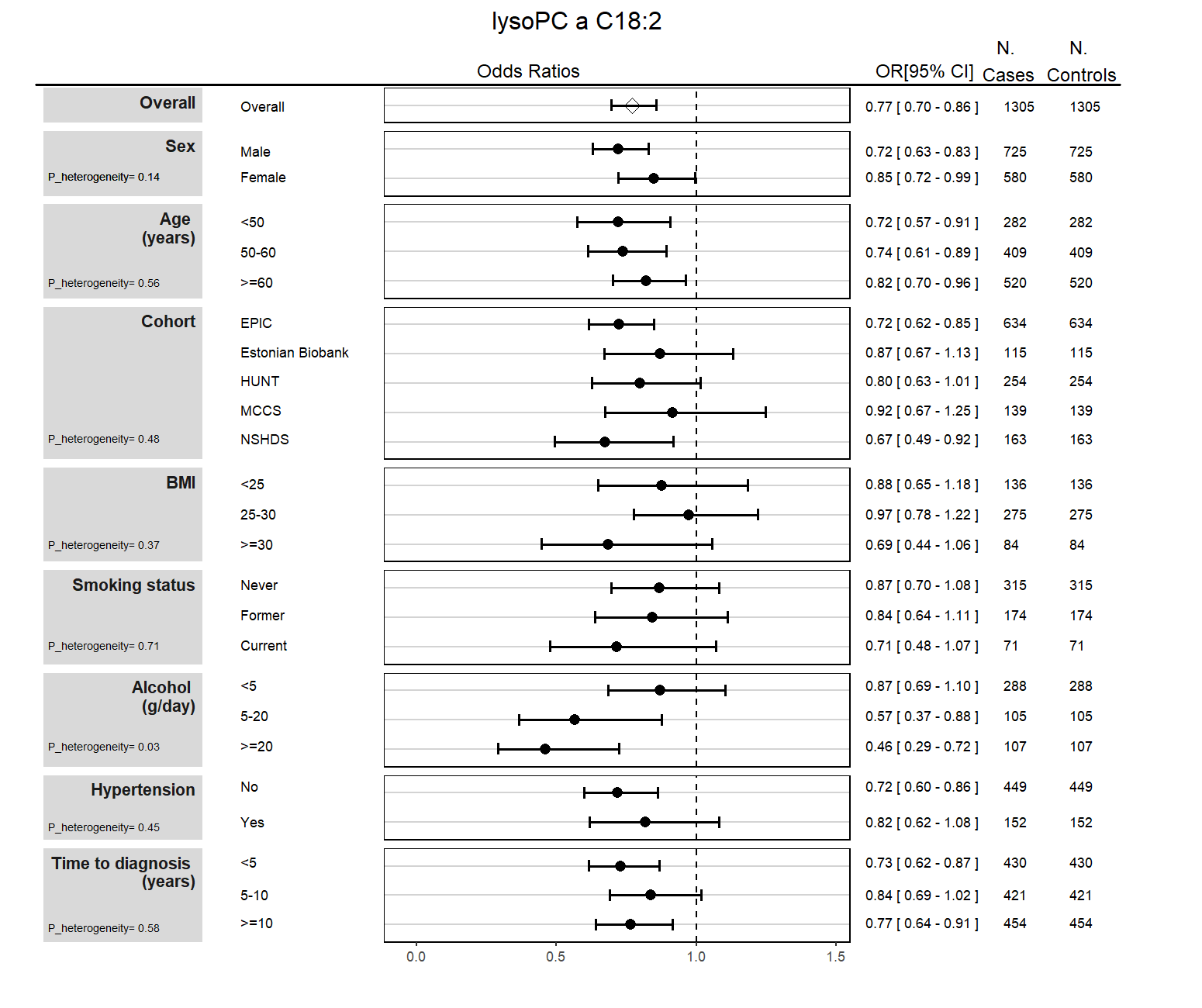
**

BMI: Body Mass Index; CI: Confidence Interval; d: days; g: grams; N.: number of participants; OR: Odds Ratio.

### Figure E. Forest plots depicts the kidney cancer risk association for PC aa C42:1, stratified by risk factors.

**
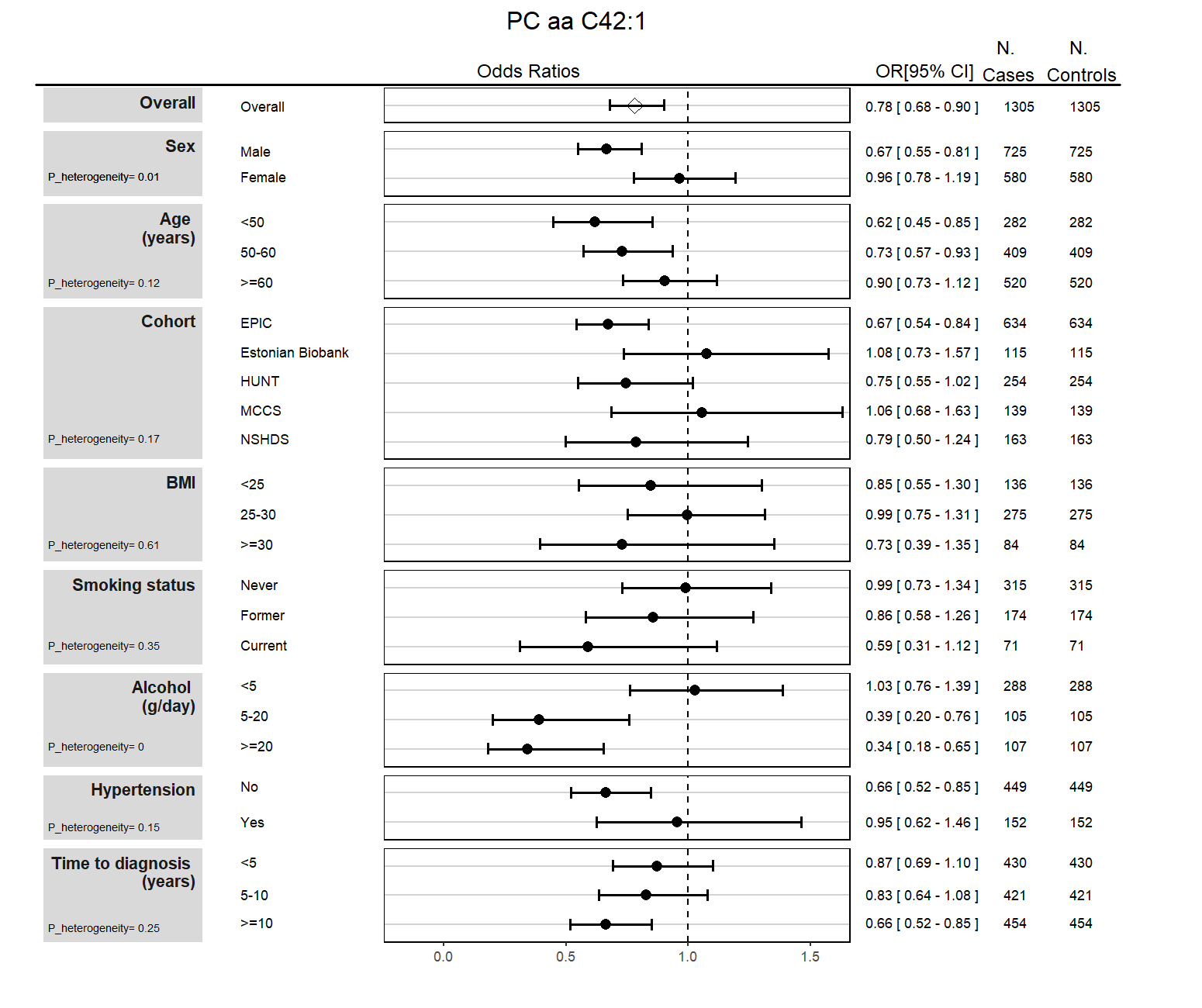
**

BMI: Body Mass Index; CI: Confidence Interval; d: days; g: grams; N.: number of participants; OR: Odds Ratio.

### Figure F. Forest plots depicts the kidney cancer risk association for PC ae C32:2, stratified by risk factors.

**
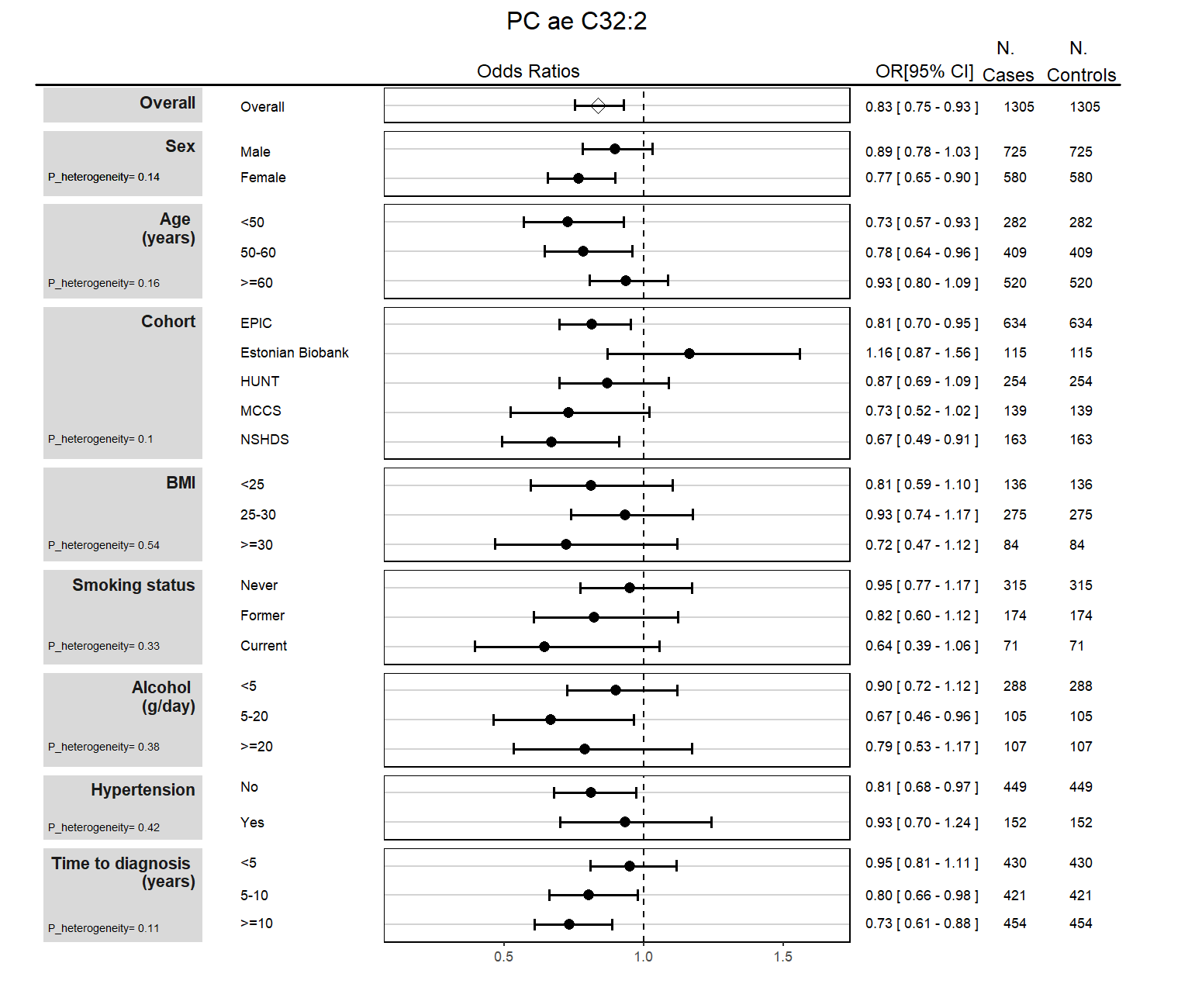
**

BMI: Body Mass Index; CI: Confidence Interval; d: days; g: grams; N.: number of participants; OR: Odds Ratio.

### Figure G. Forest plots depicts the kidney cancer risk association for PC ae C34:2, stratified by risk factors.

**
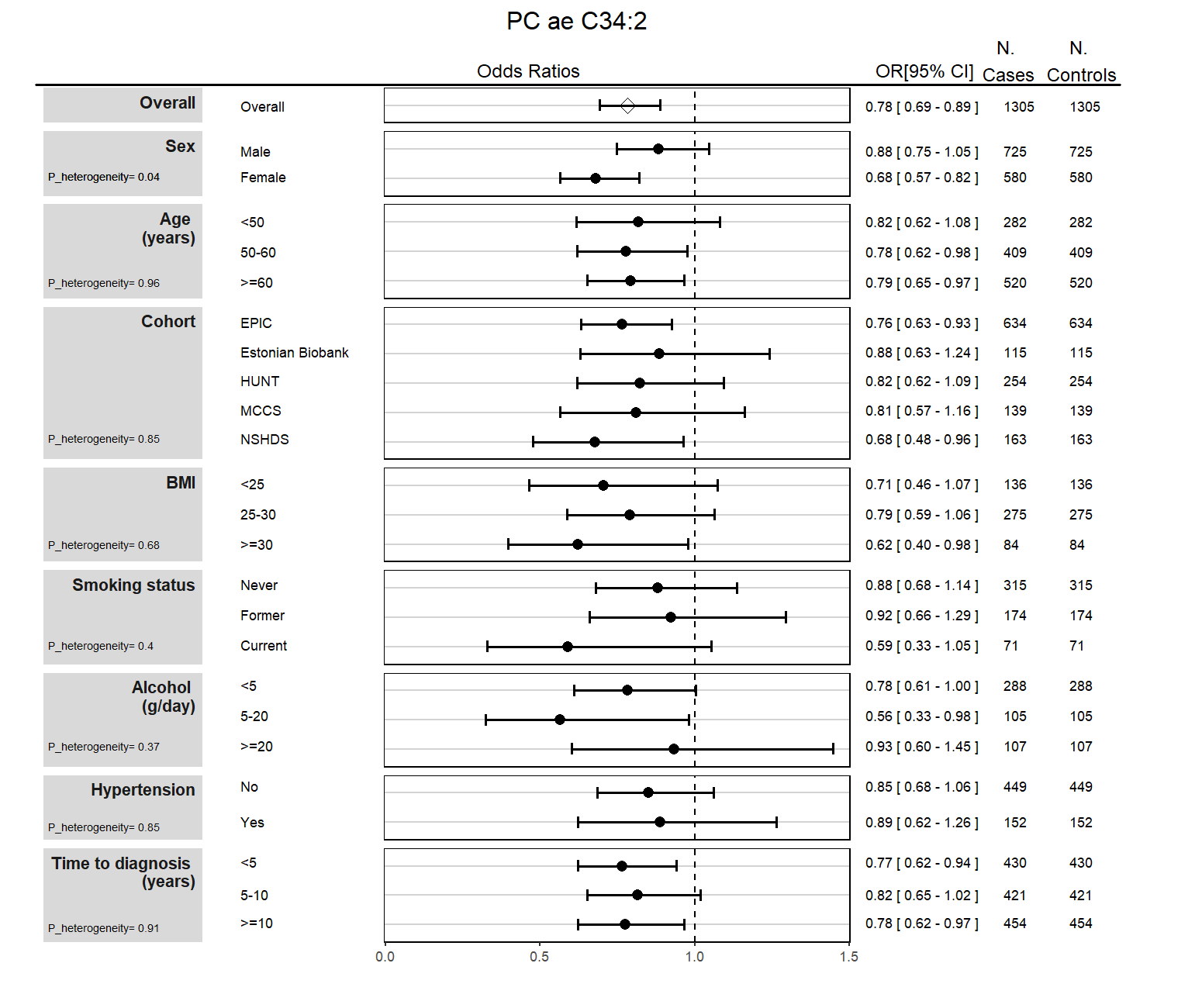
**

BMI: Body Mass Index; CI: Confidence Interval; d: days; g: grams; N.: number of participants; OR: Odds Ratio.

### Figure H. Forest plots depicts the kidney cancer risk association for PC ae C34:3, stratified by risk factors.

**
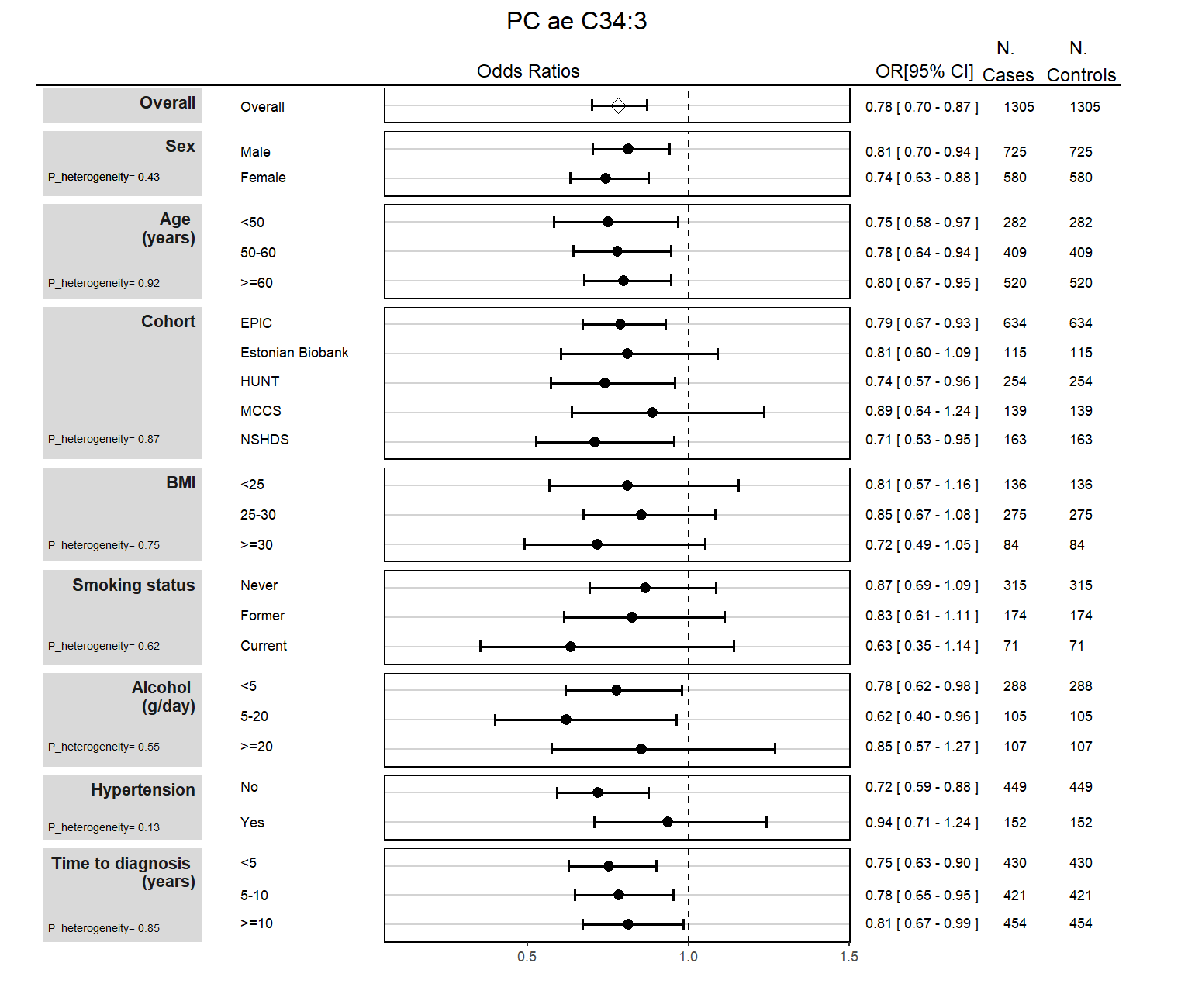
**

BMI: Body Mass Index; CI: Confidence Interval; d: days; g: grams; N.: number of participants; OR: Odds Ratio.

### Figure I. Forest plots depicts the kidney cancer risk association for PC ae C36:3, stratified by risk factors.

**
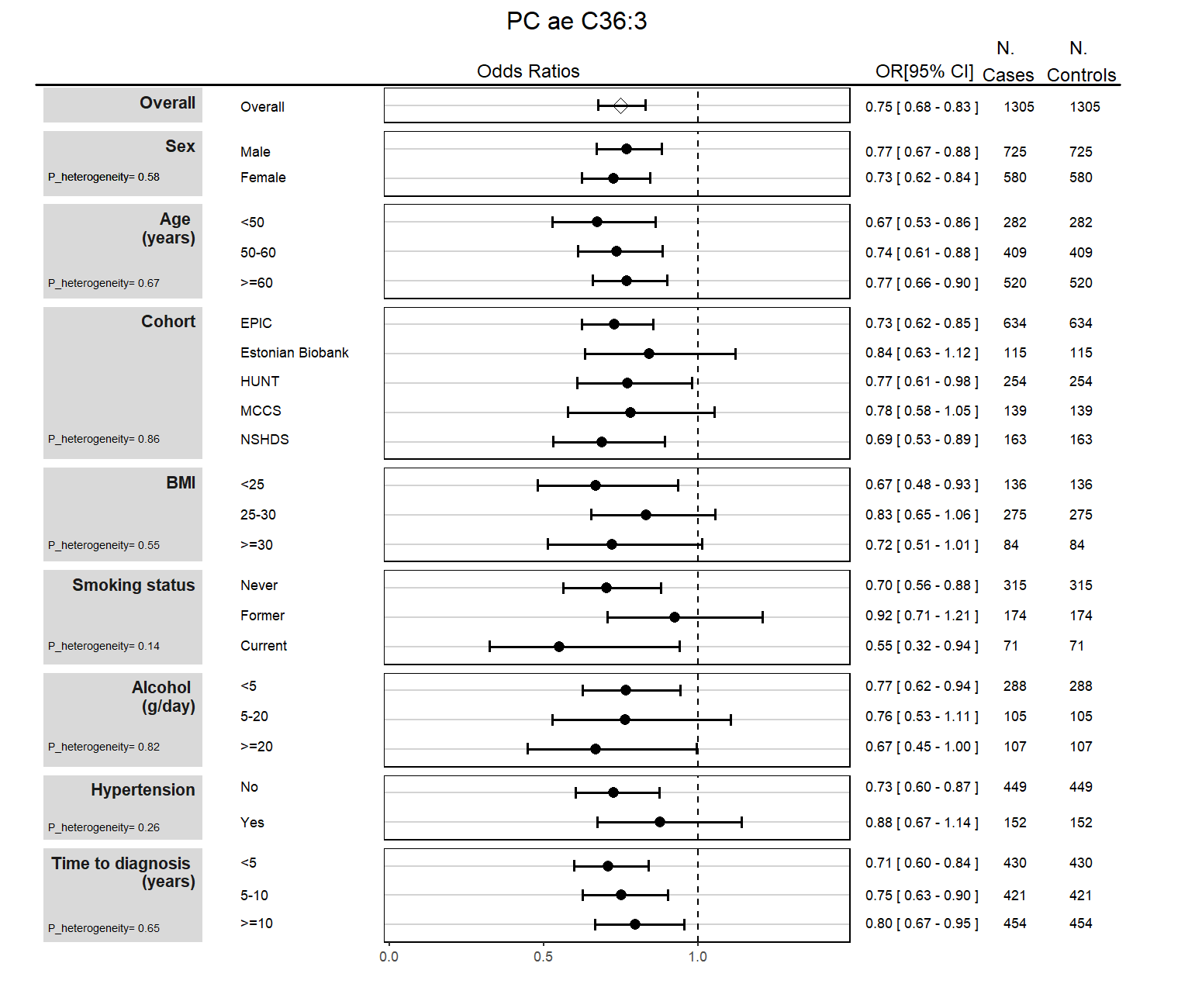
**

BMI: Body Mass Index; CI: Confidence Interval; d: days; g: grams; N.: number of participants; OR: Odds Ratio.

### Figure J. Forest plots depicts the kidney cancer risk association for PC ae C38:6, stratified by risk factors.

**
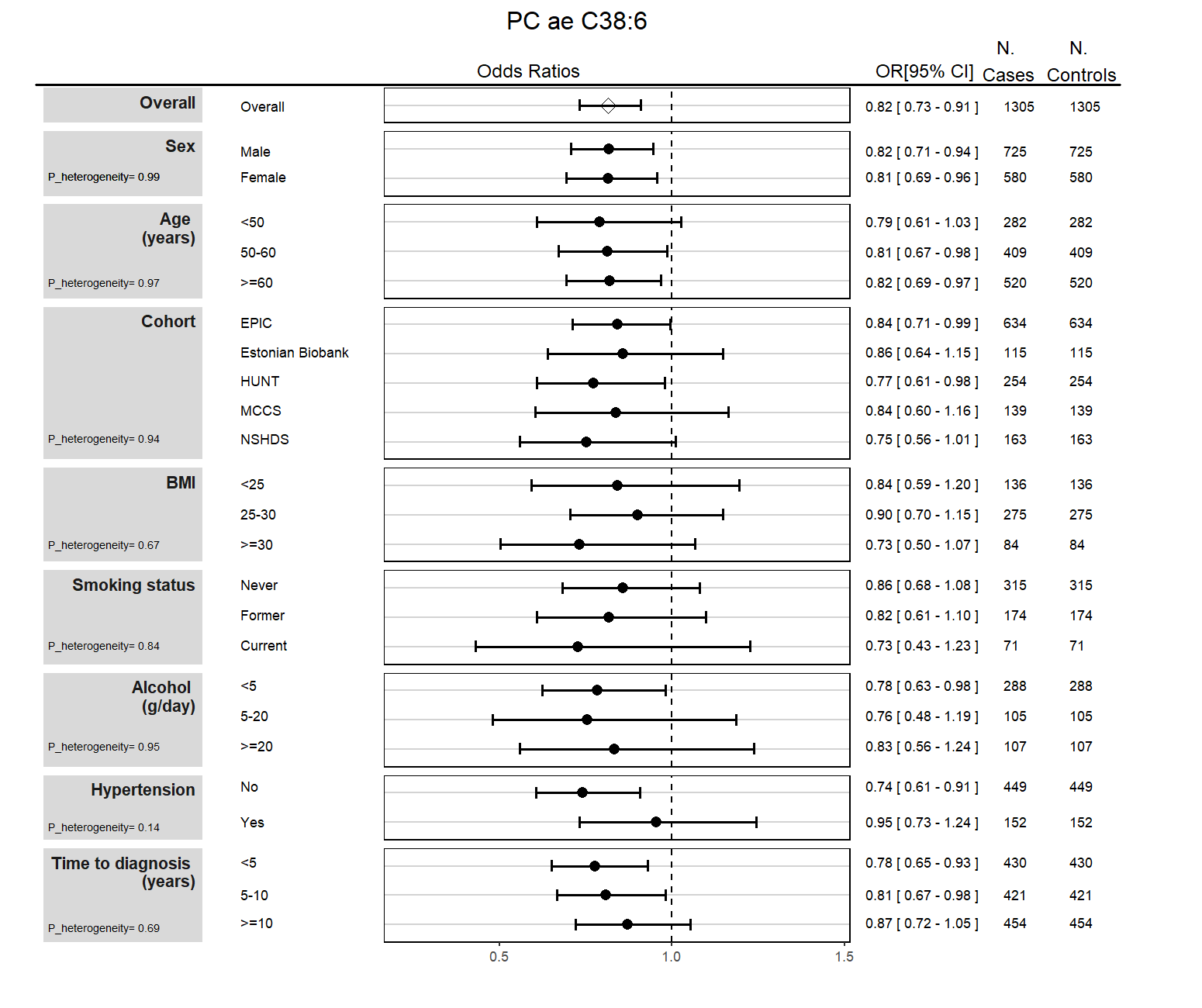
**

BMI: Body Mass Index; CI: Confidence Interval; d: days; g: grams; N.: number of participants; OR: Odds Ratio.

### Figure K. Forest plots depicts the kidney cancer risk association for PC ae C40:1, stratified by risk factors.

**
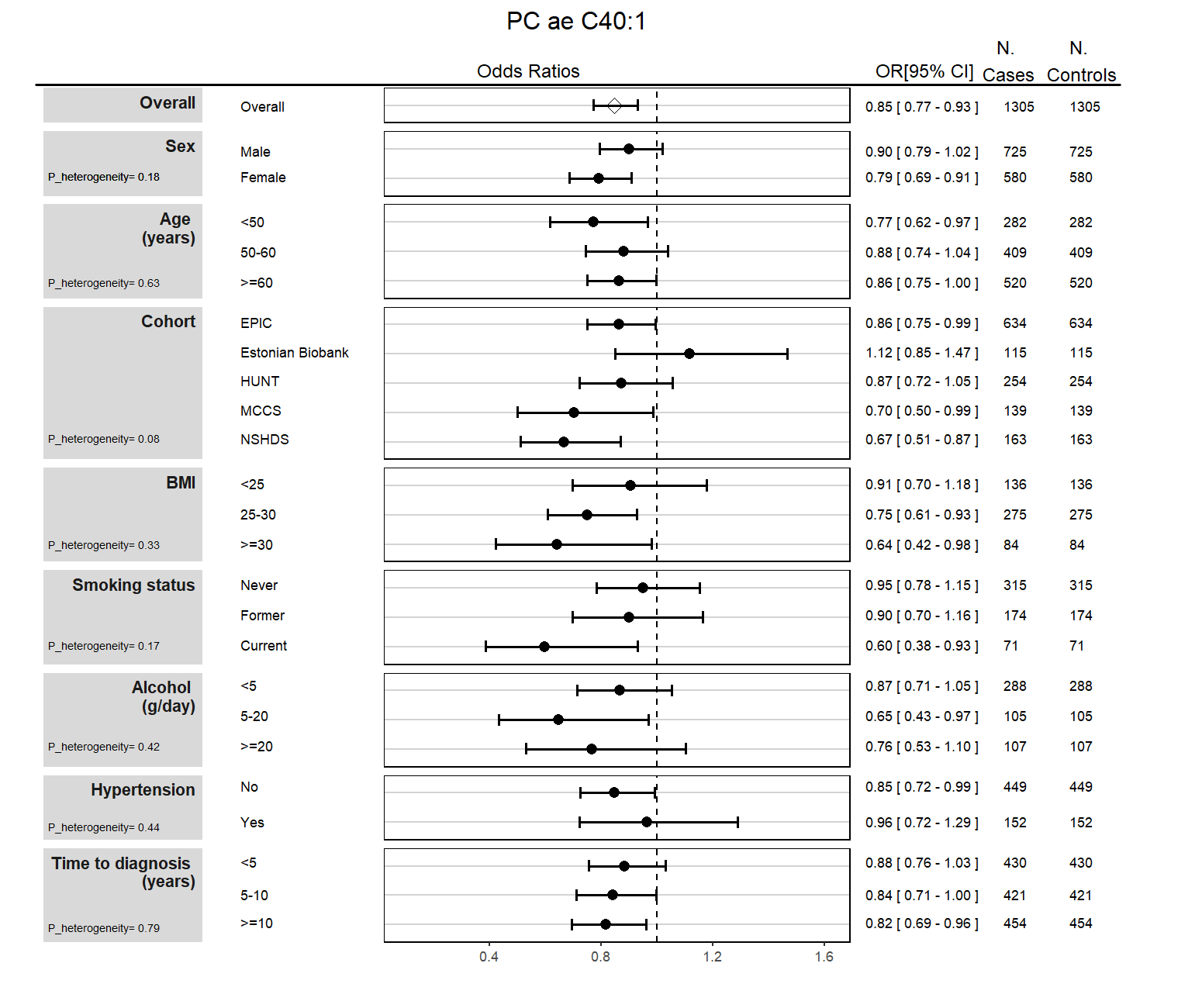
**

BMI: Body Mass Index; CI: Confidence Interval; d: days; g: grams; N.: number of participants; OR: Odds Ratio.

### Figure L. Forest plots depicts the kidney cancer risk association for PC ae C42:3, stratified by risk factors.

**
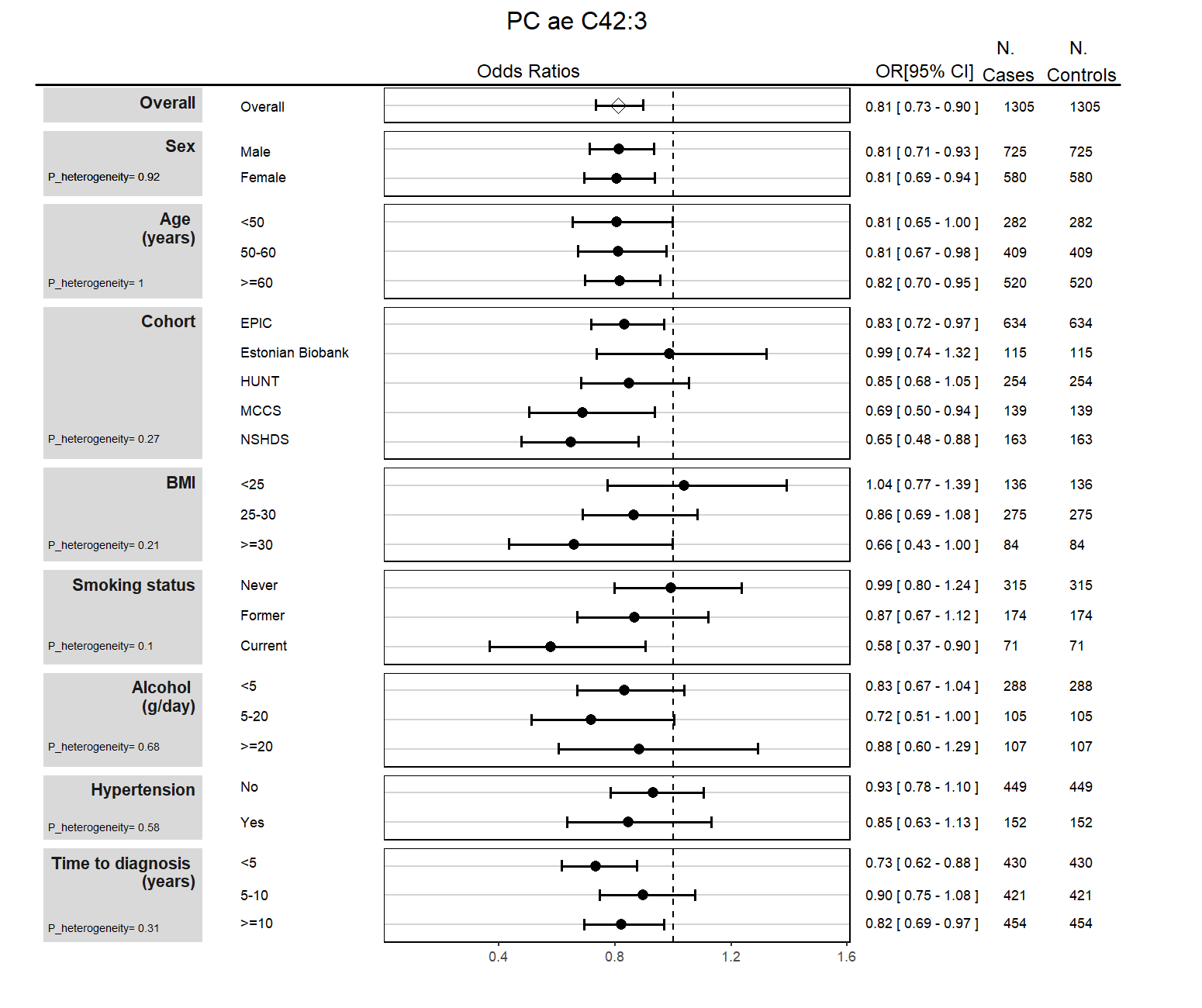
**

BMI: Body Mass Index; CI: Confidence Interval; d: days; g: grams; N.: number of participants; OR: Odds Ratio.

### Figure M. Forest plots depicts the kidney cancer risk association for 1-(1-enyl-palmitoyl)-2-linoleoyl-GPC (P-16:0/18:2)*, stratified by risk factors.

**
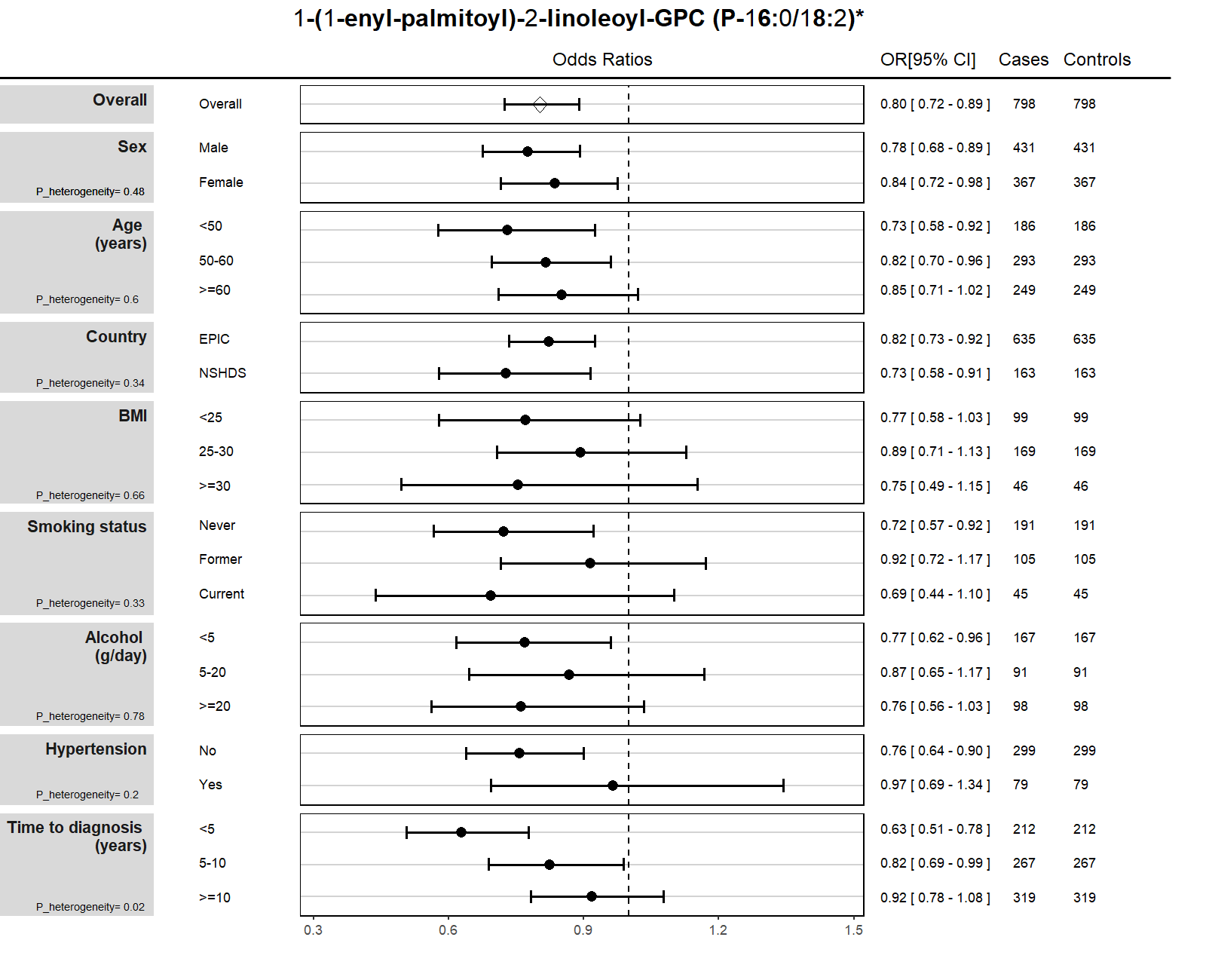
**

BMI: Body Mass Index; CI: Confidence Interval; d: days; g: grams; N.: number of participants; OR: Odds Ratio.

* metabolite identity not yet confirmed by comparison with an authentic chemical standard

### Figure N. Forest plots depicts the kidney cancer risk association for 1-(1-enyl-palmitoyl)-2-oleoyl-GPC (P-16:0/18:1)*, stratified by risk factors.

**
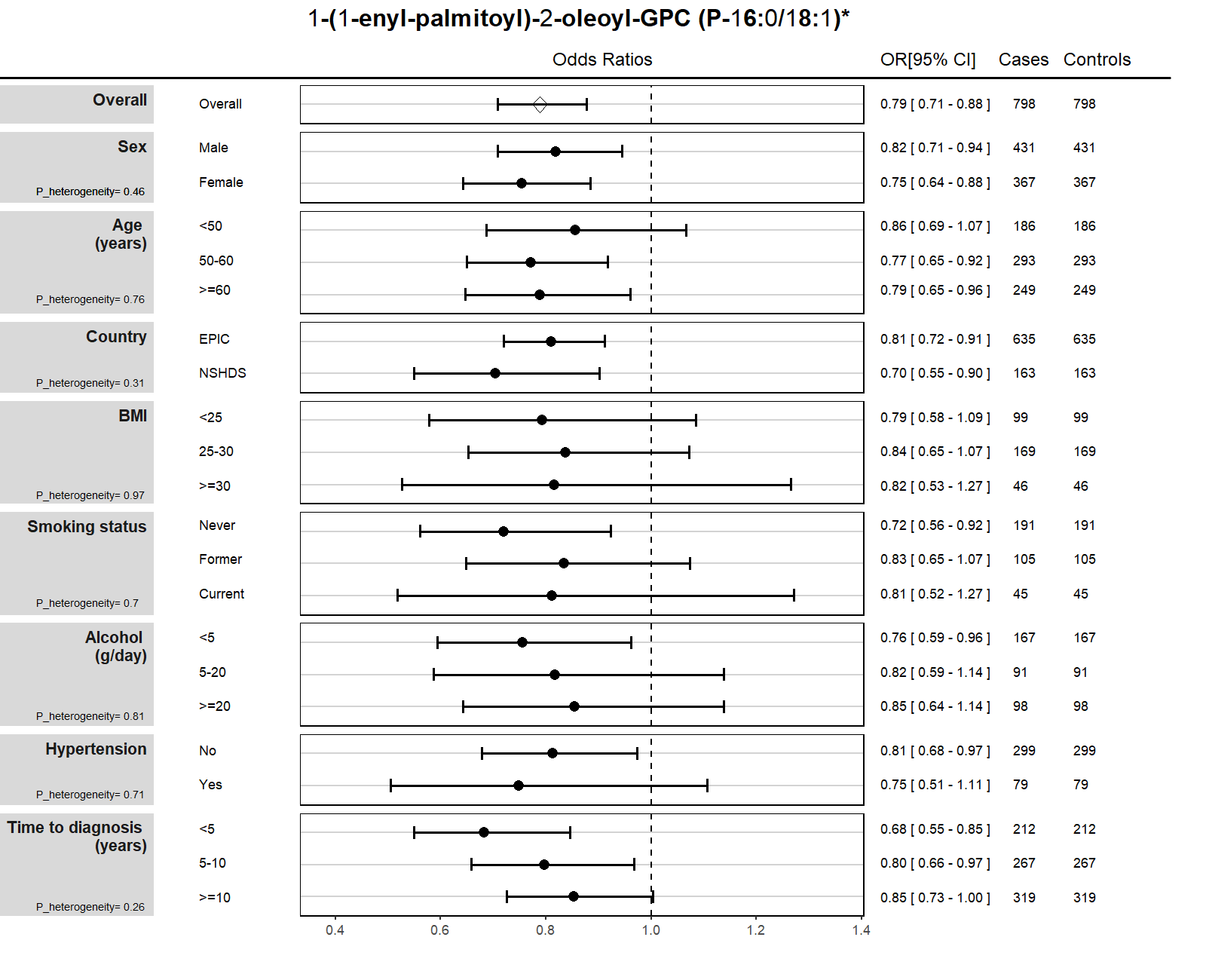
**

BMI: Body Mass Index; CI: Confidence Interval; d: days; g: grams; N.: number of participants; OR: Odds Ratio.

* metabolite identity not yet confirmed by comparison with an authentic chemical standard

### Figure O. Forest plots depicts the kidney cancer risk association for 1-(1-enyl-palmitoyl)-GPC (P-16:0)*, stratified by risk factors.

**
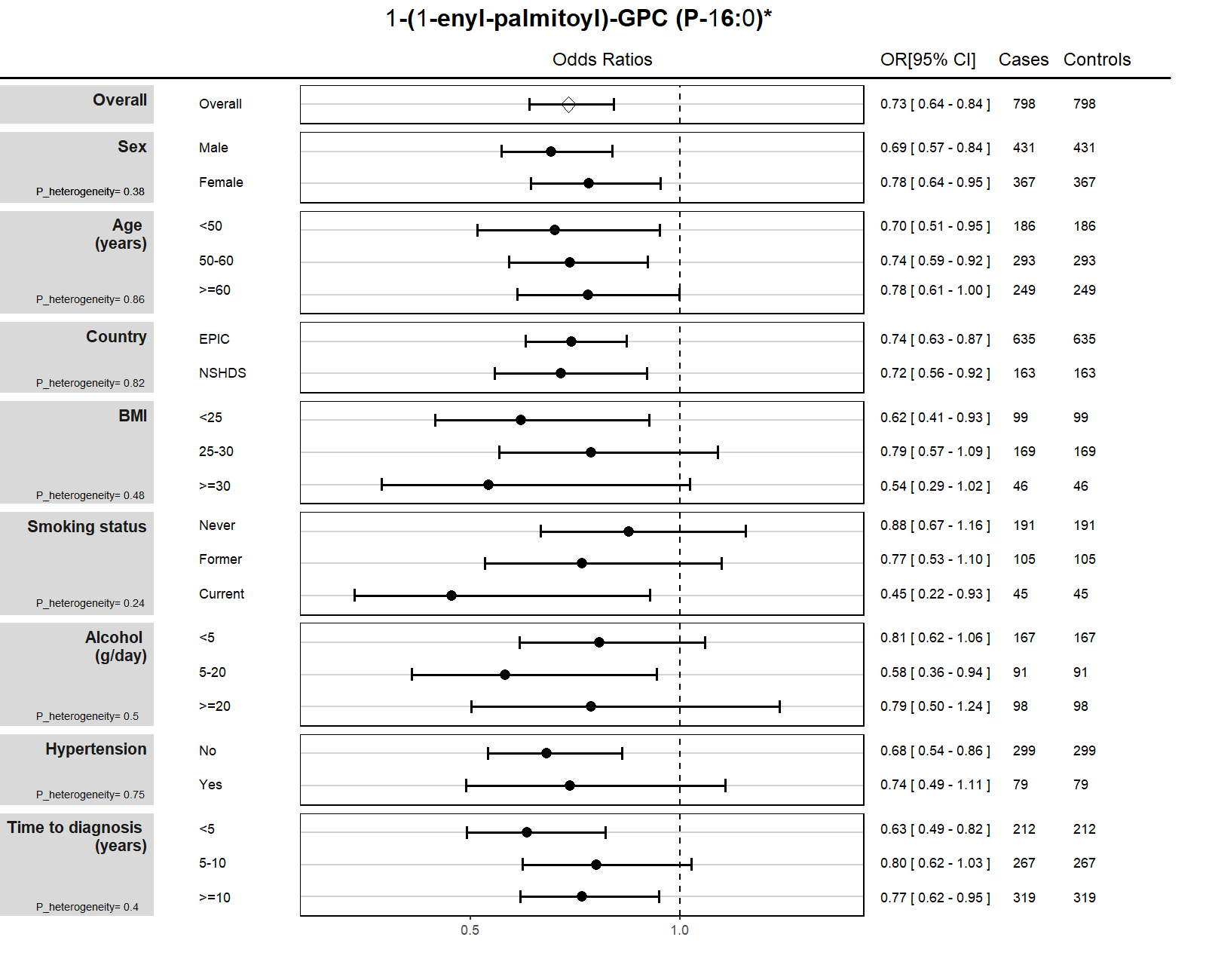
**

BMI: Body Mass Index; CI: Confidence Interval; d: days; g: grams; N.: number of participants; OR: Odds Ratio.

* metabolite identity not yet confirmed by comparison with an authentic chemical standard

### Figure P. Forest plots depicts the kidney cancer risk association for 1-linoleoyl-GPC (18:2), stratified by risk factors.

**
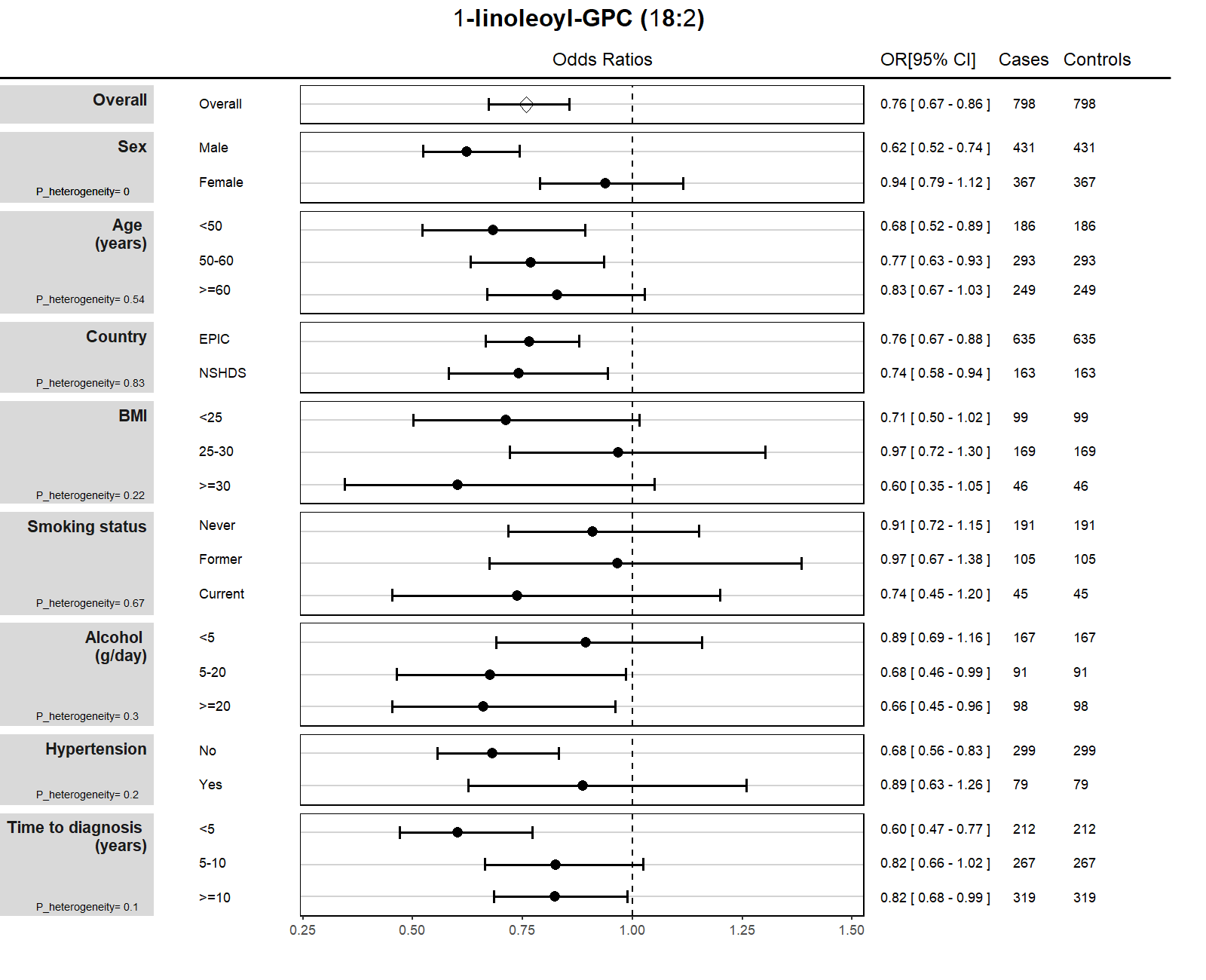
**

BMI: Body Mass Index; CI: Confidence Interval; d: days; g: grams; N.: number of participants; OR: Odds Ratio.

### Figure Q. Forest plots depicts the kidney cancer risk association for beta-cryptoxanthin, stratified by risk factors.

**
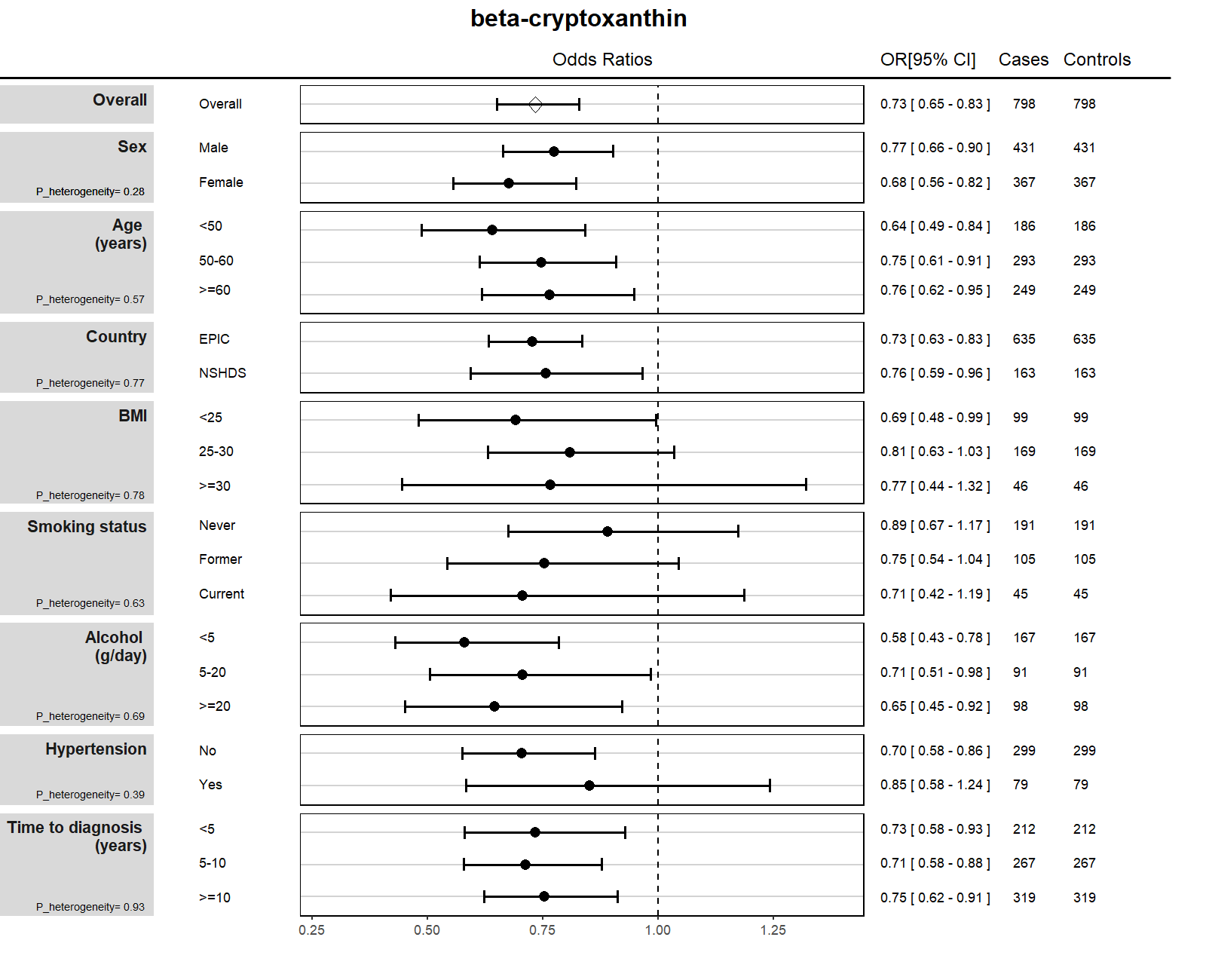
**

BMI: Body Mass Index; CI: Confidence Interval; d: days; g: grams; N.: number of participants; OR: Odds Ratio.

### Figure R. Forest plots depicts the kidney cancer risk association for cysteine-glutathione disulfide, stratified by risk factors.

**
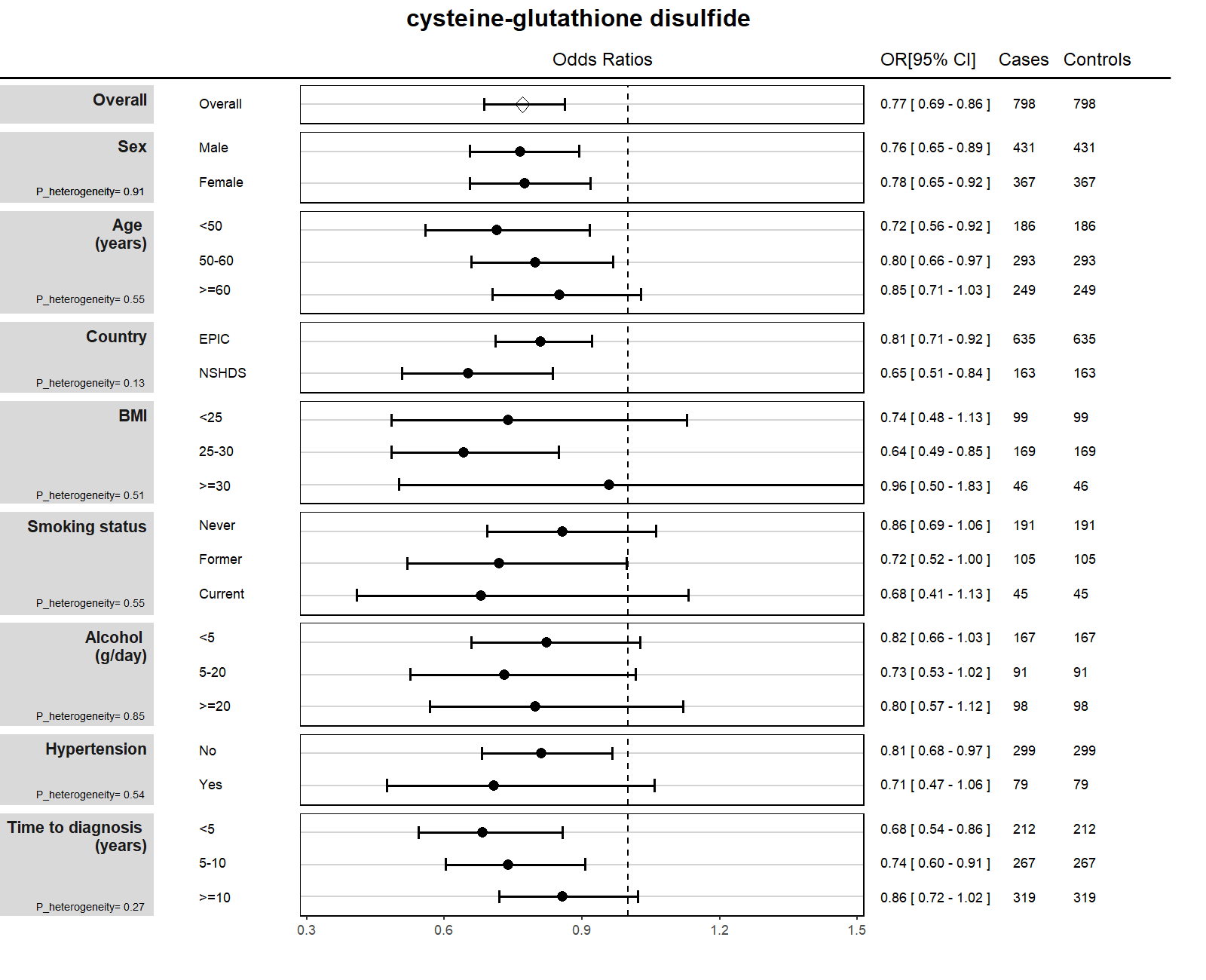
**

BMI: Body Mass Index; CI: Confidence Interval; d: days; g: grams; N.: number of participants; OR: Odds Ratio.

### Figure S. Forest plots depicts the kidney cancer risk association for formiminoglutamate, stratified by risk factors.

**
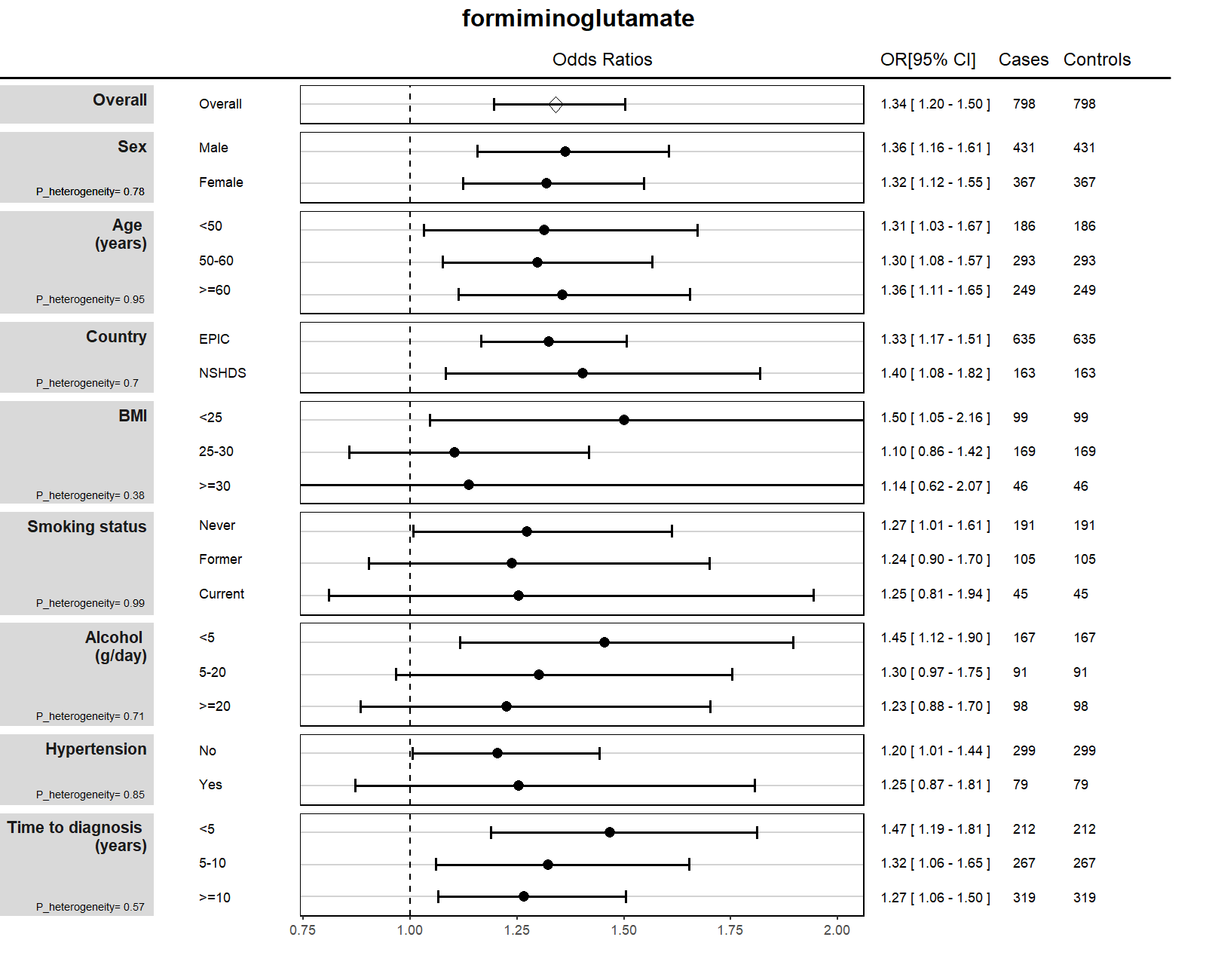
**

BMI: Body Mass Index; CI: Confidence Interval; d: days; g: grams; N.: number of participants; OR: Odds Ratio.

### Figure T. Forest plots depicts the kidney cancer risk association for gamma-glutamylisoleucine*, stratified by risk factors.

**
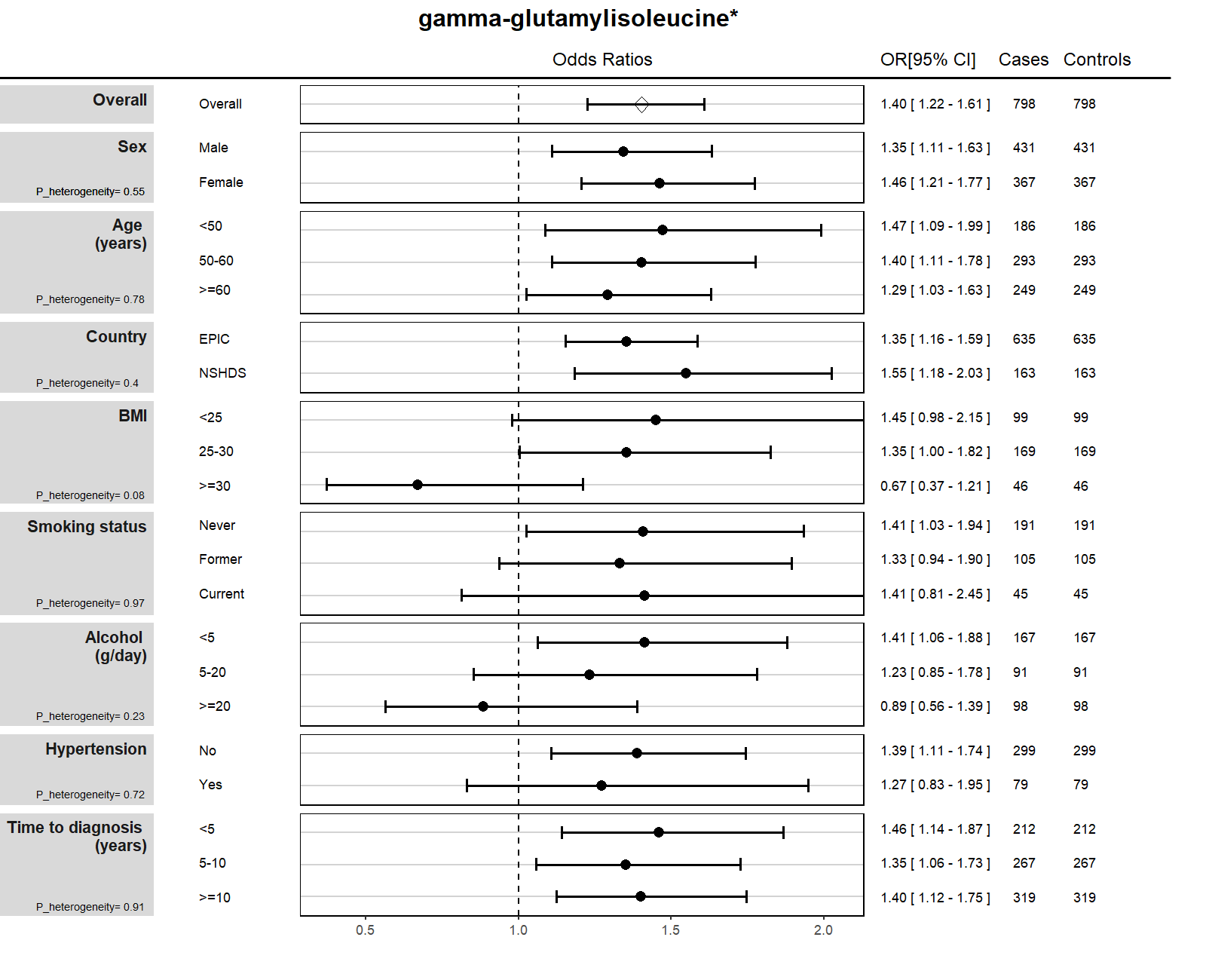
**

BMI: Body Mass Index; CI: Confidence Interval; d: days; g: grams; N.: number of participants; OR: Odds Ratio.

* metabolite identity not yet confirmed by comparison with an authentic chemical standard

### Figure U. Forest plots depicts the kidney cancer risk association for gamma-glutamylvaline, stratified by risk factors.

**
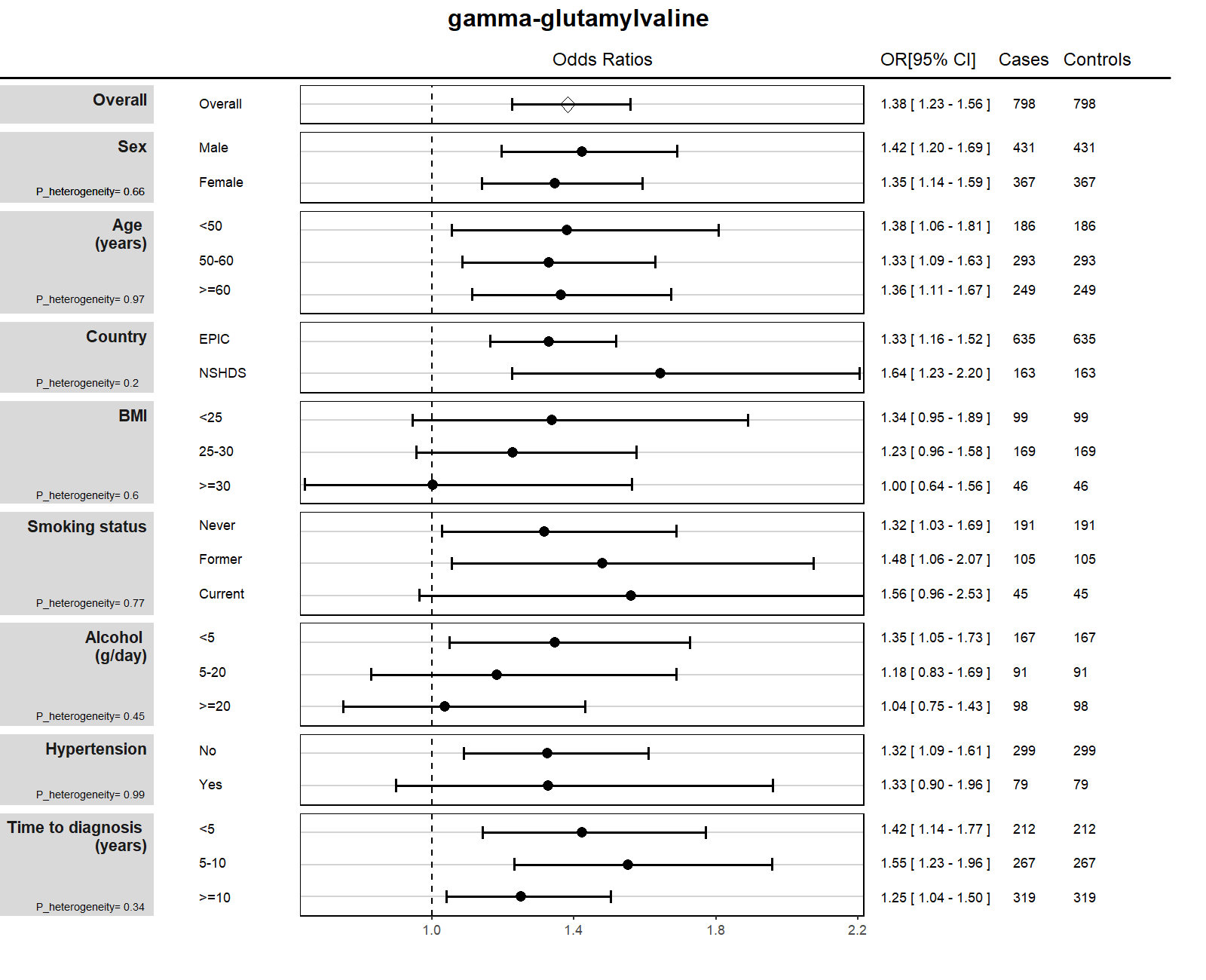
**

BMI: Body Mass Index; CI: Confidence Interval; d: days; g: grams; N.: number of participants; OR: Odds Ratio.

### Figure V. Forest plots depicts the kidney cancer risk association for glutamate (Metabolon), stratified by risk factors.

**
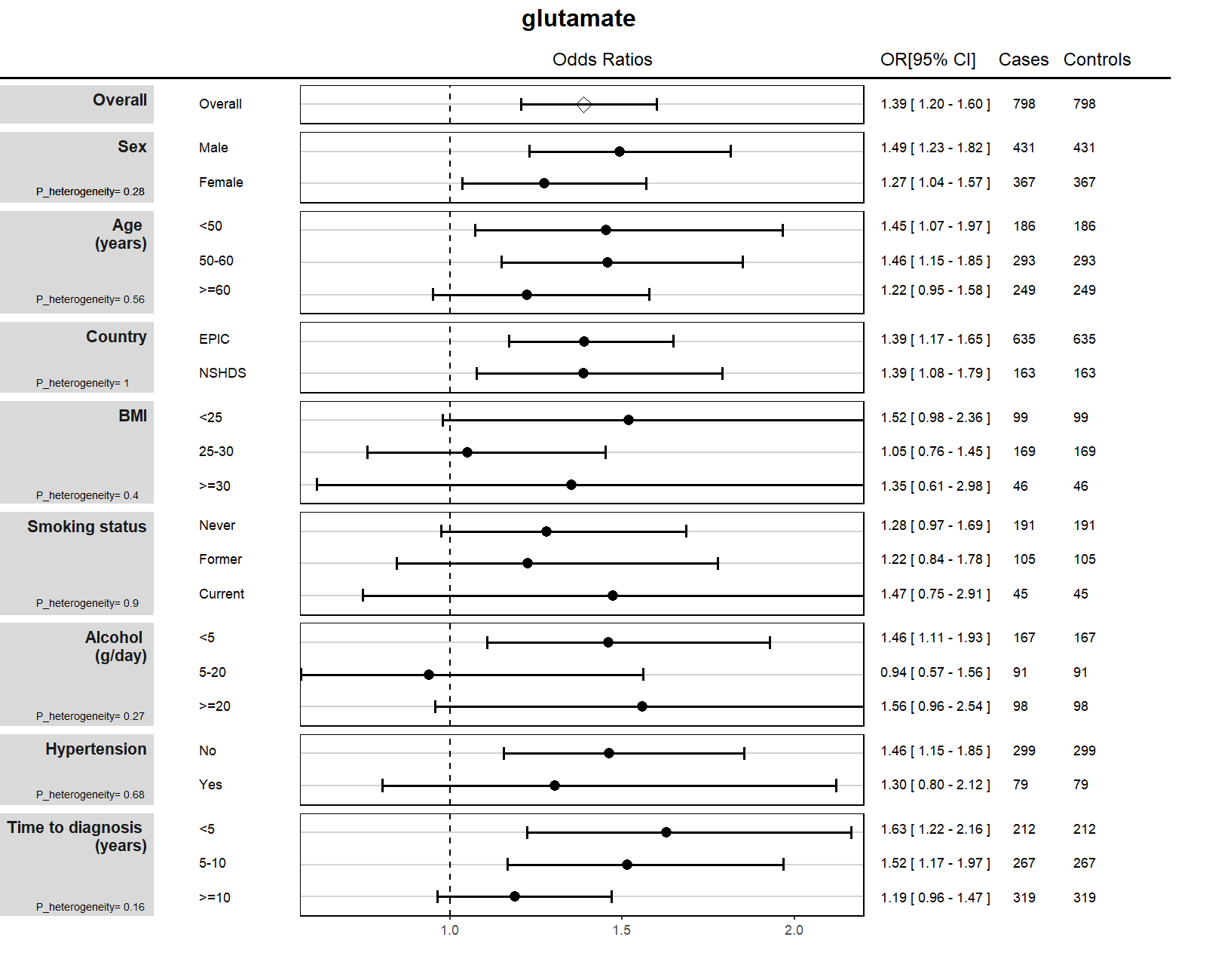
**

BMI: Body Mass Index; CI: Confidence Interval; d: days; g: grams; N.: number of participants; OR: Odds Ratio.

### Figure W. Forest plots depicts the kidney cancer risk association for hydantoin-5-propionate, stratified by risk factors.

**
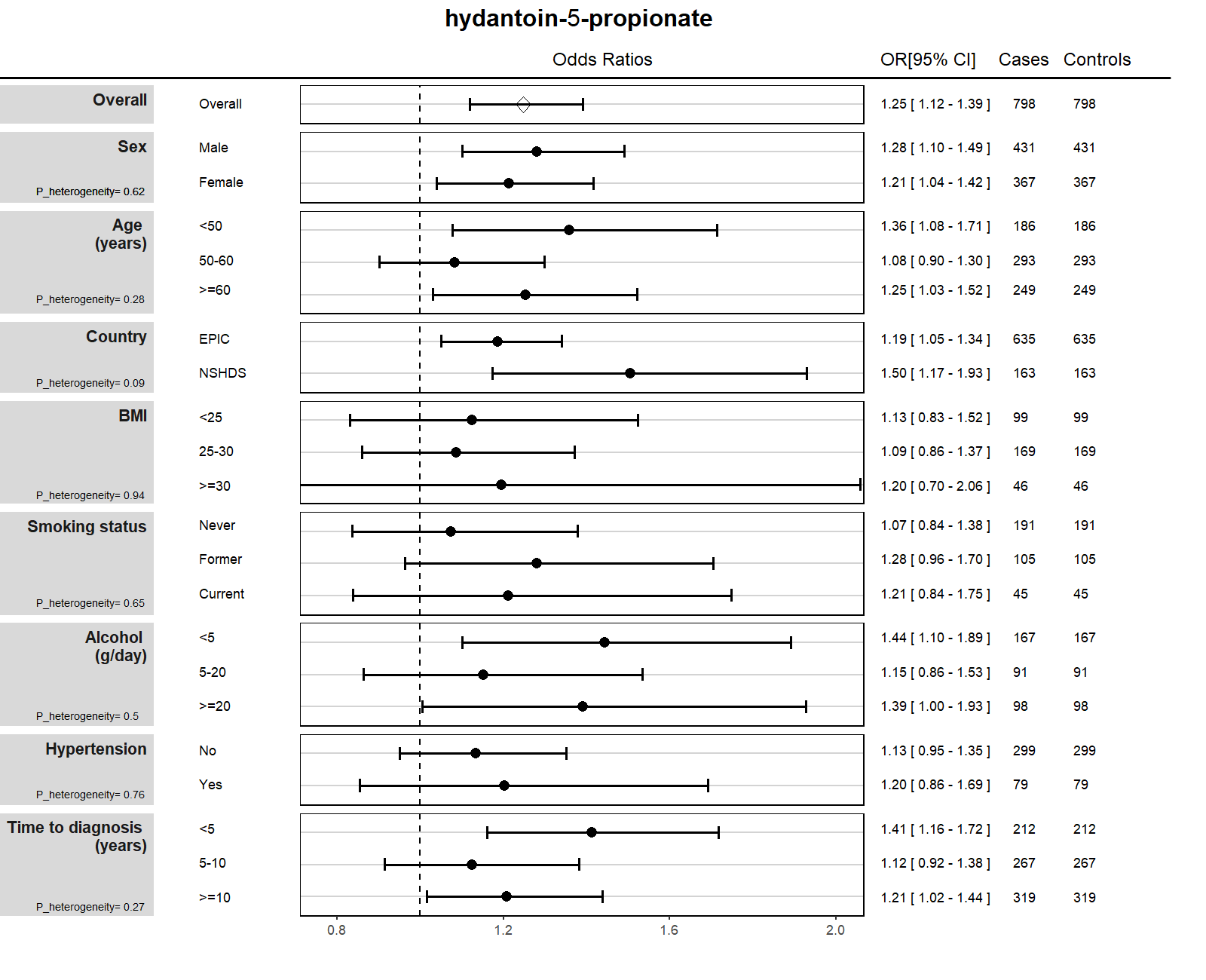
**

BMI: Body Mass Index; CI: Confidence Interval; d: days; g: grams; N.: number of participants; OR: Odds Ratio.

### Figure X. Forest plots depicts the kidney cancer risk association for N1-methyladenosine, stratified by risk factors.

**
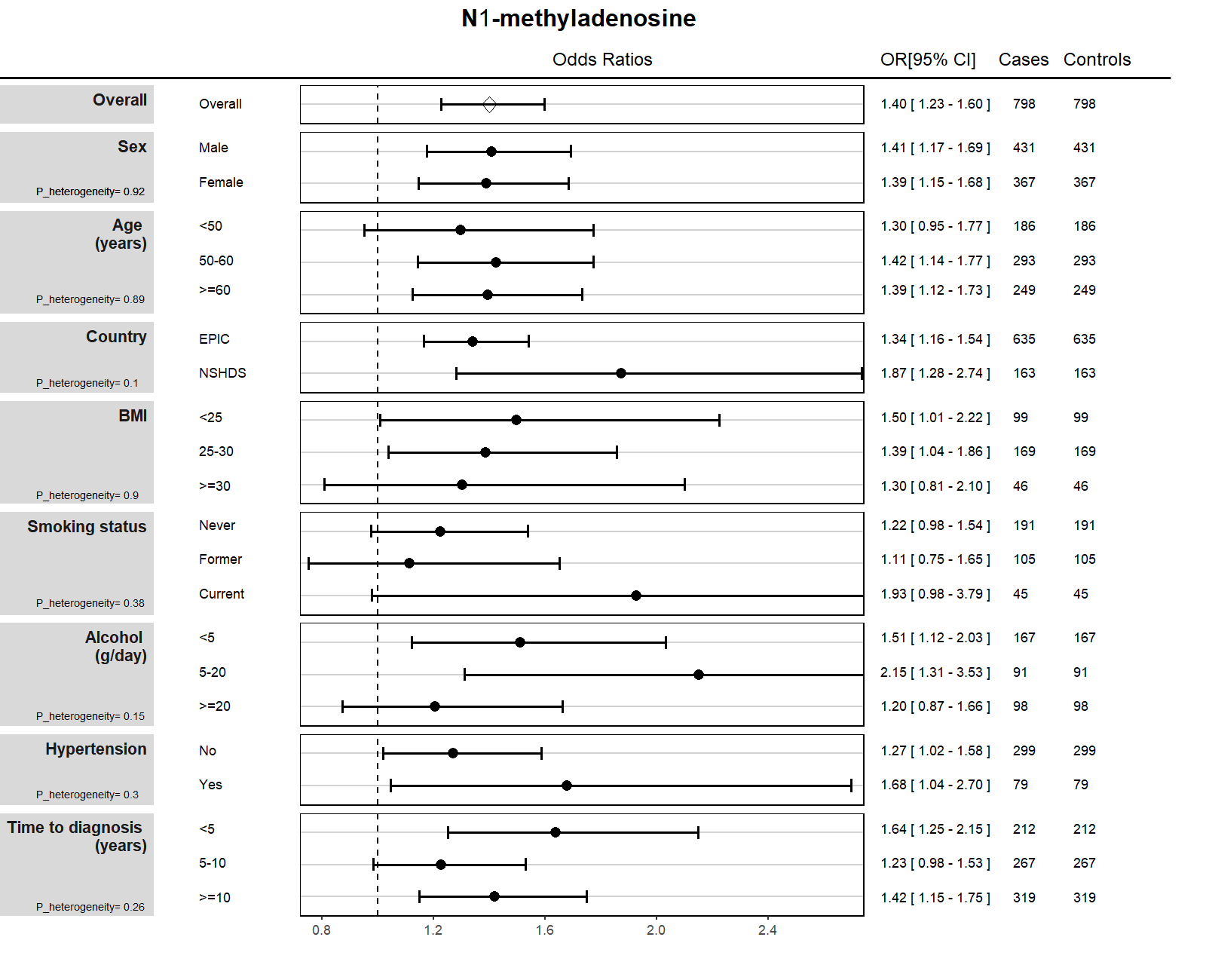
**

BMI: Body Mass Index; CI: Confidence Interval; d: days; g: grams; N.: number of participants; OR: Odds Ratio.

### Figure Y. Forest plots depicts the kidney cancer risk association for X-12096, stratified by risk factors.

**
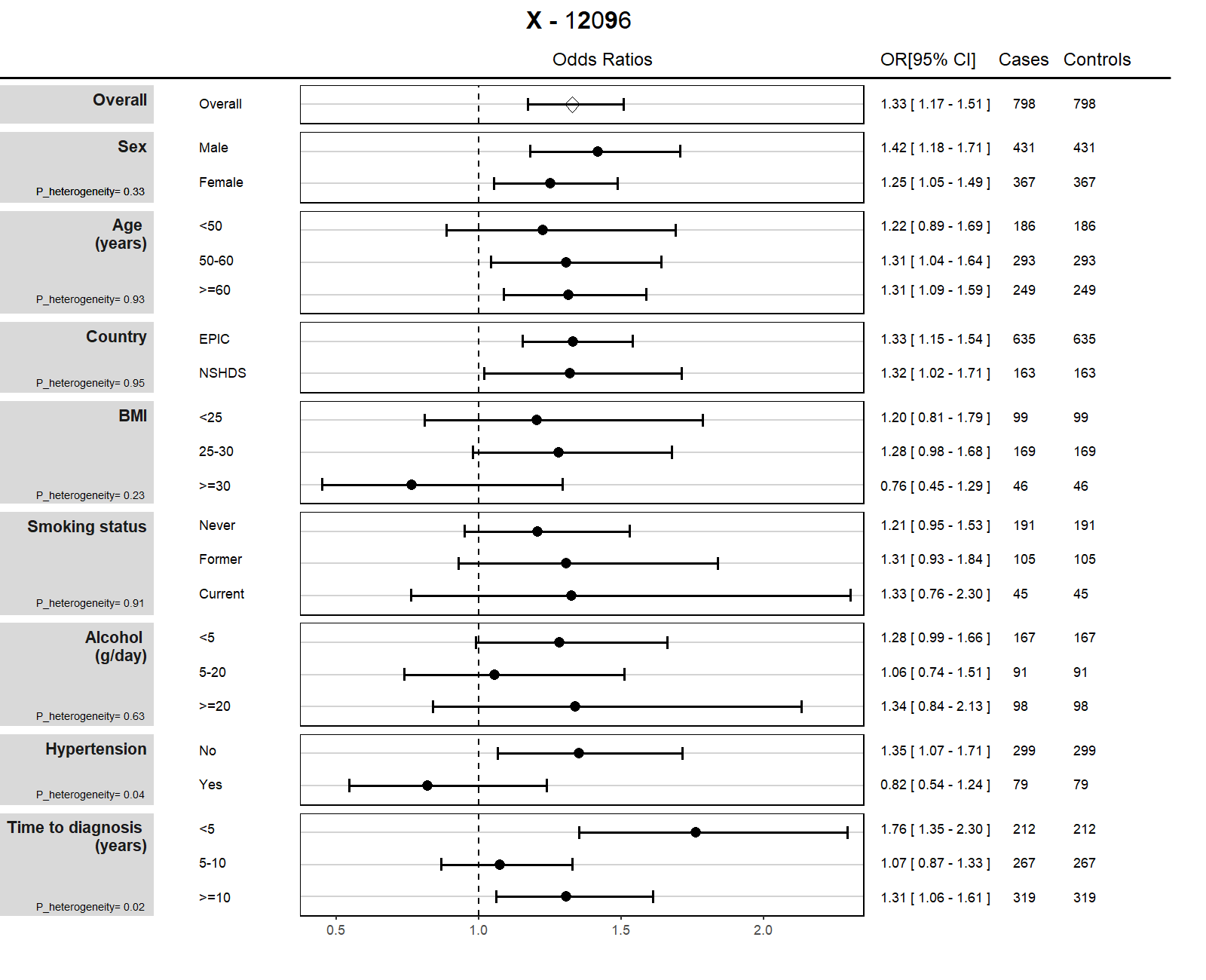
**

BMI: Body Mass Index; CI: Confidence Interval; d: days; g: grams; N.: number of participants; OR: Odds Ratio.
