# Supplementary material for: The blood metabolome of incident kidney cancer: A case–control study nested within the MetKid consortium
Source: PLoS Med. 2021 Sep 20;18(9):e1003786. doi: 10.1371/journal.pmed.1003786 (PMC8496779; doi:10.1371/journal.pmed.1003786)
Supplement: S4 Fig — Scatter plots of the cumulative variance explained in the Metabolon/Biocrates metabolites by the genome-wide significant (p < 5 × 10^−8^) independent (R2 < 0.01) SNPs for the specified risk metabolite (labelled in red). Fig A: Scatter plots of the cumulative variance explained by the genome-wide significant (p < 5 × 10^−8^) independent (R2 < 0.01) SNPs for cysteine-glutathione disulphide (Metabolon). Fig B: Scatter plots of the cumulative variance explained by the genome-wide significant (p < 5 × 10^−8^) independent (R2 < 0.01) SNPs for Hydantoin-5-propionate (Metabolon). Fig C: Scatter plots of the cumulative variance explained by the genome-wide significant (p < 5 × 10^−8^) independent (R2 < 0.01) SNPs for 1-linoleoyl-GPC (18:2) (Metabolon). Fig D: Scatter plots of the cumulative variance explained by the genome-wide significant (p < 5 × 10^−8^) independent (R2 < 0.01) SNPs for 1-(1-enyl-palmitoyl)-GPC (P-16:0) (Metabolon). Fig E: Scatter plots of the cumulative variance explained by the genome-wide significant (p < 5 × 10^−8^) independent (R2 < 0.01) SNPs for 1-(1-enyl-palmitoyl)-2-oleoyl-GPC (P-16:0/18:1) (Metabolon). Fig F: Scatter plots of the cumulative variance explained by the genome-wide significant (p < 5 × 10^−8^) independent (R2 < 0.01) SNPs for 1-(1-enyl-palmitoyl)-2-linoleoyl-GPC (P-16:0/18:2) (Metabolon). Fig G: Scatter plots of the cumulative variance explained by the genome-wide significant (p < 5 × 10^−8^) independent (R2 < 0.01) SNPs for N1-methyladenosine (Metabolon). Fig H: Scatter plots of the cumulative variance explained by the genome-wide significant (p < 5 × 10^−8^) independent (R2 < 0.01) SNPs for PC ae C34:3 (Biocrates). Fig I: Scatter plots of the cumulative variance explained by the genome-wide significant (p < 5 × 10^−8^) independent (R2 < 0.01) SNPs for lysoPC a C18:2 (Biocrates). Fig J: Scatter plots of the cumulative variance explained by the genome-wide significant (p < 5 × 10^−8^) independent (R2 < 0.01) SNPs for PC ae C34:2 (Biocrates). Fig K: Scatte [file pmed.1003786.s008.docx]

**Figures S4. Scatter plots of the cumulative variance explained in the Metabolon/Biocrates metabolites by the genome-wide significant (p<5x10-8) independent (R2<0.01) single nucleotide polymorphisms (SNPs) for the specified risk metabolite (labelled in red)**

[Figure A. Scatter plots of the cumulative variance explained by the genome-wide significant (p<5x10-8) independent (R2<0.01) single nucleotide polymorphisms (SNPs) for cysteine-glutathione disulfide (Metabolon). 2](#_Toc82022921)

[Figure B. Scatter plots of the cumulative variance explained by the genome-wide significant (p<5x10-8) independent (R2<0.01) single nucleotide polymorphisms (SNPs) for Hydantoin-5-propionate (Metabolon). 3](#_Toc82022922)

[Figure C. Scatter plots of the cumulative variance explained by the genome-wide significant (p<5x10-8) independent (R2<0.01) single nucleotide polymorphisms (SNPs) for 1-linoleoyl-GPC (18:2) (Metabolon). 4](#_Toc82022923)

[Figure D. Scatter plots of the cumulative variance explained by the genome-wide significant (p<5x10-8) independent (R2<0.01) single nucleotide polymorphisms (SNPs) for 1-(1-enyl-palmitoyl)-GPC (P-16:0) (Metabolon). 5](#_Toc82022924)

[Figure E. Scatter plots of the cumulative variance explained by the genome-wide significant (p<5x10-8) independent (R2<0.01) single nucleotide polymorphisms (SNPs) for 1-(1-enyl-palmitoyl)-2-oleoyl-GPC (P-16:0/18:1) (Metabolon). 6](#_Toc82022925)

[Figure F. Scatter plots of the cumulative variance explained by the genome-wide significant (p<5x10-8) independent (R2<0.01) single nucleotide polymorphisms (SNPs) for 1-(1-enyl-palmitoyl)-2-linoleoyl-GPC (P-16:0/18:2) (Metabolon). 7](#_Toc82022926)

[Figure G. Scatter plots of the cumulative variance explained by the genome-wide significant (p<5x10-8) independent (R2<0.01) single nucleotide polymorphisms (SNPs) for N1-methyladenosine (Metabolon). 8](#_Toc82022927)

[Figure H. Scatter plots of the cumulative variance explained by the genome-wide significant (p<5x10-8) independent (R2<0.01) single nucleotide polymorphisms (SNPs) for PC ae C34:3 (Biocrates). 9](#_Toc82022928)

[Figure I. Scatter plots of the cumulative variance explained by the genome-wide significant (p<5x10-8) independent (R2<0.01) single nucleotide polymorphisms (SNPs) for lysoPC a C18:2 (Biocrates). 10](#_Toc82022929)

[Figure J. Scatter plots of the cumulative variance explained by the genome-wide significant (p<5x10-8) independent (R2<0.01) single nucleotide polymorphisms (SNPs) for PC ae C34:2 (Biocrates). 11](#_Toc82022930)

[Figure K. Scatter plots of the cumulative variance explained by the genome-wide significant (p<5x10-8) independent (R2<0.01) single nucleotide polymorphisms (SNPs) for lysoPC a C18:1 (Biocrates). 12](#_Toc82022931)

[Figure L. Scatter plots of the cumulative variance explained by the genome-wide significant (p<5x10-8) independent (R2<0.01) single nucleotide polymorphisms (SNPs) for PC ae C40:1 (Biocrates). 13](#_Toc82022932)

[Figure M. Scatter plots of the cumulative variance explained by the genome-wide significant (p<5x10-8) independent (R2<0.01) single nucleotide polymorphisms (SNPs) for PC ae C32:2 (Biocrates). 14](#_Toc82022933)

[Figure N. Scatter plots of the cumulative variance explained by the genome-wide significant (p<5x10-8) independent (R2<0.01) single nucleotide polymorphisms (SNPs) for PC ae C36:3 (Biocrates). 15](#_Toc82022934)

[Figure O. Scatter plots of the cumulative variance explained by the genome-wide significant (p<5x10-8) independent (R2<0.01) single nucleotide polymorphisms (SNPs) for PC ae C42:3 (Biocrates). 16](#_Toc82022935)

[Figure P. Scatter plots of the cumulative variance explained by the genome-wide significant (p<5x10-8) independent (R2<0.01) single nucleotide polymorphisms (SNPs) for PC ae C38:6 (Biocrates). 17](#_Toc82022936)

[Figure Q. Scatter plots of the cumulative variance explained by the genome-wide significant (p<5x10-8) independent (R2<0.01) single nucleotide polymorphisms (SNPs) for PC aa C42:1 (Biocrates) 18](#_Toc82022937)

Figure A. Scatter plots of the cumulative variance explained by the genome-wide significant (p<5x10-8) independent (R2<0.01) single nucleotide polymorphisms (SNPs) for cysteine-glutathione disulfide (Metabolon).

Metabolites that are labelled have a p value below the genome-wide significance threshold (p<5E-08).

### Figure B. Scatter plots of the cumulative variance explained by the genome-wide significant (p<5x10-8) independent (R2<0.01) single nucleotide polymorphisms (SNPs) for **Hydantoin-5-propionate (Metabolon).**

Metabolites that are labelled have a p value below the genome-wide significance threshold (p<5E-08).

### **Figure C.** Scatter plots of the cumulative variance explained by the genome-wide significant (p<5x10-8) independent (R2<0.01) single nucleotide polymorphisms (SNPs) for **1-linoleoyl-GPC (18:2) (Metabolon).**

Metabolites that are labelled have a p value below the genome-wide significance threshold (p<5E-40).

### **Figure D.** Scatter plots of the cumulative variance explained by the genome-wide significant (p<5x10-8) independent (R2<0.01) single nucleotide polymorphisms (SNPs) for **1-(1-enyl-palmitoyl)-GPC (P-16:0) (Metabolon).**

Metabolites that are labelled have a p value below the genome-wide significance threshold (p<5E-50).

### Figure E. Scatter plots of the cumulative variance explained by the genome-wide significant (p<5x10-8) independent (R2<0.01) single nucleotide polymorphisms (SNPs) for 1-(1-enyl-palmitoyl)-2-oleoyl-GPC (P-16:0/18:1) (Metabolon).

Metabolites that are labelled have a p value below the genome-wide significance threshold (p<5E-20).

### Figure F. Scatter plots of the cumulative variance explained by the genome-wide significant (p<5x10-8) independent (R2<0.01) single nucleotide polymorphisms (SNPs) for 1-(1-enyl-palmitoyl)-2-linoleoyl-GPC (P-16:0/18:2) **(Metabolon).**

Metabolites that are labelled have a p value below the genome-wide significance threshold (p<5E-20).

### Figure G. Scatter plots of the cumulative variance explained by the genome-wide significant (p<5x10-8) independent (R2<0.01) single nucleotide polymorphisms (SNPs) for N1-methyladenosine **(Metabolon).**

Metabolites that are labelled have a p value below the genome-wide significance threshold (p<5E-08).

### Figure H. Scatter plots of the cumulative variance explained by the genome-wide significant (p<5x10-8) independent (R2<0.01) single nucleotide polymorphisms (SNPs) for PC ae C34:3 (Biocrates).

Metabolites that are labelled have a p value below the genome-wide significance threshold (p<5E-08).

### Figure I. Scatter plots of the cumulative variance explained by the genome-wide significant (p<5x10-8) independent (R2<0.01) single nucleotide polymorphisms (SNPs) for lysoPC a C18:2 (Biocrates).

Metabolites that are labelled have a p value below the genome-wide significance threshold (p<5E-08). ****

### Figure J. Scatter plots of the cumulative variance explained by the genome-wide significant (p<5x10-8) independent (R2<0.01) single nucleotide polymorphisms (SNPs) for PC ae C34:2 (Biocrates).

Metabolites that are labelled have a p value below the genome-wide significance threshold (p<5E-20).

### Figure K. Scatter plots of the cumulative variance explained by the genome-wide significant (p<5x10-8) independent (R2<0.01) single nucleotide polymorphisms (SNPs) for lysoPC a C18:1 (Biocrates).

Metabolites that are labelled have a p value below the genome-wide significance threshold (p<5E-08).

### Figure L. Scatter plots of the cumulative variance explained by the genome-wide significant (p<5x10-8) independent (R2<0.01) single nucleotide polymorphisms (SNPs) for PC ae C40:1 (Biocrates).

Metabolites that are labelled have a p value below the genome-wide significance threshold (p<5E-10).

### Figure M. Scatter plots of the cumulative variance explained by the genome-wide significant (p<5x10-8) independent (R2<0.01) single nucleotide polymorphisms (SNPs) for PC ae C32:2 (Biocrates).

Metabolites that are labelled have a p value below the genome-wide significance threshold (p<5E-08).

### Figure N. Scatter plots of the cumulative variance explained by the genome-wide significant (p<5x10-8) independent (R2<0.01) single nucleotide polymorphisms (SNPs) for PC ae C36:3 (Biocrates).

Metabolites that are labelled have a p value below the genome-wide significance threshold (p<5E-08).

### Figure O. Scatter plots of the cumulative variance explained by the genome-wide significant (p<5x10-8) independent (R2<0.01) single nucleotide polymorphisms (SNPs) for PC ae C42:3 (Biocrates).

Metabolites that are labelled have a p value below the genome-wide significance threshold (p<5E-08).

### Figure P. Scatter plots of the cumulative variance explained by the genome-wide significant (p<5x10-8) independent (R2<0.01) single nucleotide polymorphisms (SNPs) for PC ae C38:6 (Biocrates).

Metabolites that are labelled have a p value below the genom e-wide significance threshold (p<5E-08).

### Figure Q. Scatter plots of the cumulative variance explained by the genome-wide significant (p<5x10-8) independent (R2<0.01) single nucleotide polymorphisms (SNPs) for PC aa C42:1 (Biocrates)

Metabolites that are labelled have a p value below the genome-wide significance threshold (p<5E-08).
