# Supplementary material for: The blood metabolome of incident kidney cancer: A case–control study nested within the MetKid consortium
Source: PLoS Med. 2021 Sep 20;18(9):e1003786. doi: 10.1371/journal.pmed.1003786 (PMC8496779; doi:10.1371/journal.pmed.1003786)
Supplement: S5 Fig — BMI, body mass index; MR, mendelian randomisation. (DOCX) [file pmed.1003786.s009.docx]

**Figure S5. Volcano plots representing the association between BMI and circulating Biocrates metabolites (triangle) and Metabolon metabolites (dots) from MR analyses.**

Metabolites that are labelled have a *p* value below the significance threshold (*p<*0.0003 for Biocrates and *p<*5.48x10-5 for Metabolon*).*

BMI: Body Mass Index

* metabolite identity not yet confirmed by comparison with an authentic chemical standard
