# Supplementary material for: The blood metabolome of incident kidney cancer: A case–control study nested within the MetKid consortium
Source: PLoS Med. 2021 Sep 20;18(9):e1003786. doi: 10.1371/journal.pmed.1003786 (PMC8496779; doi:10.1371/journal.pmed.1003786)
Supplement: S6 Fig — MR, mendelian randomisation. (DOCX) [file pmed.1003786.s010.docx]

**Figure S6. Scatter plots comparing the metabolite profile associated with kidney cancer from prospective observational analyses with the dental disease-driven metabolite profile from MR analyses.**

Z score was calculated by dividing the effect estimate (log OR or beta) by the standard errors. Metabolites that are labelled have a *p* value below the threshold (*p<*0.05/Effective number of tests (ENT)) in the pooled analyses and are nominally significant in at least 2 cohorts separately. Metabolites measured by the Biocrates platform that are below the *p* value threshold are represented by triangles, those measured by the Metabolon platform that are below the *p* value threshold are represented by dots and those that are measured by either the Biocrates or the Metabolon platform that are above the *p* value threshold are represented by an x.

MR: Mendelian Randomization; OR: Odds Ratio; SE: Standard Error.

* metabolite identity not yet confirmed by comparison with an authentic chemical standard

On the y-axis, the OR and SE were derived from the logistic regression analyses conditioned on case set estimating the associations between circulating metabolites and kidney cancer risk in five prospective cohorts.

On the x-axis, the beta and SE were derived from the mendelian randomization analyses evaluating the effect of BMI on circulating metabolites levels.
